# Discovery of parvovirus-related sequences in an unexpected broad range of animals

**DOI:** 10.1038/srep30880

**Published:** 2016-09-07

**Authors:** S. François, D. Filloux, P. Roumagnac, D. Bigot, P. Gayral, D. P. Martin, R. Froissart, M. Ogliastro

**Affiliations:** 1 INRA, UMR DGIMI, F-34095, Montpellier, France; 2 CIRAD-INRA-SupAgro, UMR BGPI, Campus International de Montferrier-Baillarguet, Montpellier Cedex-5, France; 3 Institut de Recherche sur la Biologie de l’Insecte, UMR 7261, CNRS–Université François Rabelais, 37200 Tours, France; 4 UMR5554–Institut des Sciences de l’Evolution UMR5554, Université Montpellier–CNRS–IRD–EPHE, 34000 Montpellier, France; 5 Computational Biology Group, Institute of Infectious Disease and Molecular Medicine, Faculty of Health Sciences, University of Cape Town, Observatory, South Africa; 6 CNRS-IRD-UM, UMR 5290, MIVEGEC, 911 avenue Agropolis, 34394, Montpellier, France

## Abstract

Our knowledge of the genetic diversity and host ranges of viruses is fragmentary. This is particularly true for the *Parvoviridae* family. Genetic diversity studies of single stranded DNA viruses within this family have been largely focused on arthropod- and vertebrate-infecting species that cause diseases of humans and our domesticated animals: a focus that has biased our perception of parvovirus diversity. While metagenomics approaches could help rectify this bias, so too could transcriptomics studies. Large amounts of transcriptomic data are available for a diverse array of animal species and whenever this data has inadvertently been gathered from virus-infected individuals, it could contain detectable viral transcripts. We therefore performed a systematic search for parvovirus-related sequences (PRSs) within publicly available transcript, genome and protein databases and eleven new transcriptome datasets. This revealed 463 PRSs in the transcript databases of 118 animals. At least 41 of these PRSs are likely integrated within animal genomes in that they were also found within genomic sequence databases. Besides illuminating the ubiquity of parvoviruses, the number of parvoviral sequences discovered within public databases revealed numerous previously unknown parvovirus-host combinations; particularly in invertebrates. Our findings suggest that the host-ranges of extant parvoviruses might span the entire animal kingdom.

Recent studies have shown that viruses are the most numerous and diverse genetic entities on Earth: a discovery that has completely changed both our views on their prevalence, and our perception that they are primarily disease-causing agents^1^. Viruses have been discovered infecting organisms throughout the entire tree of life, using a wide array of strategies to move between and infect hosts belonging to either the same or different species. The genomes of many viruses can also ligate to, and become a heritable part of, the genetic material of their hosts^2^.

Largely because of their obvious medical and economic importance, the vast majority of viruses that have so far been studied are those that cause recognizable diseases of humans and our domesticated plants (almost exclusively angiosperms) and animals (mainly mammals and birds)^3^,^4^. One of the greatest achievements of environmental metagenomics has been the discovery that unknown viral species vastly outnumber the known species, and that there probably also remain more unknown genera (and possibly also entire families) than those we have currently discovered^4^,^5^. For example, it is now estimated that the ~2800 virus species that are currently recognised by the International Committee on Taxonomy of Viruses^6^ (ICTV probably account for less than 1% of all viral species on Earth^7^. Our rapidly expanding appreciation of the actual diversity of viruses on Earth is well illustrated in the recent discoveries of viruses with ssDNA genomes that likely belong to multitudes of novel genera/families which are both genetically highly divergent from species in the known ssDNA virus families (such as parvoviruses, circoviruses, microviruses and geminiviruses) and likely infect hosts that span the entire tree of life^8^,^9^,^10^,^11^,^12^,^13^,^14^.

Parvoviruses illustrate the chasm that likely exists between the known diversity of species within particular virus families, and that which actually exists in all of their potential animal hosts. The linear ssDNA viruses belonging to the family *Parvoviridae* are presently divided into two sub-families: the *Parvovirinae*, which contains species infecting vertebrates (which have, to date, mostly been birds and mammals), and the *Densovirinae*, which contains species infecting arthropods^10^ (which have, to date, mostly been crustaceans and insects). Parvovirus genomes are characteristically 4 to 6 kb long, with inverted terminal repeats (ITRs) that bracket two sets of genes encoding non-structural (Rep or NS) and structural (VP) proteins^15^. While degrees of sequence identity between viruses from different parvovirus genera are very low (e.g. some pairs of densoviruses share <15% VP sequence identity), most parvoviruses likely express both a NS1 protein with a super family 3 helicase (SF3) domain in the C terminus and a VP containing a unique phospholipase A2 (PLA2) motif in the N terminus^16^,^17^. These two proteins, even if the PLA2 motif is missing in some parvoviruses^16^, are therefore useful for parvovirus phylogenetic inferences.

Our current appreciation of parvovirus diversity is limited to the 41 *Parvovirinae* and 15 *Densovirinae* species which are presently recognized by the ICTV^18^. We estimated that only a few hundred animal species have been reported as hosts of parvoviruses, representing only approximately 0.0001% of the 1.2 million animal species that have presently been described^19^. There is likely a particularly extreme imbalance of sampling between the vertebrate-infecting *Parvovirinae* and the arthropod-infecting *Densovirinae* in that there are likely almost 20 times more arthropod species on Earth than there are species of vertebrates^19^. Given the diversity in sequence and genome organisation displayed by parvoviruses, it is entirely plausible that parvoviruses are ubiquitous in the environment, and that there exist tens of thousands of undiscovered vertebrate parvoviruses, and hundreds of thousands of undiscovered arthropod parvoviruses. Indeed, high throughput sequencing technologies and the advent of routine whole genome sequencing of eukaryotes have revealed the occurrence of “fossil” parvovirus sequences integrated into the genomes of multiple animals including those of humans. While these integrated sequences reflect a history of parvovirus interactions with an unexpectedly broad range of animal hosts^20^,^21^, the recent discovery of a densovirus associated with sea-star wasting disease supports the hypothesis that the host range of extant parvoviruses probably extends far beyond the major animal phyla in which they have already been detected^16^. This discovery also raises questions about whether densoviruses are predisposed to frequently shifting hosts, or whether they may have existed and co-evolved with their hosts during the early evolutionary radiation of multi-cellular animals^22^,^23^.

Parvoviruses belonging to the *Dependovirus* genus may depend on another virus to complete their replication-deficient life cycle. These viruses can be persistent and include a host genome integration step that results in latent infections, as has been shown for *Adeno-Associated-Virus* (AAV) where integration requires the NS1 homologue, Rep^24^. Integration and persistence of densoviruses may in some cases even be beneficial for their hosts in that it could protect them against viral infections^25^,^26^.

We therefore hypothesised that while transcriptome datasets might contain evidence of parvovirus sequences originating either from either *bona fide* transmissible episomal viruses, or from integrated (albeit possibly only transiently) viruses or viral sequence elements (from heritably integrated but still transcriptionally active parvovirus genes), animal genome sequences might contain evidence of heritably integrated, and possibly transcriptionally dormant, parvovirus sequence elements. Crucially, large volumes of transcriptomic and genomic sequence data for a wide array of animal species are currently available in public databases. Several studies have already revealed the presence of a variety of novel RNA and DNA virus sequences within transcriptome datasets^25^,^26^,^27^,^28^ (including those of animal-model organisms and environmental transcriptomes). We opted to initially focus our search for novel parvoviruses within transcriptome datasets from animals covering vertebrate and invertebrate phyla, spanning the entire animal kingdom and representing an extend of in depth searches that has not been achieved so far for parvoviruses.

The results presented here highlight the extraordinary diversity, abundance and ubiquity of expressed parvoviral sequences in numerous animal phyla, revealing previously unknown parvovirus-host associations–particularly with invertebrates including arthropods, molluscs, annelids, nematodes, and cnidarians–and supporting the hypothesis that the collective host ranges of extant parvoviruses might indeed span the entire animal kingdom.

## Results

### Identification of parvovirus-related sequences in animal transcriptome datasets

We used 74 representative parvovirus genomes as queries (Supplementary Table S1) to perform BLASTX searches against the National Centre for Biotechnological Information (NCBI) non-redundant (nr) cDNA expressed sequence tag (EST), transcriptome shotgun assembly (TSA) and protein (Uniprot) databases^29^,^30^. We also used these as queries to screen, eleven new transcriptomes generated from invertebrate datasets provided by N. Galtier (European Research Council advanced grant 232971 (PopPhyl)). All hits were next selected and used as queries to perform BLASTX or BLASTP reciprocal searches of the NCBI non-redundant sequence database (as described in the materials and methods).

**Figure 1:**
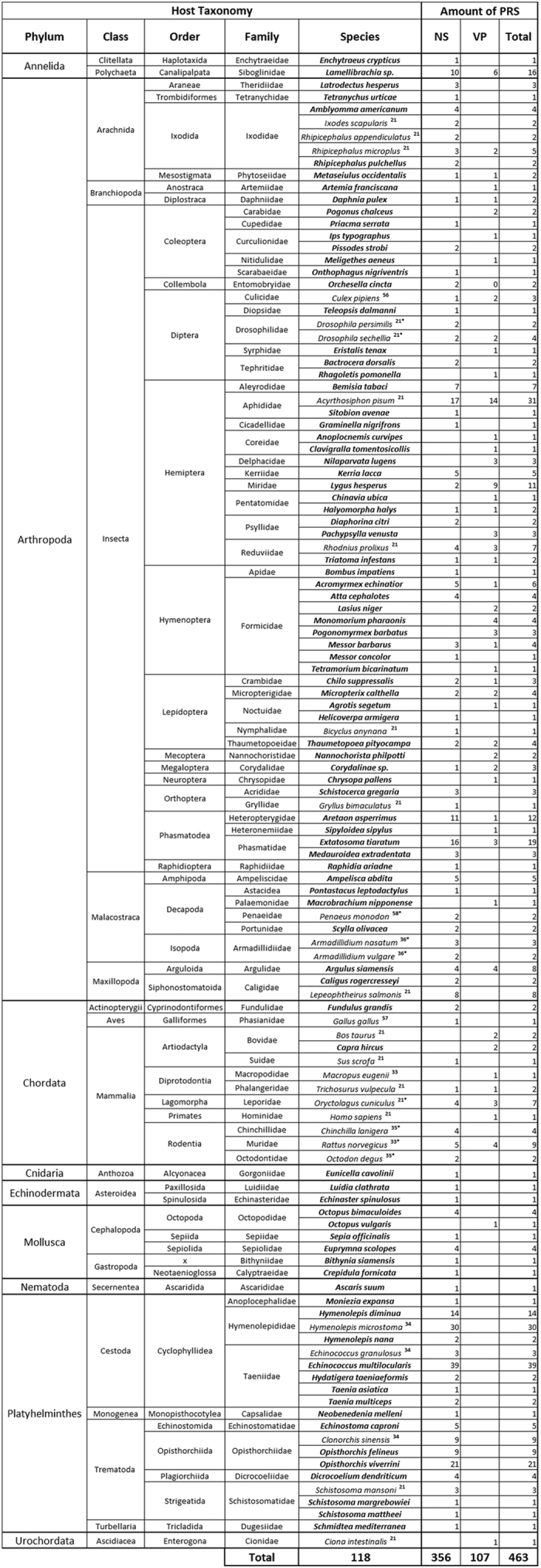
Distribution of PRSs among animals. VP and NS refer to viral structural and non-structural proteins respectively. PRSs were identified using the full sequences of 74 representative parvovirus genomes (provided in Table S1) as queries to search the EST (Expressed Sequence Tags), TSA (Transcriptome Shotgun Assembly), nr Nucleotide collection, and Uniprot databases as well as search in 11 un-deposited transcriptomes either produced for this study (*Lamellibrachia spp*.) or already published in another context (*Artemia franciscana*, *Culex pipiens*, *Crepidula fornicata*, *Eunicella cavolinii* and *Messor barbarus and Messor concolor*)^40^,^41^. Animal species wherein PRSs were first identified in this study are represented in bold, while PRSs that have already been identified are associated with numbers corresponding to the respective references. (*) PRS endogenization has been confirmed by PCRs.

Three hundred and fifty-six homologues of parvoviral non-structural protein (NS) sequences and 107 homologues of capsid protein (VP) sequences, from partial to near complete coding sequences (Fig. 1 and Supplementary Table S2), were recovered from the NCBI transcriptome (230 sequences) and genome (206 sequences) databases and from the 11 new invertebrate transcriptome datasets (27 sequences found in 8/11 datasets). These 463 PRSs were found from the transcriptomes and genomes of 118 animal species, including 74 arthropods, 19 platyhelminthes, 12 vertebrates, six molluscs, two echinoderms, two annelids, one tunicate, one nematode, and one cnidarian (Fig. 1).

Overall, 89 potentially new parvovirus host species were identified, including species belonging to animal phyla with no previously known parvovirus host species: *Mollusca*, *Annelida*, *Nematoda* and *Cnidaria* (Fig. 1). Whereas the 88% of PRSs that were recovered from 95 invertebrates (including 69 arthropods) were more similar to viruses belonging to the arthropod-infecting *Densovirinae* subfamily, the remaining 12% of PRSs that were recovered from 12 vertebrate species and 4 molluscs were more similar to vertebrate-infecting viruses in the *Parvovirinae* subfamily (Supplementary Table S2). Among arthropods, PRSs were found in species within classes (e.g. *Branchiopoda* and *Arachnida*), orders (*Phasmatodea* and *Coleoptera*) and families (e.g. *Formicidae*) that contained no previously identified parvovirus hosts (PRSs are summarized in Table S2).

It is also noteworthy that several copies of PRSs homologous to both NS and VP encoding genes were found in 72/118 of the animal transcriptomes (Table S2).

Most of the 463 PRSs (77%, corresponding to 79 animal species) potentially encoded proteins sharing between 30–85% aa identity to the NS or VP proteins of a known extant parvovirus, while 17% (derived from 30 animal species) potentially encoded proteins sharing less than 30% identity to any known extant parvoviruses. This degree of similarity is below the parvovirus genus demarcation threshold recommended by the ICTV (i.e. >30% amino acid identity in NS1), suggesting that, if these divergent PRSs are derived from extant viruses, these likely belong to species within as yet uncharacterized parvovirus genera^14^. Finally, 6% of the PRSs found within the transcriptome datasets of nine animal species potentially encoded proteins with more than 85% identity to those expressed by known extant parvoviruses (Supplementary Table S2).

**Figure 2:**
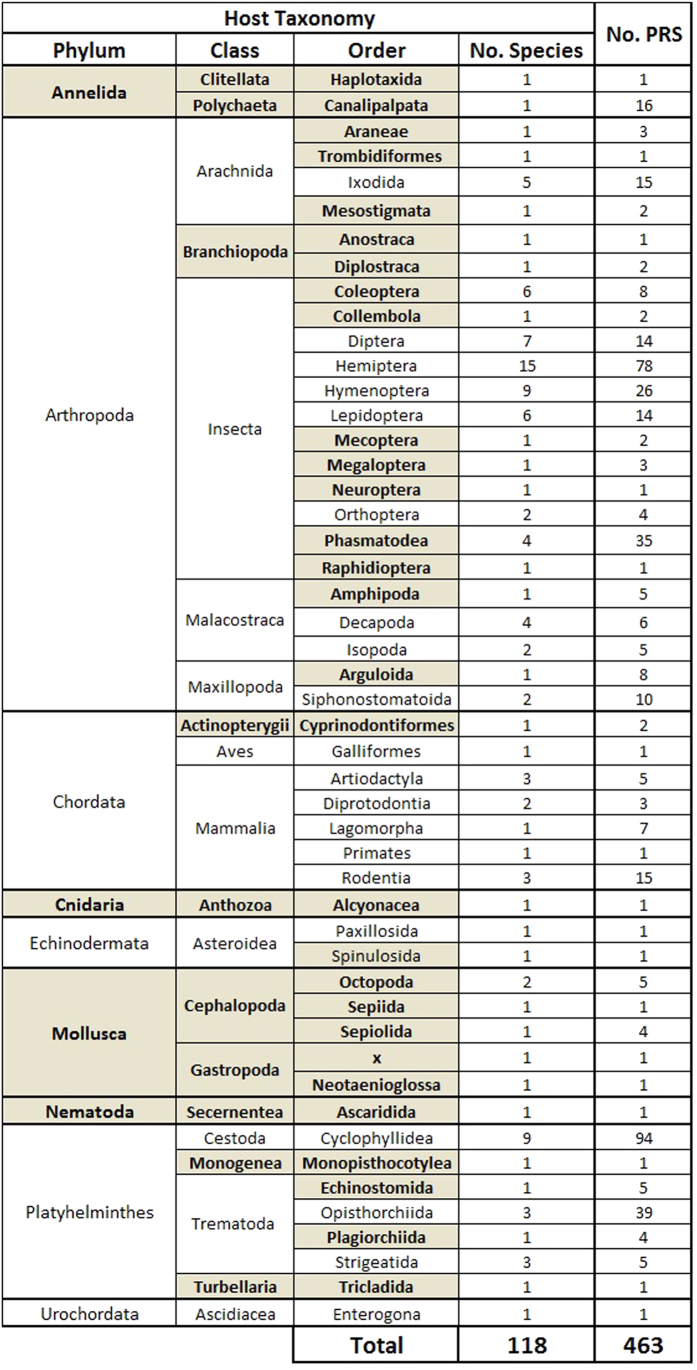
Summary of the distribution of PRSs among animal transcriptomes and genomes. Animal orders wherein PRSs were first identified in this study are represented in bold.

Altogether, we concluded that, while parvoviruses are probably associated with a wider variety of animals than has previously been thought, the PRSs we found were mostly associated with invertebrate species within phyla, classes, orders and families containing no previously known parvovirus host species (Fig. 2).

### PRSs likely originate from both fossil viral sequences and extant viruses

The PRSs that we detected could have had a number of different origins including: (1) endogenous “fossil” viral sequences resulting from ancient integration events; (2) endogenous viruses constituting latent infections resulting from recent integration events; or (3) exogenous viruses. Further, it was possible that all three types of PRS elements could have been present either within the cells of the species from which the transcriptome datasets were derived, or from (likely eukaryotic) species either parasitizing, or in some other way associated with, the species from which the transcriptome datasets were derived.

**Figure 3:**
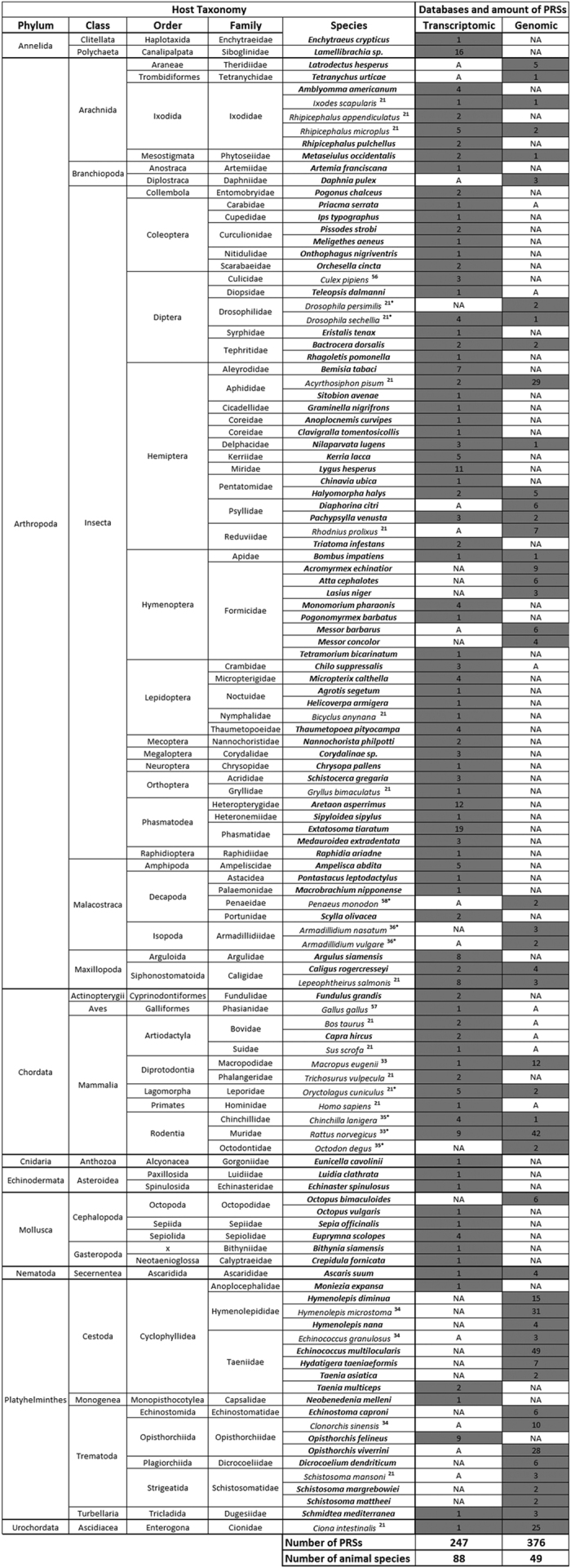
Distribution of PRSs in animal transcriptomes and genomes (in grey). Animal species wherein PRSs were first identified in this study are represented in bold while numbers correspond to references where PRSs were previously described. (*) PRS endogenization proved by PCRs. A: transcriptomic/genomic data (EST and TSA databases) available at NCBI. NA: Not Available, i.e. transcriptomic/genomic data (gss, WGS, chromosome and refseq_genomic databases) are not available at NCBI.

To identify PRSs potentially corresponding to endogenized parvoviral fragments, we used each of the 463 PRSs as queries to screen the publically available eukaryotic genome datasets (both assembled and unassembled) within the NCBI genomic database. This search identified 76 genomic sequences (summarized in Supplementary Table S3) of various sizes (0.05–8 kb) displaying significant matches (cutoff 95% identity, e-value <10^−5^) to PRSs within the genomes of 31 invertebrates from six phyla including 16 arthropods (33 PRSs), 13 platyhelminthes (36 PRSs), one mollusc (2 PRSs) and one nematode (4 PRS), for which endogenization of parvoviruses has never been found before (Table S3). In addition to the possibly endogenized PRSs identified in these 31 invertebrate species, PRSs were also identified in the genomes of 16 animal species for which potential parvovirus endogenization has been previously reported (including nine arthropods, six chordates and one platyhelminthes; Fig. 3)^20^,^21^,^31^,^32^,^33^,^34^.

Ancient integration events are often characterised by degraded integrons with the extent of degradation varying depending on whether the integrated sequences were selectively disadvantageous, beneficial or neutral^2^. We thus searched animal genome sequence databases for degraded PRSs including those containing potential transposable elements (TE) or repeated sequence insertions within the vicinity of the integration site, which may have contributed to their integration. We found 31 PRSs displaying truncations due to the accumulation of internal stop codons and/or adjacent transposable elements in the genomes of fifteen arthropod and seven platyhelminthes species (Table S3): a finding strongly supporting the hypothesis that these PRSs were the product of ancient endogenisation events in these phyla. In arthropods, whereas endogenization has been proposed previously for one of these PRSs–found within the genome of *A. pisum*^21^,^25^–we detected various other putative PRS endogenization events in a number of other Arthropods, i.e. six in Hymenoptera (*Formicidae*), three in Hemiptera (*Pachypsylla venusta*; family *Psyllidae*, *Halyomorpha halys*; family *Pentatomida*e, *Nilaparvata lugens*; family *Delphacidae*), one in Araneae (*Latrodectus hesperus*; family *Theridiidae*), one in Coleoptera (*Priacma serrata*; family *Cupedidae*) one in Mesostigmata (*Metaseiulus occidentalis*; family *Phytoseiidae*), one in Diplostraca (*Daphnia pulex*; family *Daphniidae*) and one in a Siphonostomatoida (*Caligus rogercresseyi*; family *Caligidae*), (Supplementary Tables S2 and S3); all these PRSs displayed internal stop codons and/or adjacent TE elements.

In platyhelminthes, potential densovirus endogenization has already been reported in one cestode (*Echinococcus granulosis*) and one trematode (*Schistosoma mansoni*), and all platyhelminthes associated PRSs recovered here share >95% nt identity with known platyhelminthes genome sequences^18^. The PRSs were detected in the genomes of 15 species (Fig. 3); mostly cestodes (e.g. *Taenia* sp. and *Echinococcus* sp.) and trematodes (e.g. *Schistosoma* sp.). All the PRSs detected in this phylum displayed ≥30% aa identity corresponding to the same domain of the NS1 protein although the inferred encoded amino acid sequences of this domain contained internal stop codons. For example, 39 PRSs were detected in the genome of the cestode, *Echinococcus multilocularis* that, when translated, displayed approximately 30% aa similarity with the NS1 of the shrimp parvovirus, Decapod penstyldensovirus 1 (PstDV1). These results suggest the endogenization of the NS1-like domain in the genome of several platyhelminthes species. The phylogenetic relationship between these PRSs will be addressed below.

Most genomic PRSs that we detected were, however, located at the extremities of genomic scaffolds that were less than 10 kb in length, and there was therefore limited information regarding their possible genomic context and flanking sequences: a factor which impaired our ability to definitively determine whether these sequences too were ancient degraded integrons associated with transposable elements or repeat-sequence insertions (Supplementary Table S3).

**Figure 4:**
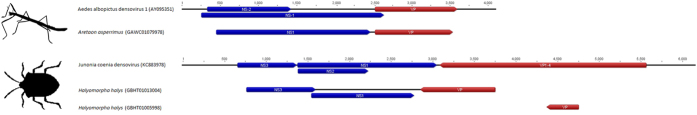
Organization of open reading frames of three PRSs (GAWC01079978 from *Aretaon asperrimus*, GBHT01013004 and GBHT01005998 from *Halyomorpha halys*). Arrowhead boxes indicate viral and predicted viral genes (NS are in blue and VP in red).

We next searched for PRSs corresponding to non-degraded viral ORFs as these might correspond to extant viruses. Four large PRSs covering both the NS and VP ORFs were found in both the genomes and transcriptomes of four arthropods (mentioned as NS-VP in Supplementary Table S2). One of these large PRS corresponded with the genomic sequence integrated in the pea aphid genome (*A. pisum*) that has been previously characterized by Liu *et al*.^35^. This PRS shares 52% aa identity with both NS and VP of the ambidensovirus infecting the aphid *Dysaphis plantaginea* (DpDV). A similar PRS (sharing >95% identity) was also detected in the genome of the peach-potato aphid (*Myzus persicae*)^23^. The transcription of both the NS and VP encoding genes was also demonstrated by these authors, suggesting that aphid endogenous parvoviral sequences represent recently integrated persistent viruses that could potentially become exogenous^25^. Remarkably, two other large PRSs (4.2 kb and 5.6 kb covering almost complete viral ORFs) were respectively recovered from the stick insect *Aretaon asperrimus*, belonging to the order *Phasmatodea*, and from the stink-bug, *Halyomorpha halys*, belonging to the order *Hemiptera*: neither species had previously been identified as densovirus hosts. These two large PRSs encompass two ORFs (NS1 and VP; GAWC1079978 recovered from *A. asperrimus*) and three ORFs (NS1, NS3 and VP; GBHT01013004 from *H. halys*; Fig. 4). The arrangement of these ORFs is similar to Brevi- and Ambidensoviruses that respectively infect mosquitoes and caterpillars (Fig. 4). Since only a few reads of the genomes of these potentially new insect hosts are available (for example we only found one contig containing the stink-bug PRS), it is not possible to conclude whether these PRSs are involved in latent infections by undescribed viruses (as is possibly the case for the integrated densovirus in aphids that is described above), or are derived from previously unknown extant exogenous *A*. *asperrimus* and/or *H. halys* infecting densoviruses^18^.

Last, we must emphasize that contamination of genomic datasets may come either from animals that are infected by parvoviruses or from animals that are infected by parasites that are themselves infected by parvoviruses. Intriguingly, we found one ~0.8 kb PRS in the genome sequence dataset of *Gregarina niphandrodes*; a protozoan Apicomplexa that infects a number of invertebrates and which was isolated from the coleopteran, *Tenebrio molitor* according to Genebank data. This PRS shared 100% identity with the VP sequence of a yet undescribed *Blatella germanica* densovirus-like virus found in the metagenome of insectivorous bat faeces^36^ (Table S2). We could not find any detectably homologous sequence within either the genome or the transcriptome of its potential host, *T. molitor*. Although we cannot rule out the possibility that the densovirus from which this PRS was derived was able to infect *G. niphandrodes*, we concluded that the PRS probably originated from a contaminant.

In total we discarded 16 PRSs from further analysis due to contamination concerns. Among these were three found in the transcript datasets of plant species (Table S2): all of these plant PRSs had high degrees of identity with extant insect-infecting densoviruses and were therefore likely derived from insects associated with the plants (Table S2). Similarly, PRSs found in the transcriptomes of three vertebrates (all amphibian species), one echinoderm (*Amphiura filiformis*) and one insect (*Drosophila ananassae*) were >85% identical to known mammal-infecting parvoviruses and were thus assumed to be contaminants.

Altogether these results highlight the large diversity of PRSs that can be found by screening publically available transcriptomes and genomes. In total, 623 PRSs were found when adding up all PRSs found in transcriptomic (247) and genomic databases (376) including the newly found and already reported sequences (Fig. 3). Our search identified that parvoviruses are/were associated with a large number of animal species that have never previously been identified as parvovirus host species (Summarized in Fig. 2).

### Phylogenetic analyses of PRSs

We next attempted to evaluate the genetic relationships of the PRSs with known exogenous parvoviruses. While the parvovirus protein NS1 contains a SF3 helicase domain that is highly conserved in all known parvoviruses (it can also be found in proteins of viruses in other families)^37^, the most conserved domain of parvoviral VPs, PLA2, is missing in certain parvoviruses: a factor which may explain why our search identified more sequences related to NS1 than to VP. The SF3 domain is thus typically used for phylogenetic analyses of divergent parvoviruses^18^.

As is shown in the *Parvoviridae* maximum likelihood trees (Figs 2 and 3), all exogenous known parvovirus species (in black text) were clearly placed within the 13 genera recognized by ICTV with bootstrap values >80%. While validating the use of the small SF3 domain of NS1 to study relationships amongst PRSs, it is apparent from the trees that most of the PRSs are situated on long branches that connect to the tree with low degrees of bootstrap support (<70%), basal to clusters of sequences from the established parvovirus genera. This phylogenetic placement is consistent with PRSs belonging to currently undescribed genus-level parvovirus lineages; although we cannot exclude ambiguous alignment of divergent sequences as the cause of low degrees of bootstrap support for the clustering of these PRSs with viruses from the known parvovirus genera.

**Figure 5:**
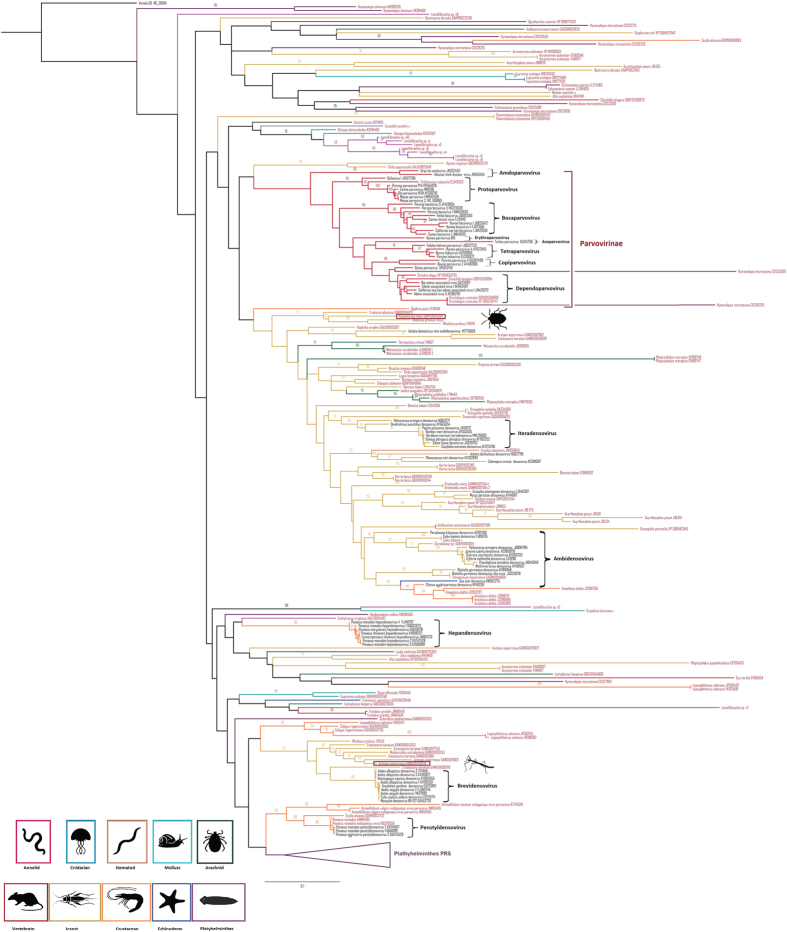
Maximum likelihood phylogenetic tree based on partial SF3 domains of the NS1 protein, including 74 parvovirus species (in black) and 264 PRSs (in red). The alignment was produced using MUSCLE 3.7 with default settings. The tree was rooted with the SF3 domain of the variola virus D5 protein. Bootstrap values >25% are indicated at each node. Scale bars correspond to amino acid substitutions per site. Genera of the *Parvoviridae* family are indicated in brackets. Associated hosts are indicated in the tree by different branch colours and silhouettes at the bottom of the figure.

Nevertheless, the PRSs drawn from the transcriptome datasets of animals belonging to particular genera were frequently monophyletic within clades supported by bootstrap values >50%: consistent with the hypothesis that these PRSs are derived from viruses belonging to undiscovered parvovirus lineages with genus-specific host-ranges. This situation is exemplified with PRSs found within transcriptome datasets of ticks, stick insects and stink-bugs (Fig. 5). Interestingly, the two large transcripts that correspond to almost complete densovirus genomes that were detected in the stink-bug and stick insect transcriptome datasets cluster together with PRSs found in related triatomine insects in either the *Reduviidae* family (for the stink bug PRSs) or the *Phasmatodea* order (for the stick insect PRSs). These large PRSs might correspond to exogenous or persistent viruses belonging to densovirus lineages that infect these insects (Fig. 4). Interestingly, PRSs found in four classes of marine molluscs and more related to vertebrate parvoviruses according to results above, branched out of all known parvovirus genera (represented by the light blue branches in Fig. 5), although we cannot exclude some contamination of these animals. These results suggest that these PRSs belong to new parvovirus lineages yet to be characterized in this phylum.

**Figure 6:**
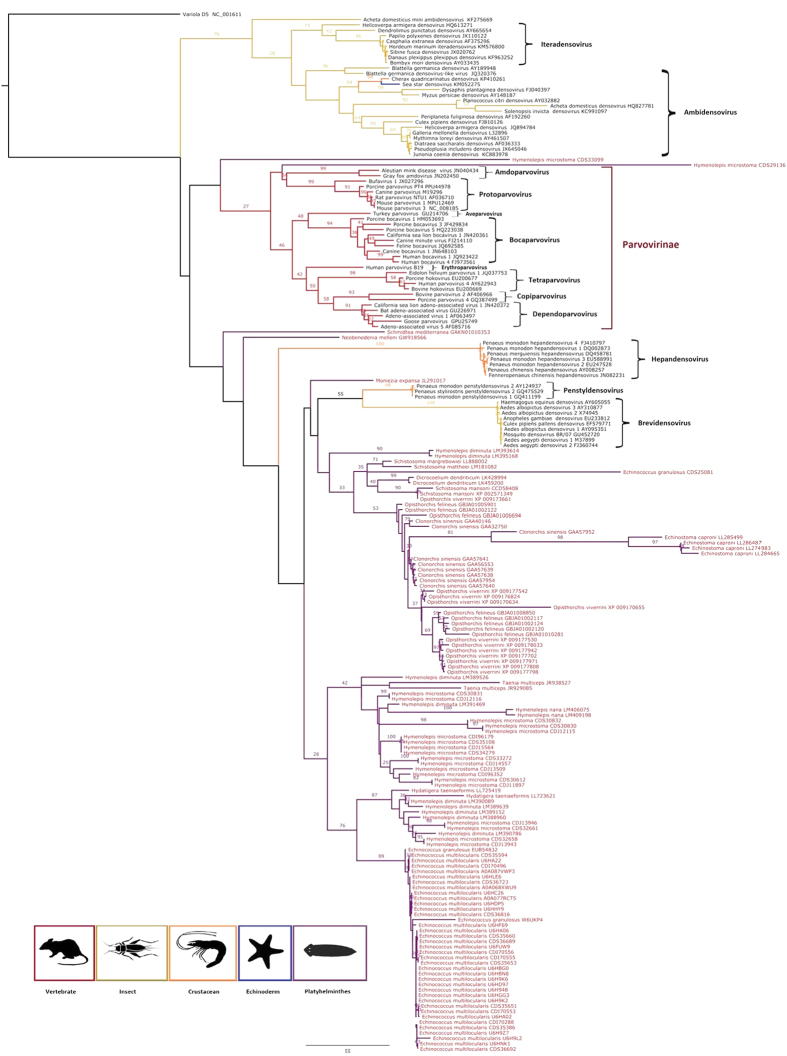
Maximum likelihood phylogenetic tree of platyhelminthes PRSs based on partial SF3 domains of the NS1 protein, including 74 parvovirus species (in black) and 118 platyhelminthes PRSs (in red). The alignment was produced using MUSCLE 3.7 with default settings. The tree was rooted with the variola virus D5 protein SF3 domain. Bootstrap values >25% are indicated at each node. Scale bars correspond to amino acid substitutions per site. The genera of the *Parvoviridae* family are indicated in brackets. Host phyla are represented by different colours and silhouettes at the bottom of the figure.

Such clustering of PRSs according to the evolutionary relationships of the animal species they are associated with is particularly apparent for PRSs found within the platyhelminthes transcriptome datasets (Fig. 6). PRSs found within these datasets form two clades supported by >70% bootstrap values (Fig. 6). The inferred SF3 amino acid sequences encoded by these PRSs share identities of <30% with those of previously known parvoviruses, suggesting that these flatworm-associated PRSs may represent new genera (Fig. 6) of either extant but undiscovered circulating parvoviruses, or integrated (and possibly extinct) parvoviruses.

The data presented here indicate that parvoviruses are likely widespread among multicellular animals. If we consider all clusters with bootstrap values >70%, then we can tentatively estimate that these phylogenetic analyses indicate the existence of around 20 currently undescribed genus-level densovirinae lineages.

## Discussion

Since the discovery of parvoviruses four decades ago, our perception of the species diversity within this family has been strongly biased by an overwhelming focus on discovering viruses of health and economic interest. While a high diversity of viral genome organisations and sequences has been revealed, few genomes were characterized: a factor which has limited our understanding of the global diversity, prevalence and host-associations of these viruses. The advent of the genomic era has now provided an alternative way to explore virus diversity in a slightly less biased way via the computational screening of large transcriptome and whole genome sequence datasets^18^,^21^,^22^.

By scanning public genomic and transcriptomic resources, we have found that parvoviruses likely infect a larger number and wider diversity of animal-hosts, particularly among invertebrate phyla, than has previously been appreciated. These newly discovered potential parvovirus hosts include molluscs, annelids, nematodes, cnidarians and arthropods.

Although these results suggest widespread parvovirus integration into the genomes of diverse invertebrates, the limited number of complete invertebrate genome sequences that are currently available hinders both the definitive identification of these PRSs as integrated sequences, and an accurate estimation of the time-scale of individual PRS integration events. Data summarized in Fig. 3 and Supplementary Table S2 highlight the fact that 60% (376/623) of PRSs were found in animal genomes, among which 10% (37/367) are likely integrated. Integration was thus uncertain for 90% of the PRSs, mostly due to either unavailable or incomplete genomes for most of the invertebrate phyla for which transcriptome data was available. However, it is plausible that some of the endogenous PRSs were integrated millions of years ago since the presence of what appear to be closely related PRSs in different mammalian species have previously suggested that parvoviruses have likely coexisted with mammals for at least 98 million years^21^.

It is particularly clear that most of the PRSs identified in this study were more similar to members of the *Densovirinae* sub-family than they were to members of the *Parvovirinae* sub-family. Considering that arthropod species vastly outnumber all other animal species, such over-representation of densoviruses, although unsurprising, contrasts with the similar numbers of described species in both subfamilies in the current ICTV report^18^,^19^.

We cannot exclude the possibility that contamination of transcriptome and genome sequence datasets with unaccounted for eukaryote and/or viral DNA could yield spurious host-virus associations in studies such as that carried out here: as is apparently exemplified by the presence of PRSs in a small number of plant datasets. It is noteworthy, however, that densovirus-plant associations actually do occur in nature. For example, aphid-infecting densoviruses can be injected into, and circulate within, plants^38^. It has, in fact, been speculated that plants might be stationary vectors of some densoviruses that infect plant-feeding insects^38^.

It has been proposed that all viruses in a parvovirus genus should be monophyletic and encode NS1 proteins that share >30% amino acid sequence identity to each other^18^. The phylogeny that we have produced for the SF3 helicase domain of NS1 indicated that 15% of the PRSs that contain this domain are highly divergent. The low degrees of similarity shared between these PRSs and both known parvoviruses and the other PRSs meant that they could not be reliably aligned: a factor that could have contributed to these divergent sequences falling on long isolated branches of the phylogenetic tree. While the intermingling of these divergent PRS lineages amongst known members of the *Parvovirinae* and *Densovirinae* genera, suggests that numerous genus-level parvovirus lineages are presently undescribed, we can also not exclude the possibility that some of these PRSs may be derived from virus families, such as *Bidnaviridae*, that are related to the parvoviruses. Like parvoviruses, the bidnaviruses are also ssDNA viruses that express a SF3 domain containing protein^18^,^39^. Unlike parvoviruses, however, they have two-component genomes and encode DNA polymerases. Although all of the PRSs discovered here were more closely related to known parvovirus sequences than to known bidnaviruses, the possibility remains that some of these PRSs are potentially derived from viruses belonging to currently undescribed families.

Here we have highlighted the extraordinary diversity of PRSs that can be found in public databases. As more animal sequences will be released we can anticipate that our knowledge of the diversity of parvoviruses will also keep improving. Combining such database searches with more directed viral metagenomics approaches and classical etiological survey, will be of great value both for discovering new parvoviruses and, as more endogenous PRSs are discovered within eukaryote genomes, for illuminating the deep evolutionary history of this family in relation to that of the host species that they infect.

## Methods

### Biological samples and transcriptome datasets

*Lamellibrachia* sp. (marine polychaete annelid) samples were collected in 2007 in the Gulf of Mexico at 1250 m depth for individual, GA27M, and in the Gulf of Guinea at 580–670 m depth for individuals, GA27P, GA27S and GA27U. Vestimentum tissue was dissected and stored immediately in liquid nitrogen. Due to poor yield with standard total RNA isolation methods, we used a modified protocol based on a Trizol-Chloroform method combined with a QIAshredder column (Qiagen) purification step^40^ involving the addition of 4 μl of glycogen (Ambion, final concentration = 0.04 mg/μL) to increase RNA yield and a further polyvinylpolypyrrolidone (PVPP) purification step. Five μg of total RNA was reverse-transcribed using the SMART cDNA library construction kit (Clontech, Mountain View, USA). Libraries were sequenced to produce 100 bp paired-end reads on a Genome Analyzer II or Hiseq 2000 (Illumina, Inc.). Low-quality read extremities were trimmed using the SeqClean program (http://compbio.dfci.harvard.edu/tgi/). Reads were deposited in the Sequence Read Archive (SRA) NCBI database under bioproject PRJNA302863, accession numbers SRX1440230, SRX1447229, SRX1447303 and SRX1447300. *Lamellibrachia* transcriptomes produced in this study, as well as individual previously published transcriptomes of *Artemia franciscana* (individual GA17B; SRX565006), *Crepidula fornicata* (individual GA22E; SRX565072), *Eunicella cavolinii* (individual GA31L; SRX565138) and *Messor barbarus* (individual GA40E; SRX565206) were successively assembled using ABYSS V 1.2.0^41^ and CAP3^42^. This assembly method was previously found to be suitable for other mollusc and animal transcriptomes^43^,^44^. Three supplementary transcriptomes of whole individual adults of *Messor barbarus*, *Messor concolor* and *Culex pipiens* were assembled as above and were added to this dataset (N. Galtier, unpublished data). In total eleven transcriptomes have been used in this study, four of which were generated for this study (*Lamellibrachia*) and seven of which were previously used for animal genomics studies that did not involve virus detection (*Artemia franciscana*, *Culex pipiens*, *Crepidula fornicata*, *Eunicella cavolinii*, two *Messor barbarus* and *Messor concolor*).

### Homology searches for parvovirus-related sequences (PRSs)

We assembled a dataset of NS and VP amino acid sequences derived from each of the 74 parvovirus species, recognized and yet to be approved by the ICTV (all obtained from GenBank; genomes listed in Table S1). These sequences were used as queries to perform BLASTX searches for PRSs within all the non-redundant (nr) nucleotide and protein sequences at the NCBI, including the cDNA EST (http://www.ncbi.nlm.nih.gov/nucest/), TSA (http://www.ncbi.nlm.nih.gov/genbank/tsa), and Uniprot databases^29^. All sequences from these databases that matched parvovirus sequences (E-value < 10^−3^) were selected and used as queries to perform BLASTX or BLASTP reciprocal searches of the cDNA EST, TSA and Uniprot databases. Sequences were considered PRSs when they matched known parvovirus sequences with associated BLASTX or BLASTP E-values < 10^−3^. The eleven new transcriptomes were also screened for the presence of PRSs as described above. Complete and 5′- or 3′-truncated ORFs were detected using Prodigal V2_60^45^,^46^ using the standard genetic code. ORFs displaying internal stretches of undetermined nucleotides (N) were also considered. Putative protein sequences were first annotated by detecting protein homology using the HHblits component of the HHSuite package^47^,^48^ of nr protein sequences of the NCBI database. ORFs matching parvoviral proteins (E-values < 10^−3^) were selected and used as query for reciprocal BLASTP searches of the cDNA EST, TSA and Uniprot databases as described above.

### Detection of endogenous parvovirus-related sequences

The 463 PRSs found from BLASTX searches described above were used as queries against the reference genomic sequences (Refseq_genomic, http://www.ncbi.nlm.nih.gov/refseq/), chromosome (http://www.ncbi.nlm.nih.gov/genome/), GSS (Genomic survey sequences) (http://www.ncbi.nlm.nih.gov/nucgss/) and WGS (Whole-Genome Shotgun contigs) (http://www.ncbi.nlm.nih.gov/genbank/wgs) databases using BLASTN and tBLASTN, with a minimum percentage similarity cutoff of 95% and an E-value cutoff of 10^−5^. Five hundred nucleotide long genomic fragments located up- and down-stream of each PRS were scanned for transposable elements (TE) or repetitive sequences using WSCensor (http://www.girinst.org/censor/).

### Phylogenetic analyses

The putative amino acid sequences of the SF3 helicase domains of parvoviral NS1and PRS NS1-like proteins were used for phylogenetic analyses. All PRSs were translated *in silico* using ORF finder (cut off >300 bp) (http://www.ncbi.nlm.nih.gov/projects/gorf/). Putative domains of the resulting proteins were predicted using Interproscan5 49. Among the 463 PRSs, 264 contained a SF3 helicase domain sequence from which 191 aa-long fragments were aligned with the corresponding SF3 fragments from the 74 known parvoviruses (Table S1) using MUSCLE 3.7 (16 iterations) with default settings^50^. Aligned sequences were manually edited (full alignment of the 191 aa-long partial SF3 helicase domains, is provided in FASTA format, and is presented in Supplementary Figure S1). Maximum likelihood phylogenetic trees were produced from this alignment using PhyML 3.1 51 with a Blossum + G + F + I amino acid substitution model chosen as the best-fit using ProtTest^52^. Five hundred bootstrap replicates were used to test the support of branches. Trees were visualized with FigTree 1.4 (http://tree.bio.ed.ac.uk/software/figtree/). In addition, a second tree focusing on the evolutionary relationships of 118 PRSs derived from platyhelminthes together with the 74 representative parvovirus SF3 domain sequences (Table S1) was constructed using the same approaches described above (full alignment of the 156 aa-long partial SF3 helicase domains is provided in FASTA format and in Supplementary Figure S2). The variola D5 protein (Genbank accessionnumber: P33069) was used in both cases as an outgroup to root the trees^53^,^54^,^55^,^56^,^57^,^58^.

## Additional Information

**How to cite this article**: François, S. *et al*. Discovery of parvovirus-related sequences in an unexpected broad range of animals. *Sci. Rep*. **6**, 30880; doi: 10.1038/srep30880 (2016).

## Supplementary Material

Supplementary Information
