# Supplementary material for: Discovery of parvovirus-related sequences in an unexpected broad range of animals
Source: Sci Rep. 2016 Sep 7;6:30880. doi: 10.1038/srep30880 (PMC5013282; doi:10.1038/srep30880)
Supplement: Supplementary Information [file srep30880-s1.pdf]

Discovery of parvovirus-related sequences in an unexpected broad range of animals

S. François[1], D. Filloux[2], P. Roumagnac[2], D. Bigot[3], P. Gayral[3, 4], D. P. Martin[5], R. Froissart[2,6] & M. Ogliastro[1*]

Supplementary Figures and Tables

SUPPLEMENTARY DATA

Table S1. Reference parvoviral genomes used to search for PRSs and for phylogenetic analyses.

Table S2. Detailed information about PRSs found in both genomic and transcriptomic databases. Host taxonomy and BLAST results are given for each PRS. Animal species containing PRSs displaying < 30% identity with extant parvoviruses are highlighted in blue. Highlighted in yellow are animal species containing PRSs displaying > 85% identity with extant parvoviruses.

Table S3. Detailed information about PRSs that display homologies with genomic databases only (Refseq_genomic, chromosome, gss and WGS).

Figure S1: Amino acid alignment of the SF3 helicase domain of 264 PRSs and 74 parvoviruses. The variola D5 protein SF3 domain was used as outgroup. The alignment was produced using MUSCLE 3.7 with default settings. Aligned sequences were trimmed and columns with gaps were manually removed.

Figure S2: Amino acid alignment of the SF3 helicase domain of 118 platyhelminthes PRSs and 74 parvoviruses. The variola D5 protein SF3 domain was used as an out-group. The alignment was produced using MUSCLE 3.7 with default settings. Aligned sequences were trimmed and columns in the alignment with gaps were manually removed.

Phylum	Class	Order	Family	Organism	Accession No. (PRS)	Genomic Database	Position (L)	Position (R)	Length (bp)	%Coverage	E value	% Identity	Accession No.	Size (bp)	Adjacent transposable element	Degenerated mutations relative to viral genes
Arthropoda		Araneae	Theridiidae	**Latrodectus hesperus**	GBCS01037246	WGS	553	346	207	90%	9,00E-102	100%	JJRX01010780.1	2545	Yes	internal stop codons
					GBCS01014800		3499	3610	111	3%	2,00E-41	95%	JJRX01010779.1	3611	Yes	
		Mesostigmata	Phytoseiidae	**Metaseiulus occidentalis**	JI999665	genome (reference only)	2596	3387	791	100%	0.0	99%	NW_003803976.1	536467	Yes	internal stop codons
	Branchiopoda	Diplostraca	Daphniidae	**Daphnia pulex**	EFX61411	WGS	2045	3772	1727	100%	0.0	99%	ACJG01014942.1	6319	No	internal stop codons
	Insecta	Coleoptera	Cupedidae	**Priacma serrata**	GACO01003330	WGS	564	491	73	9%	6,00E-26	95%	AGRH01000640.1	8293	No	internal stop codons
		Diptera	Tephritidae	**Bactrocera dorsalis**	GAKP01023238	WGS	453219	452802	417	55%	0.0	99%	JFBF01000220.1	459848	No	
							408	378	30	4%	2,00E-06	100%	JFBF01000093.1	1167390	No	
		Hemiptera	Delphacidae	**Nilaparvata lugens**	HS466630	WGS	1335	1502	167	100%	4,00E-81	100%	AOSB01120472.1	2333	No	
					GANM01012737		1548	824	724	100%	0.0	99%	AOSB01120472.1	2333	No	internal stop codons
			Pentatomidae	**Halyomorpha halys**	GBHT01013004	WGS	12819	15765	2946	98%	0.0	98%	JMPT01017517.1	16063	No	
							440	3343	2903	98%	0.0	95%	JMPT01038455.1	18195	Yes	
							41367	43129	1762	60%	0.0	96%	JMPT01084032.1	44282	Yes	
							7323	6481	842	29%	0.0	98%	JMPT01052785.1	7323	No	
							1809	956	853	30%	0.0	97%	JMPT01084370.1	3319	Yes	
			Psyllidae	**Diaphorina citri**	XM_008484718	WGS	3021	3732	711	100%	0.0	100%	AWGM01156254.1	11892	No	
							1	81	80	9%	2,00E-33	100%	AWGM01025324.1	226	No	
							878	817	61	10%	8,00E-19	95%	AWGM01000952.1	1117	No	
							291	243	48	8%	2,00E-14	98%	AWGM01064873.1	293	No	
				**Pachypsylla venusta**	GAOP01032067	WGS	6232	6859	627	100%	0.0	99%	AZLD01217521.1	7421	Yes	internal stop codons
					GAOP01141099		3944	3729	215	100%	6,00E-100	98%	AZLD01011675.1	4115	No	
		Hymenoptera	Apidae	**Bombus impatiens**	JI097654	WGS	1329	1660	331	99%	2,00E-163	98%	AEQM02016195.1	3848	No	internal stop codons
			Formicidae	**Acromyrmex echinatior**	XM_011070319.1	WGS	27037	25815	1222	100%	0.0	100%	AEVX01004976.1	79951	No	
					EGI68940		1482	1790	308	19%	4,00E-56	96%	AEVX01005448.1	2528	Yes	
							3686	3889	203	8%	9,00E-36	99%	AEVX01005453.1	5028	Yes	
				**Atta cephalotes**	XM_012209122.1	WGS	20667	21660	993	100%	0.0	96%	ADTU01008063.1	23996	No	internal stop codons
					W4VWX9		112	699	587	47%	6,00E-127	100%	ADTU01004457.1	5143	Yes	
				**Lasius niger**	KMQ81887.1	WGS	604	2	602	100%	5,00E-124	97%	LBMM01030606.1	776	No	
				**Monomorium pharaonis**	XM_012666055.1	WGS	20535	17977	2558	100%	0.0	98%	BBSX02014388.1	21843	No	
							1	92	91	3%	3,00E-39	100%	BBSX02013400.1	2878	No	
					XM_012666053.1		1	92	91	3%	3,00E-39	100%	BBSX02013400.1	2878	No	
							20535	17965	2570	100%	0.0	98%	BBSX02014388.1	21843	No	
							1	92	91	3%	3,00E-39	100%	BBSX02013400.1	2878	No	
				**Pogonomyrmex barbatus**	XM_011645096.1	WGS	4152	6241	2089	100%	0.0	99%	ADIH01015354.1	12755	Yes	
					XM_011645095.1		4152	6238	2086	100%	0.0	100%	ADIH01015354.1	12755	Yes	
	Maxillopoda	Siphonostomatoida	Caligidae	**Caligus rogercresseyi**	GAZX01015685	WGS	71	2189	2118	78%	0.0	99%	LBBV01106530.1	2515	Yes	internal stop codons
							1863	329	1534	57%	0.0	99%	LBBU01242504.1	1863	No	
					GAZX01042735		476	963	487	100%	0.0	100%	LBBV01271803.1	994	No	
							519	32	487	100%	0.0	100%	LBBU01149111.1	2470	Yes	
Mollusca	Cephalopoda	Octopoda	Octopodidae	**Octopus bimaculoides**	KOF81493.1	WGS	5332	5847	515	100%	6,00E-103	93%	LGKD01669633.1	7219	Yes	
					KOF62817.1		935	1672	737	100%	3,00E-174	100%	LGKD01331296.1	2875	Yes	
Nematoda	Secernentea	Ascaridida	Ascarididae	**Ascaris suum**	JI174103	WGS	14090	14131	41	3%	6,00E-12	100%	ANBK01004617.1	39446	Yes	
							4254	4213	41	3%	6,00E-12	100%	AMPH01016759.1	26950	Yes	
							25879	25838	41	3%	6,00E-12	100%	AEUI02001051.1	53597	Yes	
							36	1	35	2%	5,00E-07	97%	AEUI02016456.1	219	No	
latyhelminthe	Cestoda	Cyclophyllidea	Hymenolepididae	**Hymenolepis diminua**	LM390089.1	Genbank nr	1032	2378	1346	100%	0.0	100%	LM390089.1	2383	No	
				**Hymenolepis microstoma**	CDS32658	WGS	74578	73628	950	93%	0.0	100%	CBLW020003802.1	75037	No	
				**Hymenolepis nana**	LM406075.1	Genbank nr	1	4839	4838	100%	0.0	100%	LM406075.1	4839	No	
							35	2518	2483	100%	0.0	96%	LM409198.1	2518	No	
			Taeniidae	**Echinococcus multilocularis**	U6HGG3	WGS	5004	6296	1292	100%	0.0	99%	CBLO020000155.1	7682	No	
							3092	4384	1292	100%	0.0	99%	CBLO020000266.1	61081	No	
							890	2182	1292	100%	0.0	97%	CBLO020000868.1	2622	No	
							1842	604	1238	100%	0.0	99%	CBLO020000226.1	4432	No	
							10653	9478	1175	99%	0.0	97%	CBLO020000544.1	33361	No	
							346	1089	743	57%	2,00E-163	99%	CBLO020000490.1	10554	No	
							2422	1733	689	53%	2,00E-147	95%	CBLO020000966.1	5011	No	
							975	412	563	43%	1,00E-119	96%	CBLO020000024.1	3149	No	
							3178	2615	563	43%	4,00E-118	97%	CBLO020000171.1	4825	No	
							3491	3934	446	34%	3,00E-90	97%	CBLO020000092.1	4638	No	
				**Hydatigera taeniaeformis**	LL723621.1	Genbank nr	1	3111	3110	100%	0.0	100%	LL723621.1	3111	No	internal stop codons
							241	507	266	15%	2,00E-118	97%	LL748207.1	507	No	
							579	724	145	7%	3,00E-64	99%	LL740453.1	724	No	
							1391	1465	74	2%	7,00E-22	95%	LL728283.1	1751	No	
							72	114	42	1%	3,00E-08	98%	LL727136.1	1978	No	
				**Taenia asiatica**	LM127006.1	Genbank nr	381	620	239	100%	9,00E-99	100%	LM127006.1	12071	No	internal stop codons
	Trematoda	Echinostomida	Echinostomatidae	**Echinostoma caproni**	LL286487.1	Genbank nr	1168	239	929	100%	0.0	100%	LL286487.1	1623	No	internal stop codons
		Opisthorchiida	Opisthorchiidae	**Clonorchis sinensis**	GAA57638	WGS	1847	1029	818	100%	9,00E-172	95%	BADR02003990.1	27389	No	
				**Opisthorchis viverrini**	XP_009176824	WGS	97	1590	1493	97%	0.0	97%	JACJ01026580.1	2354	No	
							3	596	593	45%	6,00E-137	95%	JACJ01037170.1	706	No	
							3	527	524	34%	1,00E-117	95%	JACJ01041207.1	527	No	
							1	474	473	30%	1,00E-109	96%	JACJ01043459.1	474	No	
							1	248	247	29%	1,00E-100	97%	JACJ01044501.1	452	No	
							4	246	242	15%	5,00E-50	100%	JACJ01060560.1	248	No	
							3	206	203	13%	2,00E-38	97%	JACJ01069043.1	206	No	
		Plagiorchiida	Dicrocoeliidae	**Dicrocoelium dendriticum**	LK446595.1	Chromosomes	1	1838	1837	100%	0.0	100%	LK446595.1	1838	No	internal stop codons
					LK428994.1		1	2134	2133	100%	0.0	100%	LK428994.1	2134	No	internal stop codons
		Strigeatida	Schistosomatidae	**Schistosoma margrebowiei**	LL888002.1	GenBank nr	1	8085	8084	100%	0.0	100%	LL888002.1	8085	Yes	internal stop codons
				**Schistosoma mattheei**	LM181082.1	GenBank nr	1	2250	2249	100%	1,00E-96	100%	LM181082.1	2250	No	internal stop codons
	Turbellaria	Tricladida	Dugesiidae	**Schmidtea mediterranea**	GAKN01010353	WGS	8883	9542	659	100%	0.0	100%	AUVC01112254.1	32292	No	
							16546	15886	660	100%	0.0	99%	ASXR01032274.1	18356	No	
							48324	48984	660	100%	0.0	99%	AAWT01002411.1	169447	No	

Host Taxonomy					Accession Number	Fragment Lenght	Database	BLAST							
Phylum	Class	Order	Family	Species			Genomic/trancriptomic	Virus species	Virus genus	Virus sub-family	Accession No	E-value	Identity (%)	Coverage (%)	Protein
Annelida	Clitellata	Haplotaxida	Enchytraeidae	Enchytraeus crypticus	GALF01015487	1121 (nt)	TSA	Culex pipiens densovirus	Ambidensovirus	Densovirinae	YP_002887625	1,00E-12	30%	43%	NS
Annelida	Polychaeta	Canalipalpata	Siboglinidae	Lamellibrachia sp.	x	465 (nt)	RNAseq	Papilio polyxenes densovirus	Iteradensovirus	Densovirinae	YP_006589928	4,00E-08	31%	75%	NS
					x	309 (nt)	RNAseq	Sibine fusca densovirus	Iteradensovirus	Densovirinae	YP_006576512	8,00E-05	30%	95%	NS
					x	495 (nt)	RNAseq	Helicoverpa armigera densovirus	Iteradensovirus	Densovirinae	AEI26261	2,00E-05	33%	46%	VP
					x	126 (nt)	RNAseq	Helicoverpa armigera densovirus	Iteradensovirus	Densovirinae	YP_004678721	2,00E-06	51%	97%	VP
					x	522 (nt)	RNAseq	Helicoverpa armigera densovirus	Iteradensovirus	Densovirinae	AEI26261	4,00E-07	36%	44%	VP
					x	654 (nt)	RNAseq	Helicoverpa armigera densovirus	Iteradensovirus	Densovirinae	AEI26261	2,00E-06	31%	35%	VP
					x	459 (nt)	RNAseq	Dendrolimus punctatus densovirus	Iteradensovirus	Densovirinae	YP_164339	1,00E-08	34%	58%	NS
					x	582 (nt)	RNAseq	Danaus plexippus plexippus iteravirus	Iteradensovirus	Densovirinae	YP_009021036	4,00E-06	32%	52%	NS
					x	930 (nt)	RNAseq	Bombyx mori densovirus 5	Iteradensovirus	Densovirinae	NP_694834	4,00E-13	30%	43%	NS
					x	513 (nt)	RNAseq	Sibine fusca densovirus	Iteradensovirus	Densovirinae	YP_006576512	3,00E-08	32%	64%	NS
					x	1695 (nt)	RNAseq	Papilio polyxenes densovirus	Iteradensovirus	Densovirinae	YP_006589928	2,00E-11	31%	28%	NS
					x	477 (nt)	RNAseq	Helicoverpa armigera densovirus	Iteradensovirus	Densovirinae	YP_004678721	5,00E-06	34%	47%	VP
					x	1035 (nt)	RNAseq	Dendrolimus punctatus densovirus	Iteradensovirus	Densovirinae	YP_164339	2,00E-06	30%	28%	NS
					x	1743 (nt)	RNAseq	Helicoverpa armigera densovirus	Iteradensovirus	Densovirinae	AEI26261	1,00E-06	28%	27%	VP
					x	498 (nt)	RNAseq	Sibine fusca densovirus	Iteradensovirus	Densovirinae	YP_006576512	4,00E-04	30%	59%	NS
					x	807 (nt)	RNAseq	Helicoverpa armigera densovirus	Iteradensovirus	Densovirinae	AEI26255	1,00E-15	30%	69%	NS
Arthropoda	Arachnida	Trombidiformes	Tetranychidae	Tetranychus urticae	T1KBZ7	100 (aa)	UNIPROT	Helicoverpa armigera densovirus	Iteradensovirus	Densovirinae	AEI26255	6,00E-12	37%	79%	NS
Arthropoda	Arachnida	Araneae	Theridiidae	Latrodectus hesperus	GBCS01020635	2578 (nt)	TSA	Turkey parvovirus TP1-2012/HUN	Aveparvovirus	Parvovirinae	AHF54687	2,00E-43	43%	40%	NS
					GBCS01037246	231 (nt)	TSA	Eidolon helvum parvovirus 2	Tetraparvovirus	Parvovirinae	AGL97807	2,00E-07	40%	88%	NS
					GBCS01014800	2861 (nt)	TSA	Turkey parvovirus TP1-2012/HUN	Aveparvovirus	Parvovirinae	AHF54687	1,00E-38	43%	37%	NS
Arthropoda	Arachnida	Ixodida	Ixodidae	Amblyomma americanum	JZ180183	557 (nt)	EST	Dendrolimus punctatus densovirus	Iteradensovirus	Densovirinae	YP_164339	2,00E-11	45%	44%	NS
					GAGD01002820	680 (nt)	TSA	Dendrolimus punctatus densovirus	Iteradensovirus	Densovirinae	YP_164339	3,00E-04	38%	30%	NS
					JZ170404	680 (nt)	EST	Dendrolimus punctatus densovirus	Iteradensovirus	Densovirinae	YP_164339	3,00E-04	38%	30%	NS
					GAGD01007016	557 (nt)	TSA	Dendrolimus punctatus densovirus	Iteradensovirus	Densovirinae	YP_164339	2,00E-11	45%	44%	NS
Arthropoda	Arachnida	Ixodida	Ixodidae	Ixodes scapularis	XP_002401871	582 (nt)	nr	Helicoverpa armigera densovirus	Iteradensovirus	Densovirinae	HQ613264	5,00E-51	46%	93%	NS
					EW910578	781 (nt)	EST	Dendrolimus punctatus densovirus	Iteradensovirus	Densovirinae	YP_164339	[illegible]	[illegible]	[illegible]	NS
Arthropoda	Arachnida	Ixodida	Ixodidae	Rhipicephalus appendiculatus	CD786958	699 (nt)	EST	Helicoverpa armigera densovirus	Iteradensovirus	Densovirinae	AEI26255	9,00E-48	42%	89%	NS
					CD793420	585 (nt)	EST	Helicoverpa armigera densovirus	Iteradensovirus	Densovirinae	AEI26255	2,00E-22	49%	82%	NS
Arthropoda	Arachnida	Ixodida	Ixodidae	Rhipicephalus microplus	CK189748	575 (nt)	EST	Dendrolimus punctatus densovirus	Iteradensovirus	Densovirinae	YP_164339	[illegible]	[illegible]	29%	NS
					CK189747	575 (nt)	EST	Dendrolimus punctatus densovirus	Iteradensovirus	Densovirinae	YP_164339	[illegible]	[illegible]	29%	NS
					EW679313	626 (nt)	EST	Helicoverpa armigera densovirus	Iteradensovirus	Densovirinae	AEI26255	[illegible]	[illegible]	68%	NS
					CK175050	865 (nt)	EST	Periplaneta fuliginosa densovirus	Iteradensovirus	Densovirinae	NP_051016	5,00E-07	22%	44%	VP
					CK175049	692 (nt)	EST	Periplaneta fuliginosa densovirus	Iteradensovirus	Densovirinae	NP_051016	2,00E-07	28%	44%	VP
Arthropoda	Arachnida	Ixodida	Ixodidae	Rhipicephalus pulchellus	GACK01007160	594 (nt)	TSA	Helicoverpa armigera densovirus	Iteradensovirus	Densovirinae	AEI26255	[illegible]	[illegible]	[illegible]	NS
					L7M443	197 (aa)	UNIPROT	Helicoverpa armigera densovirus	Iteradensovirus	Densovirinae	AEI26255	5,00E-52	47%	94%	NS
Arthropoda	Arachnida	Mesostigmata	Phytoseiidae	Metaseiulus occidentalis	JI999665	430 (nt)	TSA	Densovirus SC2121	Ambidensovirus	Densovirinae	AFH02759	1,00E-14	38%	90%	VP
					JL018010	1652 (nt)	TSA	Culex pipiens densovirus	Ambidensovirus	Densovirinae	YP_002887625	9,00E-36	29%	49%	NS
Arthropoda	Branchiopoda	Anostraca	Artemiidae	Artemia franciscana	x	1648 (nt)	RNAseq	Acheta domesticus mini ambidensovirus	Ambidensovirus	Densovirinae	YP_008658570	4,00E-13	37%	22%	VP
Arthropoda	Branchiopoda	Diplostraca	Daphniidae	Daphnia pulex	EFX61411	622 (aa)	nr	Culex pipiens densovirus	Ambidensovirus	Densovirinae	YP_002887625	2,00E-09	22%	69%	NS
					EFX65985	207 (aa)	nr	Acheta domesticus mini ambidensovirus	Ambidensovirus	Densovirinae	YP_008658570	7,00E-04	35%	39%	VP
Arthropoda	Insecta	Collembola	Entomobryidae	Orchesella cincta	GAMM01017564	1543 (nt)	TSA	Mythimna loreyi densovirus	Ambidensovirus	Densovirinae	NP_958099	2,00E-60	31%	86%	NS
					GAMM01015958	434 (nt)	TSA	Periplaneta fuliginosa densovirus	Ambidensovirus	Densovirinae	NP_051017	1,00E-05	43%	41%	NS
Arthropoda	Insecta	Coleoptera	Carabidae	Pogonus chalceus	JU441893	1499 (nt)	TSA	Acheta domesticus mini ambidensovirus	Ambidensovirus	Densovirinae	YP_008658570	2,00E-07	41%	16%	VP
					JU441895	1334 (nt)	TSA	Acheta domesticus mini ambidensovirus	Ambidensovirus	Densovirinae	YP_008658570	2,00E-07	41%	18%	VP
Arthropoda	Insecta	Coleoptera	Cupedidae	Priacma serrata	GACO01003330	742 (nt)	TSA	Helicoverpa armigera densovirus	Iteradensovirus	Densovirinae	YP_004678720	2,00E-33	36%	90%	NS
Arthropoda	Insecta	Coleoptera	Curculionidae	Ips typographus	GACR01001580	1494 (nt)	TSA	Periplaneta fuliginosa densovirus	Ambidensovirus	Densovirinae	NP_051016	[illegible]	[illegible]	[illegible]	VP
Arthropoda	Insecta	Coleoptera	Curculionidae	Pissodes strobi	GT294677	723 (nt)	EST	Culex pipiens densovirus	Ambidensovirus	Densovirinae	YP_002887625	1,00E-12	40%	57%	NS
					GAED01002887	1190 (nt)	TSA	Culex pipiens densovirus	Ambidensovirus	Densovirinae	YP_002887625	2,00E-12	40%	34%	NS
Arthropoda	Insecta	Coleoptera	Nitidulidae	Meligethes aeneus	GAFB01003071	336 (nt)	TSA	Periplaneta fuliginosa densovirus	Ambidensovirus	Densovirinae	NP_051016	2,00E-18	48%	84%	VP
Arthropoda	Insecta	Coleoptera	Scarabaeidae	Onthophagus nigriventris	GAQW01056793	415 (nt)	TSA	Bombyx mori densovirus 5	Iteradensovirus	Densovirinae	NP_694834	4,00E-24	61%	81%	NS
Arthropoda	Insecta	Diptera	Culicidae	Culex pipiens	x	131 (nt)	RNAseq	Culex pipiens densovirus	Ambidensovirus	Densovirinae	YP_002887627	2,00E-17	88%	99%	VP
					x	131 (nt)	RNAseq	Culex pipiens densovirus	Iteradensovirus	Densovirinae	YP_002887627	2,00E-10	74%	96%	VP
					x	123 (nt)	RNAseq	Culex pipiens densovirus	Ambidensovirus	Densovirinae	YP_002887625	1,00E-19	100%	97%	NS
Arthropoda	Insecta	Diptera	Diopsidae	Teleopsis dalmanni	GBBP01040481	482 (nt)	TSA	Mythimna loreyi densovirus	Ambidensovirus	Densovirinae	NP_958099	7,00E-39	51%	85%	NS
Arthropoda	Insecta	Diptera	Drosophilidae	Drosophila ananassae	EB577555	817 (nt)	EST	Mouse parvovirus 5a	Protoparvovirus	Parvovirinae	YP_002332920	3,00E-133	96%	74%	NS
Arthropoda	Insecta	Diptera	Drosophilidae	Drosophila persimilis	HM469391	673 (nt)	nr	Solenopsis invicta densovirus	Ambidensovirus	Densovirinae	YP_008766862	2,00E-04	35%	34%	NS
					ADQ55747	204 (aa)	nr	Solenopsis invicta densovirus	Ambidensovirus	Densovirinae	YP_008766862	2,00E-06	39%	32%	NS
Arthropoda	Insecta	Diptera	Drosophilidae	Drosophila sechellia	DK330728	424 (nt)	EST	Helicoverpa armigera densovirus	Iteradensovirus	Densovirinae	AEI26255	[illegible]	[illegible]	[illegible]	NS
					DK334580	286 (nt)	EST	Helicoverpa armigera densovirus	Iteradensovirus	Densovirinae	AEI26255	[illegible]	[illegible]	[illegible]	NS
					DK312665	410 (nt)	EST	Periplaneta fuliginosa densovirus	Ambidensovirus	Densovirinae	NP_051016	[illegible]	[illegible]	[illegible]	VP
					DK318448	410 (nt)	EST	Periplaneta fuliginosa densovirus	Ambidensovirus	Densovirinae	NP_051016	[illegible]	[illegible]	[illegible]	VP
Arthropoda	Insecta	Diptera	Syrphidae	Eristalis tenax	CAM92106	230 (aa)	nr	Densovirus SC444	Ambidensovirus	Densovirinae	AFH02751	3,00E-05	39%	34%	VP
Arthropoda	Insecta	Diptera	Tephritidae	Bactrocera dorsalis	GAKP01023238	748 (nt)	TSA	Dendrolimus punctatus densovirus	Iteradensovirus	Densovirinae	YP_164339	5,00E-08	27%	73%	NS
					GAKP01023412	1070 (nt)	TSA	Drosophila persimilis nonstructural-like protein	Ambidensovirus	Densovirinae	ADQ55747	1,00E-29	48%	36%	NS
Arthropoda	Insecta	Diptera	Tephritidae	Rhagoletis pomonella	EZ131500	170 (nt)	TSA	Serpentine adeno-associated virus 2	Dependoparvovirus	Densovirinae	ACJ66591	4,00E-05	61%	58%	VP
Arthropoda	Insecta	Hemiptera	Aleyrodidae	Bemisia tabaci	EZ941734	374 (nt)	TSA	Mythimna loreyi densovirus	Iteradensovirus	Densovirinae	NP_958099	2,00E-05	53%	40%	NS
					EZ941358	360 (nt)	TSA	Blattella germanica densovirus	Ambidensovirus	Densovirinae	NP_874381	5,00E-22	45%	90%	NS
					EZ949243	187 (nt)	TSA	Pseudoplusia includens densovirus	Ambidensovirus	Densovirinae	YP_007003823	7,00E-05	50%	78%	NS
					EZ945577	125 (nt)	TSA	Periplaneta fuliginosa densovirus	Ambidensovirus	Densovirinae	NP_051016	2,00E-06	63%	96%	NS
					EZ966062	1172 (nt)	TSA	Blattella germanica densovirus	Ambidensovirus	Densovirinae	NP_874381	[illegible]	[illegible]	[illegible]	NS
					HP650670	211 (nt)	TSA	Helicoverpa armigera densovirus	Iteradensovirus	Densovirinae	AEI26255	[illegible]	[illegible]	[illegible]	NS
					HP645250	153 (nt)	TSA	Mythimna loreyi densovirus	Iteradensovirus	Densovirinae	NP_958099	3,00E-04	36%	96%	NS
Arthropoda	Insecta	Hemiptera	Aphididae	Acyrthosiphon pisum	XM_003241298	3145 (nt)	nr	Dysaphis plantaginea densovirus	Ambidensovirus	Densovirinae	ACG50803	7,00E-141	52%	43%	NS-VP
					CV848413	918 (nt)	EST	Dysaphis plantaginea densovirus	Ambidensovirus	Densovirinae	FJ040397	5,00E-14	87%	8%	NS
					CV837559	877 (nt)	EST	Dysaphis plantaginea densovirus	Ambidensovirus	Densovirinae	ACG50803	2,00E-59	61%	69%	NS
					J9M817	589 (aa)	UNIPROT	Myzus persicae densovirus	Ambidensovirus	Densovirinae	NP_874377	2,00E-108	37%	89%	VP
					J9M820	540 (aa)	UNIPROT	Myzus persicae densovirus	Ambidensovirus	Densovirinae	NP_874376	2,00E-11	63%	32%	NS
					XP_003246947	539 (aa)	nr	Dysaphis plantaginea densovirus	Ambidensovirus	Densovirinae	ACG50803	7,00E-113	41%	88%	NS
					J9LTF9	529 (aa)	UNIPROT	Dysaphis plantaginea densovirus	Ambidensovirus	Densovirinae	ACI01073	2,00E-49	57%	27%	NS
					XP_003247000	523 (aa)	nr	Myzus persicae densovirus	Ambidensovirus	Densovirinae	NP_874375	7,00E-11	39%	15%	NS
					J9LIS5	471 (aa)	UNIPROT	Dysaphis plantaginea densovirus	Ambidensovirus	Densovirinae	ACG50803	1,00E-36	54%	49%	NS
					J9KHY5	453 (aa)	UNIPROT	Acyrthosiphon pisum densovirus	Ambidensovirus	Densovirinae	XP_001949504	5,00E-21	29%	50%	VP
					J9LDI1	443 (aa)	UNIPROT	Dysaphis plantaginea densovirus	Ambidensovirus	Densovirinae	ACG50803	1,00E-52	61%	33%	NS
					J9M818	440 (aa)	UNIPROT	Myzus persicae densovirus	Ambidensovirus	Densovirinae	NP_874377	5,00E-35	32%	88%	VP
					J9LDN6	404 (aa)	UNIPROT	Dysaphis plantaginea densovirus	Ambidensovirus	Densovirinae	ACG50803	7,00E-30	58%	26%	NS
					J9M024	422 (aa)	UNIPROT	Dysaphis plantaginea densovirus	Ambidensovirus	Densovirinae	ACI01073	7,00E-08	52%	10%	NS
					J9M821	368 (aa)	UNIPROT	Myzus persicae densovirus	Ambidensovirus	Densovirinae	NP_874375	3,00E-17	34%	39%	NS
					J9LVB6	355 (aa)	UNIPROT	Acyrthosiphon pisum densovirus	Ambidensovirus	Densovirinae	XP_003244155	3,00E-20	36%	49%	VP
					XP_003243220	330 (aa)	nr	Myzus persicae densovirus	Ambidensovirus	Densovirinae	NP_874375	8,00E-10	36%	26%	NS
					J9LIS4	315 (aa)	UNIPROT	Dysaphis plantaginea densovirus	Ambidensovirus	Densovirinae	ACG50803	2,00E-36	57%	58%	NS
					J9KDT0	311 (aa)	UNIPROT	Myzus persicae densovirus	Ambidensovirus	Densovirinae	NP_874375	3,00E-21	38%	46%	NS
					J9KNE2	278 (aa)	UNIPROT	Myzus persicae densovirus	Ambidensovirus	Densovirinae	NP_874377	3,00E-35	32%	90%	VP
					J9LGW7	275 (aa)	UNIPROT	Myzus persicae densovirus	Ambidensovirus	Densovirinae	NP_874377	1,00E-08	53%	23%	VP
					J9KQJ4	245 (aa)	UNIPROT	Myzus persicae densovirus	Ambidensovirus	Densovirinae	NP_874377	6,00E-68	49%	97%	VP
					J9LIS2	233 (aa)	UNIPROT	Myzus persicae densovirus	Ambidensovirus	Densovirinae	NP_874377	5,00E-38	36%	97%	VP
					J9LTF8	233 (aa)	UNIPROT	Myzus persicae densovirus	Ambidensovirus	Densovirinae	NP_874377	5,00E-24	31%	91%	VP
					J9LFQ1	230 (aa)	UNIPROT	Myzus persicae densovirus	Ambidensovirus	Densovirinae	NP_874377	3,00E-14	43%	40%	VP
					J9KN63	229 (aa)	UNIPROT	Dysaphis plantaginea densovirus	Ambidensovirus	Densovirinae	ACG50803	1,00E-56	62%	65%	NS
					J9KXT4	128 (aa)	UNIPROT	Dysaphis plantaginea densovirus	Ambidensovirus	Densovirinae	ACG50806	1,00E-08	50%	77%	VP
					J9KT31	181 (aa)	UNIPROT	Myzus persicae densovirus	Ambidensovirus	Densovirinae	NP_874377	3,00E-10	29%	74%	VP
					J9LTF7	114 (aa)	UNIPROT	Myzus persicae densovirus	Ambidensovirus	Densovirinae	NP_874377	3,00E-07	43%	61%	VP
					J9LBV1	87 (aa)	UNIPROT	Myzus persicae densovirus	Ambidensovirus	Densovirinae	NP_874376	4,00E-06	65%	39%	NS
					J9LQZ1	78 (aa)	UNIPROT	Myzus persicae densovirus	Ambidensovirus	Densovirinae	NP_874377	2,00E-10	56%	83%	VP
Arthropoda	Insecta	Hemiptera	Aphididae	Sitobion avenae	GAPL01021454.1	1836 (nt)	TSA	Myzus persicae densovirus	Ambidensovirus	Densovirinae	NP_874376.1	4,00E-157	69%	88%	NS
Arthropoda	Insecta	Hemiptera	Cicadellidae	Graminella nigrifrons	GAQX01015625	714 (nt)	TSA	Dendrolimus punctatus densovirus	Iteradensovirus	Densovirinae	YP_164339	2,00E-29	38%	88%	NS
Arthropoda	Insecta	Hemiptera	Coreidae	Anoplocnemis curvipes	GAJV01009079	913 (nt)	TSA	Periplaneta fuliginosa densovirus	Ambidensovirus	Densovirinae	NP_051016	2,00E-18	56%	28%	VP
Arthropoda	Insecta	Hemiptera	Coreidae	Clavigralla tomentosicollis	GAJX01007999	365 (nt)	TSA	Periplaneta fuliginosa densovirus	Ambidensovirus	Densovirinae	NP_051016	5,00E-19	52%	71%	VP
Arthropoda	Insecta	Hemiptera	Delphacidae	Nilaparvata lugens	HS466630	168 (nt)	EST	Periplaneta fuliginosa densovirus	Ambidensovirus	Densovirinae	NP_051016	3,00E-07	50%	78%	VP
					GANM01012737	725 (nt)	TSA	Periplaneta fuliginosa densovirus	Ambidensovirus	Densovirinae	NP_051016	7,00E-19	48%	37%	VP
					HS458495	147 (nt)	EST	Rat adeno-associated virus 1	Dependoparvovirus	Parvovirinae	AAZ79676	4,00E-07	58%	91%	NS
Arthropoda	Insecta	Hemiptera	Kerriidae	Kerria lacca	GBDP01003119	1354 (nt)	TSA	Helicoverpa armigera densovirus	Iteradensovirus	Densovirinae	AEI26255	9,00E-30	41%	51%	NS
					GBDP01040339	966 (nt)	TSA	Helicoverpa armigera densovirus	Iteradensovirus	Densovirinae	YP_004678720	2,00E-37	35%	78%	NS
					GBDP01000144	964 (nt)	TSA	Helicoverpa armigera densovirus	Iteradensovirus	Densovirinae	YP_004678720	2,00E-37	35%	79%	NS
					GBDO01037141	681 (nt)	TSA	Culex pipiens densovirus	Ambidensovirus	Densovirinae	YP_002887625	2,00E-28	47%	73%	NS
					GBDO01030308	1259 (nt)	TSA	Helicoverpa armigera densovirus	Iteradensovirus	Densovirinae	YP_004678720	4,00E-34	32%	61%	NS
Arthropoda	Insecta	Hemiptera	Miridae	Lygus hesperus	GBHO01030277.1	2175 (nt)	TSA	Helicoverpa armigera densovirus	Iteradensovirus	Densovirinae	YP_004678721.1	5,00E-28	28%	71%	VP
					A0A0A9VS64	241 (aa)	UNIPROT	Periplaneta fuliginosa densovirus	Ambidensovirus	Densovirinae	NP_051016.1	9,00E-17	46%	37%	VP
					A0A0A9VW96	109 (aa)	UNIPROT	Acheta domesticus mini ambidensovirus	Ambidensovirus	Densovirinae	YP_008658570.1	3,00E-15	49%	76%	VP
					A0A0A9W115	215 (aa)	UNIPROT	Periplaneta fuliginosa densovirus	Ambidensovirus	Densovirinae	NP_051016.1	5,00E-17	46%	42%	VP
					A0A0A9XIJ7	249 (aa)	UNIPROT	Periplaneta fuliginosa densovirus	Ambidensovirus	Densovirinae	NP_051016.1	2,00E-11	43%	36%	VP
					A0A0A9YFR6	114 (aa)	UNIPROT	Dendrolimus punctatus densovirus	Iteradensovirus	Densovirinae	YP_164339.1	7,00E-17	51%	66%	NS
					A0A0A9YTR0	563 (aa)	UNIPROT	Helicoverpa armigera densovirus	Iteradensovirus	Densovirinae	YP_004678720.1	1,00E-57	31%	73%	NS
					A0A0K8T346	708 (aa)	UNIPROT	Acheta domesticus mini ambidensovirus	Ambidensovirus	Densovirinae	YP_008658570.1	1,00E-14	51%	12%	VP
					A0A0A9VS60	103 (aa)	UNIPROT	Periplaneta fuliginosa densovirus	Ambidensovirus	Densovirinae	NP_051016.1	6,00E-06	48%	52%	VP
					A0A0A9WXX8	703 (aa)	UNIPROT	Helicoverpa armigera densovirus	Iteradensovirus	Densovirinae	YP_004678721.1	2,00E-33	28%	81%	VP
					A0A0A9VST1	244 (aa)	UNIPROT	Periplaneta fuliginosa densovirus	Ambidensovirus	Densovirinae	NP_051016.1	4,00E-16	48%	35%	VP
Arthropoda	Insecta	Hemiptera	Pentatomidae	Chinavia ubica	GBFA01009334	369 (nt)	TSA	Periplaneta fuliginosa densovirus	Ambidensovirus	Densovirinae	NP_051016	5,00E-11	48%	61%	VP
Arthropoda	Insecta	Hemiptera	Pentatomidae	Halyomorpha halys	GBHT01005998	684 (nt)	TSA	Periplaneta fuliginosa densovirus	Ambidensovirus	Densovirinae	BAA82965	1,00E-18	51%	41%	VP
					GBHT01013004	5603 (nt)	TSA	Blattella germanica densovirus-like virus	Ambidensovirus	Densovirinae	AFC75858	4,00E-24	33%	15%	NS-VP
Arthropoda	Insecta	Hemiptera	Psyllidae	Diaphorina citri	XM_008484718	874 (nt)	nr	Dendrolimus punctatus densovirus	Iteradensovirus	Densovirinae	YP_164339.1	1,00E-04	25%	83%	NS
					XP_008482940	290 (aa)	nr	Dendrolimus punctatus densovirus	Iteradensovirus	Densovirinae	YP_164339	5,00E-07	26%	83%	NS
Arthropoda	Insecta	Hemiptera	Psyllidae	Pachypsylla venusta	GAOP01006963	221 (nt)	TSA	Goose parvovirus	Dependoparvovirus	Parvovirinae	ABU50789	8,00E-07	49%	61%	VP
					GAOP01032067	628 (nt)	TSA	Periplaneta fuliginosa densovirus	Ambidensovirus	Densovirinae	NP_051016	2,00E-09	42%	41%	VP
					GAOP01141099	213 (nt)	TSA	Porcine bocavirus 3	Bocaparvovirus	Parvovirinae	AHX26965	2,00E-07	62%	59%	VP
Arthropoda	Insecta	Hemiptera	Reduviidae	Rhodnius prolixus	T1I545	622 (aa)	UNIPROT	Blattella germanica densovirus-like virus	Ambidensovirus	Densovirinae	AFC75858	1,00E-12	45%	11%	VP
					T1I3J4	551 (aa)	UNIPROT	Acheta domesticus mini ambidensovirus	Ambidensovirus	Densovirinae	YP_008658570	5,00E-13	24%	76%	VP
					T1I3J1	403 (aa)	UNIPROT	Dendrolimus punctatus densovirus	Iteradensovirus	Densovirinae	YP_164339	3,00E-22	35%	37%	NS
					T1I3J3	320 (aa)	UNIPROT	Aedes albopictus C6/36 cell densovirus	Brevidensovirus	Densovirinae	AAP78907	3,00E-38	44%	56%	NS
					T1HE17	293 (aa)	UNIPROT	Acheta domesticus mini ambidensovirus	Ambidensovirus	Densovirinae	YP_008658570	4,00E-06	25%	94%	VP
					T1HRN5	254 (aa)	UNIPROT	Periplaneta fuliginosa densovirus	Ambidensovirus	Densovirinae	NP_051016	7,00E-21	54%	34%	VP
					T1HE18	230 (aa)	UNIPROT	Blattella germanica densovirus	Ambidensovirus	Densovirinae	NP_874381	7,00E-10	44%	33%	NS
Arthropoda	Insecta	Hemiptera	Reduviidae	Triatoma infestans	GBBI01004972	1695 (nt)	TSA	Bombyx mori densovirus 5	Iteradensovirus	Densovirinae	NP_694834	2,00E-27	26%	68%	NS
					GBBI01004379	366 (nt)	TSA	Acheta domesticus mini ambidensovirus	Ambidensovirus	Densovirinae	YP_008658570	6,00E-06	31%	88%	VP
Arthropoda	Insecta	Hymenoptera	Apidae	Bombus impatiens	JK097654	333 (nt)	TSA	Helicoverpa armigera densovirus	Iteradensovirus	Densovirinae	AEI26255	1,00E-24	44%	98%	NS
Arthropoda	Insecta	Hymenoptera	Formicidae	Acromyrmex echinatior	XM_011070319.1	2013 (nt)	nr	Culex densovirus 0507J511	Brevidensovirus	Densovirinae	ACS70953.1	4,00E-50	29%	82%	NS
					XP_011068621.1	520 (aa)	nr	Culex densovirus 0507J511	Brevidensovirus	Densovirinae	ACS70953.1	1,00E-49	31%	96%	NS
					F4W8Y7	420 (aa)	UNIPROT	Mosquito densovirus HB-3	Brevidensovirus	Densovirinae	KM975734.1	2,00E-13	40%	23%	NS
					F4WA07	838 (aa)	UNIPROT	Aedes densovirus (strain GKV 002 002)	Brevidensovirus	Densovirinae	P40784	6,00E-22	34%	24%	NS
					EGI68937	838 (aa)	nr	Culex pipiens pallens densovirus	Brevidensovirus	Densovirinae	ABU94988	7,00E-22	33%	25%	NS-VP
					EGI69346	420 (aa)	nr	Mosquito densovirus BR/07	Brevidensovirus	Densovirinae	YP_004222724	3,00E-15	30%	42%	NS
Arthropoda	Insecta	Hymenoptera	Formicidae	Atta cephalotes	XM_012209122.1	2574 (nt)	nr	Mosquito densovirus HB-3	Brevidensovirus	Densovirinae	AII02597.1	5,00E-40	29%	73%	NS
					H9K5W1	430 (aa)	UNIPROT	Anopheles gambiae densonucleosis virus	Brevidensovirus	Densovirinae	EU233812.1	7,00E-04	31%	29%	NS
					H9VWX9	411 (aa)	UNIPROT	Haemagogus equinus densovirus	Brevidensovirus	Densovirinae	AAT35223.1	8,00E-22	27%	78%	NS
					XP_012064512.1	857 (aa)	nr	Mosquito densovirus HB-3	Brevidensovirus	Densovirinae	AII02597.1	2,00E-48	30%	70%	NS
Arthropoda	Insecta	Hymenoptera	Formicidae	Lasius niger	A0A0J7JUP7	201 (aa)	UNIPROT	Blattella germanica densovirus-like virus	Ambidensovirus	Densovirinae	AFC75861.1	1,00E-40	41%	99%	VP
					KMQ81887.1	201 (aa)	nr	Blattella germanica densovirus-like virus	Ambidensovirus	Densovirinae	AFC75861.1	1,00E-40	41%	99%	VP
Arthropoda	Insecta	Hymenoptera	Formicidae	Messor barbarus	x	506 (nt)	RNAseq	Mosquito densovirus BR/07	Brevidensovirus	Densovirinae	YP_004222724	[illegible]	[illegible]	97%	NS
					x	510 (nt)	RNAseq	Mosquito densovirus BR/07	Brevidensovirus	Densovirinae	YP_004222724	[illegible]	[illegible]	85%	NS
					x	336 (nt)	RNAseq	Culex densovirus 0507J511	Brevidensovirus	Densovirinae	ACS70953	8,00E-16	45%	86%	NS
					x	140 (nt)	RNAseq	Bombyx mori densovirus 5	Iteradensovirus	Densovirinae	NP_955626	5,00E-17	85%	98%	VP3
Arthropoda	Insecta	Hymenoptera	Formicidae	Messor concolor	x	250 (nt)	RNAseq	Culex pipiens pallens densovirus	Brevidensovirus	Densovirinae	ABU94990	3,00E-13	49%	98%	NS
Arthropoda	Insecta	Hymenoptera	Formicidae	Monomorium pharaonis	XP_012521507.1	738 (aa)	nr	Densovirus SC1065	Ambidensovirus	Densovirinae	AFH02754.1	1,00E-73	52%	36%	VP
					XM_012666055.1	2518 (nt)	nr	Densovirus SC1065	Ambidensovirus	Densovirinae	AFH02754.1	1,00E-66	54%	27%	VP
					XM_012666054.1	2518 (nt)	nr	Densovirus SC1065	Ambidensovirus	Densovirinae	AFH02754.1	1,00E-66	54%	27%	VP
					XM_012666053.1	2520 (nt)	nr	Densovirus SC1065	Ambidensovirus	Densovirinae	AFH02754.1	1,00E-66	54%	27%	VP
Arthropoda	Insecta	Hymenoptera	Formicidae	Pogonomyrmex barbatus	XM_011645096.1	2554 (nt)	nr	Densovirus SC1065	Ambidensovirus	Densovirinae	AFH02754.1	5,00E-53	54%	30%	VP
					XM_011645095.1	2573 (nt)	nr	Densovirus SC1065	Ambidensovirus	Densovirinae	AFH02754.1	6,00E-53	54%	30%	VP
					XP_011643397.1	277 (aa)	nr	Periplaneta fuliginosa densovirus	Ambidensovirus	Densovirinae	NP_051016.1	1,00E-16	50%	30%	VP
Arthropoda	Insecta	Hymenoptera	Formicidae	Tetramorium bicarinatum	GASM01014809.1	4856 (nt)	TSA	Blattella germanica densovirus-like virus	Ambidensovirus	Densovirinae	AFC75860.1	0.0	98%	36%	VP
Arthropoda	Insecta	Lepidoptera	Crambidae	Chilo suppressalis	GAJS01056415	1864 (nt)	TSA	Densovirus SC444	Ambidensovirus	Densovirinae	AFH02751	8,00E-15	35%	26%	NS-VP
					GAJS01023148	852 (nt)	TSA	Aleutian mink disease virus	Amdoparvovirus	Parvovirinae	ACX31241	1,00E-07	32%	38%	NS
					GAJS01033341	262 (nt)	TSA	Culex pipiens densovirus	Ambidensovirus	Densovirinae	YP_002887625	5,00E-05	33%	91%	NS
Arthropoda	Insecta	Lepidoptera	Micropterigidae	Micropterix calthella	GACP01013448	797 (nt)	TSA	Helicoverpa armigera densovirus	Iteradensovirus	Densovirinae	AFK91983	2,00E-15	38%	44%	VP
					GACP01050464.1	446 (nt)	TSA	Culex pipiens densovirus	Ambidensovirus	Densovirinae	YP_002887625.1	4,00E-21	49%	68%	NS
					GACP01062115.1	387 (nt)	TSA	Culex pipiens densovirus	Ambidensovirus	Densovirinae	YP_002887626.1	6,00E-07	46%	47%	NS
					GACP01000056	2168 (nt)	TSA	Culex pipiens densovirus	Ambidensovirus	Densovirinae	YP_002887627	1,00E-18	40%	16%	VP
Arthropoda	Insecta	Lepidoptera	Noctuidae	Agrotis segetum	GBCW01052843	214 (nt)	TSA	Porcine parvovirus	Protoparvovirus	Parvovirinae	AFV25463	1,00E-05	59%	57%	VP
Arthropoda	Insecta	Lepidoptera	Noctuidae	Helicoverpa armigera	GBDM01002181.1	2011 (nt)	TSA	Helicoverpa armigera densovirus	Iteradensovirus	Densovirinae	YP_004678720.1	0.0	99%	99%	NS
Arthropoda	Insecta	Lepidoptera	Nymphalidae	Bicyclus anynana	GE699248	710 (nt)	EST	Helicoverpa armigera densovirus	Iteradensovirus	Densovirinae	AEI26255	3,00E-45	41%	83%	NS
Arthropoda	Insecta	Lepidoptera	Thaumetopoeidae	Thaumetopoea pityocampa	GBXZ01000030.1	3129 (nt)	TSA	Sibine fusca densovirus	Iteradensovirus	Densovirinae	YP_006576512.1	0.0	90%	37%	NS
					GBXZ01001450.1	2084 (nt)	TSA	Danaus plexippus plexippus iteravirus	Iteradensovirus	Densovirinae	YP_009021038.1	0.0	84%	97%	VP
					GBZB01000084.1	1473 (nt)	TSA	Sibine fusca densovirus	Iteradensovirus	Densovirinae	YP_006576512.1	0.0	89%	98%	NS
					GBZB01000242.1	2075 (nt)	TSA	Danaus plexippus plexippus iteravirus	Iteradensovirus	Densovirinae	YP_009021038.1	0.0	84%	98%	VP
Arthropoda	Insecta	Mecoptera	Nannochoristidae	Nannochorista philpotti	GAOB01022500	479 (nt)	TSA	Diatraea saccharalis densovirus	Ambidensovirus	Densovirinae	NP_046815	2,00E-09	36%	66%	VP
					GAOB01026327	410 (nt)	TSA	Acheta domesticus mini ambidensovirus	Ambidensovirus	Densovirinae	YP_008658570	9,00E-13	41%	76%	VP
Arthropoda	Insecta	Megaloptera	Corydalidae	Corydalinae sp.	GADH01010824	703 (nt)	TSA	Junonia coenia densovirus	Ambidensovirus	Densovirinae	NP_694824	2,00E-102	65%	99%	NS
					GADH01005272.1	878 (nt)	TSA	Culex pipiens densovirus	Ambidensovirus	Densovirinae	YP_002887625.1	7,00E-73	49%	89%	NS
					GADH01024644.1	491 (nt)	TSA	Diatraea saccharalis densovirus	Ambidensovirus	Densovirinae	NP_046818.1	3,00E-15	43%	56%	VP
Arthropoda	Insecta	Neuroptera	Chrysopidae	Chrysopa pallens	GAGF01035954	271 (nt)	TSA	Periplaneta fuliginosa densovirus	Ambidensovirus	Densovirinae	BAA82965	3,00E-12	48%	70%	VP
Arthropoda	Insecta	Orthoptera	Acrididae	Schistocerca gregaria	JG678612	514 (nt)	EST	Planococcus citri densovirus	Ambidensovirus	Densovirinae	NP_694843	1,00E-22	35%	99%	NS
					JG668850	877 (nt)	EST	Acheta domestica densovirus	Ambidensovirus	Densovirinae	YP_227600	2,00E-18	39%	81%	NS
					JG696318	756 (nt)	EST	Planococcus citri densovirus	Ambidensovirus	Densovirinae	NP_694843	4,00E-24	42%	61%	NS
Arthropoda	Insecta	Orthoptera	Gryllidae	Gryllus bimaculatus	BY997974	857 (nt)	EST	Blattella germanica densovirus	Ambidensovirus	Densovirinae	NP_874381	1,00E-53	51%	73%	NS
Arthropoda	Insecta	Phasmatodea	Heteropterygidae	Aretaon asperrimus	GAWC01012962	2326 (nt)	TSA	Acheta domesticus mini ambidensovirus	Brevidensovirus	Densovirinae	YP_008658568	2,00E-35	27%	59%	NS
					GAWC01079969	1301 (nt)	TSA	Haemagogus equinus densovirus	Brevidensovirus	Densovirinae	AAT35224	1,00E-04	29%	26%	NS
					GAWC01014089	961 (nt)	TSA	Aedes albopictus C6/36 cell densovirus	Brevidensovirus	Densovirinae	AAM28943	5,00E-09	31%	56%	NS
					GAWC01079963	744 (nt)	TSA	Haemagogus equinus densovirus	Brevidensovirus	Densovirinae	AAT35224	3,00E-06	30%	50%	NS
					GAWC01079979	559 (nt)	TSA	Haemagogus equinus densovirus	Brevidensovirus	Densovirinae	AAT35224	3,00E-04	31%	60%	NS
					GAWC01033483	488 (nt)	TSA	Aedes albopictus C6/36 cell densovirus	Brevidensovirus	Densovirinae	AAP78907	7,00E-19	38%	85%	NS
					GAWC01061961	328 (nt)	TSA	Acheta domesticus mini ambidensovirus	Brevidensovirus	Densovirinae	YP_008658570	3,00E-06	38%	86%	VP
					GAWC01112458	260 (nt)	TSA	Culex pipiens pallens densovirus	Brevidensovirus	Densovirinae	ABU94990.1	3,00E-11	44%	93%	NS
					GAWC01115021	238 (nt)	TSA	Aedes albopictus C6/36 cell densovirus	Brevidensovirus	Densovirinae	AAP78907	4,00E-16	53%	98%	NS
					GAWC01079975	9205 (nt)	TSA	Culex pipiens pallens densovirus	Brevidensovirus	Densovirinae	ABU94987	4,00E-41	31%	12%	NS
					GAWC01079978	6252 (nt)	TSA	Culex pipiens pallens densovirus	Brevidensovirus	Densovirinae	ABU94996	[illegible]	[illegible]	[illegible]	NS-VP
					GAWC01079972	1286 (nt)	TSA	Haemagogus equinus densovirus	Brevidensovirus	Densovirinae	AAT35224	[illegible]	[illegible]	[illegible]	NS
Arthropoda	Insecta	Phasmatodea	Heteronemiidae	Sipyloidea sipylus	GAWF01042485	4237 (nt)	TSA	Periplaneta fuliginosa densovirus	Ambidensovirus	Densovirinae	NP_051016	2,00E-04	42%	5%	VP
Arthropoda	Insecta	Phasmatodea	Phasmatidae	Extatosoma tiaratum	GAWG01068362	2554 (nt)	TSA	Acheta domesticus mini ambidensovirus	Brevidensovirus	Densovirinae	YP_008658568	7,00E-10	43%	12%	NS
					GAWG01068359	2531 (nt)	TSA	Acheta domesticus mini ambidensovirus	Brevidensovirus	Densovirinae	YP_008658568	7,00E-10	43%	12%	NS
					GAWG01068354	2381 (nt)	TSA	Acheta domesticus mini ambidensovirus	Brevidensovirus	Densovirinae	YP_008658568	7,00E-10	43%	13%	NS
					GAWG01046839	1022 (nt)	TSA	Acheta domesticus mini ambidensovirus	Brevidensovirus	Densovirinae	YP_008658568	2,00E-20	41%	40%	NS
					GAWG01044928	873 (nt)	TSA	Acheta domesticus mini ambidensovirus	Brevidensovirus	Densovirinae	YP_008658570	2,00E-13	33%	70%	VP
					GAWG01112514	520 (nt)	TSA	Acheta domesticus mini ambidensovirus	Brevidensovirus	Densovirinae	YP_008658568	2,00E-22	34%	97%	NS
					GAWG01035049	468 (nt)	TSA	Acheta domesticus mini ambidensovirus	Brevidensovirus	Densovirinae	YP_008658570	5,00E-09	45%	38%	VP
					GAWG01032598	334 (nt)	TSA	Acheta domesticus mini ambidensovirus	Brevidensovirus	Densovirinae	YP_008658570	9,00E-15	38%	86%	VP
					GAWG01125783	818 (nt)	TSA	Culex pipiens pallens densovirus	Brevidensovirus	Densovirinae	ABU94987	1,00E-05	39%	33%	NS
					GAWG01127082	646 (nt)	TSA	Culex pipiens pallens densovirus	Brevidensovirus	Densovirinae	ABU94966	3,00E-14	28%	84%	NS
					GAWG01117743	644 (nt)	TSA	Aedes albopictus C6/36 cell densovirus	Brevidensovirus	Densovirinae	AAP78907	3,00E-36	50%	75%	NS
					GAWG01002294	624 (nt)	TSA	Aedes aegypti densovirus	Brevidensovirus	Densovirinae	YP_002854231	1,00E-05	34%	79%	NS
					GAWG01117729	522 (nt)	TSA	Aedes albopictus C6/36 cell densovirus	Brevidensovirus	Densovirinae	AAM28944	5,00E-06	30%	67%	NS
					GAWG01139811	410 (nt)	TSA	Culex pipiens densovirus	Ambidensovirus	Densovirinae	AFH02734	7,00E-13	47%	54%	NS
					GAWG01115162	384 (nt)	TSA	Haemagogus equinus densovirus	Brevidensovirus	Densovirinae	AAT35223	3,00E-18	46%	77%	NS
					GAWG01005303	304 (nt)	TSA	Aedes albopictus C6/36 cell densovirus	Brevidensovirus	Densovirinae	AAP78907	5,00E-19	51%	87%	NS
					GAWG01003140	317 (nt)	TSA	Aedes albopictus C6/36 cell densovirus	Brevidensovirus	Densovirinae	AAM28944	4,00E-06	35%	85%	NS
					GAWG01006018	300 (nt)	TSA	Aedes albopictus C6/36 cell densovirus	Brevidensovirus	Densovirinae	AAP78907	2,00E-20	48%	99%	NS
					GAWG01003945	292 (nt)	TSA	Aedes albopictus C6/36 cell densovirus	Brevidensovirus	Densovirinae	AAM28943	5,00E-21	51%	96%	NS
Arthropoda	Insecta	Phasmatodea	Phasmatidae	Medauroidea extradentata	GAWD01093153	587 (nt)	TSA	Aedes albopictus C6/36 cell densovirus	Brevidensovirus	Densovirinae	AAP78907	9,00E-38	43%	88%	NS
					GAWD01089640	387 (nt)	TSA	Culex pipiens densovirus	Ambidensovirus	Densovirinae	AFH02734	3,00E-04	47%	39%	NS
					GAWD01059795	457 (nt)	TSA	Periplaneta fuliginosa densovirus	Ambidensovirus	Densovirinae	NP_051016	[illegible]	[illegible]	[illegible]	NS
Arthropoda	Insecta	Raphidioptera	Raphidiidae	Raphidia ariadne	GACX01003907	951 (nt)	TSA	Dendrolimus punctatus densovirus	Iteradensovirus	Densovirinae	YP_164339	2,00E-34	32%	73%	NS
Arthropoda	Malacostraca	Amphipoda	Ampeliscidae	Ampelisca abdita	JZ096885	647 (nt)	EST	Periplaneta fuliginosa densovirus	Ambidensovirus	Densovirinae	NP_051020	[illegible]	[illegible]	[illegible]	NS
					JZ093383	590 (nt)	EST	Periplaneta fuliginosa densovirus	Ambidensovirus	Densovirinae	NP_051020	[illegible]	[illegible]	[illegible]	NS
					JZ096796	594 (nt)	EST	Periplaneta fuliginosa densovirus	Ambidensovirus	Densovirinae	NP_051020	[illegible]	[illegible]	[illegible]	NS
					JZ088717	505 (nt)	EST	Periplaneta fuliginosa densovirus	Ambidensovirus	Densovirinae	NP_051020	[illegible]	[illegible]	[illegible]	NS
					JZ093297	619 (nt)	EST	Periplaneta fuliginosa densovirus	Ambidensovirus	Densovirinae	NP_051020	[illegible]	[illegible]	[illegible]	NS
Arthropoda	Malacostraca	Decapoda	Astacidea	Pontastacus leptodactylus	GAFY01011594.1	743 (nt)	TSA	Decapod penstyldensovirus 1	Penstyldensovirus	Densovirinae	AYS1518.1	9,00E-07	36%	96%	NS
Arthropoda	Malacostraca	Decapoda	Palaemonidae	Macrobrachium nipponense	JK528478	1178 (nt)	EST	Casphalia extranea densovirus	Iteradensovirus	Densovirinae	NP_694840	3,00E-09	42%	22%	VP
Arthropoda	Malacostraca	Decapoda	Penaeidae	Penaeus monodon	DQ228358.1	4655 (nt)	nr	Penaeus monodon penstyldensovirus 2	Penstyldensovirus	Densovirinae	AAM93906.1	0.0	87%	42%	NS
					AAM94165.1	666 (aa)	nr	Penaeus monodon penstyldensovirus 2	Penstyldensovirus	Densovirinae	AAM93906.1	0.0	87%	100%	NS
Arthropoda	Malacostraca	Decapoda	Portunidae	Scylla olivacea	GDRN01037122.1	531 (nt)	TSA	Decapod penstyldensovirus 1	Penstyldensovirus	Densovirinae	AFA36168.1	2,00E-53	58%	98%	NS
					GDRN01091983.1	1295 (nt)	TSA	Decapod penstyldensovirus 1	Penstyldensovirus	Densovirinae	ALE60540.1	2,00E-34	49%	35%	NS
Arthropoda	Malacostraca	Isopoda	Armadillidiidae	Armadillidium nasatum	KT714029.1	2088 (nt)	nr	Decapod penstyldensovirus 1	Penstyldensovirus	Densovirinae	AGO44085.1	9,00E-39	27%	58%	NS
					KT714028.1	458 (nt)	nr	Decapod penstyldensovirus 1	Penstyldensovirus	Densovirinae	AFA36168.1	2,00E-12	31%	98%	NS
					KT714027.1	252 (nt)	nr	Decapod penstyldensovirus 1	Penstyldensovirus	Densovirinae	CAI58631.1	3,00E-11	46%	94%	NS
Arthropoda	Malacostraca	Isopoda	Armadillidiidae	Armadillidium vulgare	KM034113.1	523 (nt)	nr	Decapod penstyldensovirus 1	Penstyldensovirus	Densovirinae	AFA36168.1	1,00E-30	46%	82%	NS
					KM034115.1	729 (nt)	nr	Penaeus stylirostris densovirus	Penstyldensovirus	Densovirinae	AID76764.1	2,00E-36	41%	81%	NS
Arthropoda	Maxillopoda	Arguloida	Argulidae	Argulus siamensis	JW946455	2247 (nt)	TSA	Planococcus citri densovirus	Ambidensovirus	Densovirinae	NP_694842	1,00E-52	33%	53%	NS
					JW950843	1505 (nt)	TSA	Planococcus citri densovirus	Ambidensovirus	Densovirinae	NP_694843	4,00E-56	32%	85%	NS
					JW970278	734 (nt)	TSA	Planococcus citri densovirus	Ambidensovirus	Densovirinae	NP_694842	2,00E-27	39%	64%	NS
					JW966945	718 (nt)	TSA	Dysaphis plantaginea densovirus	Ambidensovirus	Densovirinae	ACG50806	2,00E-13	29%	71%	VP
					JW969152	636 (nt)	TSA	Planococcus citri densovirus	Ambidensovirus	Densovirinae	NP_694842	3,00E-09	47%	33%	VP
					JW980412	363 (nt)	TSA	Planococcus citri densovirus	Ambidensovirus	Densovirinae	NP_694843	3,00E-06	41%	62%	NS
					JW950890	2003 (nt)	TSA	Planococcus citri densovirus	Ambidensovirus	Densovirinae	NP_694842	[illegible]	[illegible]	[illegible]	VP
					JW971361	603 (nt)	TSA	Planococcus citri densovirus	Ambidensovirus	Densovirinae	NP_694842	[illegible]	[illegible]	[illegible]	VP
Arthropoda	Maxillopoda	Siphonostomatoida	Caligidae	Caligus rogercresseyi	GAZX01015685	2691 (nt)	TSA	Infectious hypodermal and hematopoietic necrosis virus	Penstyldensovirus	Densovirinae	AGK30043	5,00E-32	26%	43%	NS
					GAZX01042735	488 (nt)	TSA	Infectious hypodermal and hematopoietic necrosis virus	Penstyldensovirus	Densovirinae	AFA36168	3,00E-29	37%	99%	NS
Arthropoda	Maxillopoda	Siphonostomatoida	Caligidae	Lepeophtheirus salmonis	JP336063	408 (nt)	TSA	Infectious hypodermal and hematopoietic necrosis virus	Penstyldensovirus	Densovirinae	AFA36168	3,00E-15	31%	100%	NS
					JP304452	882 (nt)	TSA	Infectious hypodermal and hematopoietic necrosis virus	Penstyldensovirus	Densovirinae	AFA36168	9,00E-05	32%	48%	NS
					JP302515	1147 (nt)	TSA	Infectious hypodermal and hematopoietic necrosis virus	Penstyldensovirus	Densovirinae	AFR11649	1,00E-04	31%	24%	NS
					FK925682	782 (nt)	EST	Infectious hypodermal and hematopoietic necrosis virus	Penstyldensovirus	Densovirinae	AFA36168	6,00E-05	32%	55%	NS
					FK925681	862 (nt)	EST	Infectious hypodermal and hematopoietic necrosis virus	Penstyldensovirus	Densovirinae	AFA36168	8,00E-05	32%	50%	NS
					FK913417	882 (nt)	EST	Infectious hypodermal and hematopoietic necrosis virus	Penstyldensovirus	Densovirinae	AFA36168	9,00E-05	32%	48%	NS
					FK918082	878 (nt)	EST	Infectious hypodermal and hematopoietic necrosis virus	Penstyldensovirus	Densovirinae	AFA36168	6,00E-04	32%	21%	NS
					FK918083	845 (nt)	EST	Infectious hypodermal and hematopoietic necrosis virus	Penstyldensovirus	Densovirinae	AFR11649	4,00E-05	31%	33%	NS
Cnidaria	Anthozoa	Alcyonacea	Gorgoniidae	Eunicella cavolinii	x	1374 (nt)	RNAseq	Clonorchis sinensis non capsid protein NS-1	Penstyldensovirus	Densovirinae	GAA57638	8,00E-11	29%	33%	NS
Echinodermata	Asteroidea	Paxillosida	Luidiidae	Luidia clathrata	GAVQ01079362	449 (nt)	TSA	Dendrolimus punctatus densovirus	Iteradensovirus	Densovirinae	YP_164339	1,00E-09	36%	68%	NS
Echinodermata	Asteroidea	Spinulosida	Echinasteridae	Echinaster spinulosus	GAVE01029446	2405 (nt)	TSA	Culex pipiens densovirus	Ambidensovirus	Densovirinae	YP_002887625	2,00E-17	29%	24%	NS
Echinodermata	Ophiuroidea	Ophiurida	Amphiuridae	Amphiura filiformis	JG390847	303 (nt)	EST	Aleutian mink disease virus	Amdoparvovirus	Parvovirinae	ACG68611	1,00E-62	95%	97%	NS
Mollusca	Cephalopoda	Octopoda	Octopodidae	Octopus bimaculoides	A0A0L8FGN6	172 (aa)	UNIPROT	Dendrolimus punctatus densovirus	Iteradensovirus	Densovirinae	YP_164339.1	1,00E-10	31%	88%	NS
					A0A0L8GWZ8	246 (aa)	UNIPROT	Dendrolimus punctatus densovirus	Iteradensovirus	Densovirinae	YP_164339.1	4,00E-19	32%	66%	NS
					KOF81493.1	246 (aa)	nr	Dendrolimus punctatus densovirus	Iteradensovirus	Densovirinae	YP_164339.1	4,00E-19	32%	66%	NS
					KOF62817.1	172 (aa)	nr	Dendrolimus punctatus densovirus	Iteradensovirus	Densovirinae	YP_164339.1	1,00E-10	31%	88%	NS
Mollusca	Cephalopoda	Octopoda	Octopodidae	Octopus vulgaris	JR446908	565 (nt)	TSA	Adeno-associated virus	Dependoparvovirus	Parvovirinae	AAU05371	1,00E-07	33%	58%	VP
Mollusca	Cephalopoda	Sepiida	Sepiidae	Sepia officinalis	FO193416	657 (nt)	EST	Turkey parvovirus	Aveparvovirus	Parvovirinae	AHF54687	1,00E-08	30%	61%	NS
Mollusca	Cephalopoda	Sepiolida	Sepiolidae	Euprymna scolopes	DW277051	743 (nt)	EST	Turkey parvovirus TP1-2012/HUN	Aveparvovirus	Parvovirinae	AHF54687	4,00E-06	38%	27%	NS
					DW277466	765 (nt)	EST	Turkey parvovirus TP1-2012/HUN	Aveparvovirus	Parvovirinae	AHF54687	8,00E-06	39%	26%	NS
					DW283133	719 (nt)	EST	Turkey parvovirus TP1-2012/HUN	Aveparvovirus	Parvovirinae	AHF54687	7,00E-06	43%	25%	NS
					GBHH01003248	514 (nt)	TSA	Turkey parvovirus TP1-2012/HUN	Aveparvovirus	Parvovirinae	AHF54687	7,00E-10	32%	68%	NS
Mollusca	Gastropoda	x	Bithyniidae	Bithynia siamensis	GAQQ01009056	228 (nt)	TSA	Infectious hypodermal and hematopoietic necrosis virus	Penstyldensovirus	Densovirinae	AFA36168	8,00E-12	49%	89%	NS
Mollusca	Gastropoda	Neotaenioglossa	Calyptraeidae	Crepidula fornicata	x	586 (nt)	RNAseq	Seal parvovirus	Protoparvovirus	Parvovirinae	AHA86835	2,00E-07	39%	44%	NS
Nematoda	Secernentea	Ascaridida	Ascarididae	Ascaris suum	JI174103	1289 (nt)	TSA	Dendrolimus punctatus densovirus	Iteradensovirus	Densovirinae	YP_164339	1,00E-15	27%	42%	NS
Platyhelminthes	Cestoda	Cyclophyllidea	Anoplocephalidae	Moniezia expansa	JL291017	1482 (nt)	TSA	Infectious hypodermal and hematopoietic necrosis virus	Penstyldensovirus	Densovirinae	AFA36168	2,00E-18	42%	24%	NS
Platyhelminthes	Cestoda	Cyclophyllidea	Hymenolepididae	Hymenolepis diminua	LM389152.1	3287 (nt)	nr	Decapod penstyldensovirus 1	Penstyldensovirus	Densovirinae	AFA36168.1	5,00E-22	26%	31%	NS
					LM387022.1	8913 (nt)	nr	Decapod penstyldensovirus 1	Penstyldensovirus	Densovirinae	ALE60543.1	1,00E-10	32%	12%	NS
					LM387577.1	6568 (nt)	nr	Decapod penstyldensovirus 1	Penstyldensovirus	Densovirinae	CAI58631.1	7,00E-10	35%	4%	NS
					LM388960.1	3553 (nt)	nr	Decapod penstyldensovirus 1	Penstyldensovirus	Densovirinae	AFA36168.1	3,00E-22	25%	30%	NS
					LM389526.1	2886 (nt)	nr	Decapod penstyldensovirus 1	Penstyldensovirus	Densovirinae	AFA36168.1	1,00E-23	30%	24%	NS
					LM389639.1	2778 (nt)	nr	Decapod penstyldensovirus 1	Penstyldensovirus	Densovirinae	AFA36168.1	1,00E-20	32%	19%	NS
					LM390089.1	2383 (nt)	nr	Decapod penstyldensovirus 1	Penstyldensovirus	Densovirinae	AFA36168.1	6,00E-25	26%	44%	NS
					LM390786.1	1902 (nt)	nr	Decapod penstyldensovirus 1	Penstyldensovirus	Densovirinae	AFA36168.1	3,00E-24	26%	58%	NS
					LM391469.1	1566 (nt)	nr	Decapod penstyldensovirus 1	Penstyldensovirus	Densovirinae	AFA36168.1	2,00E-25	29%	50%	NS
					LM391906.1	1401 (nt)	nr	Decapod penstyldensovirus 1	Penstyldensovirus	Densovirinae	AFA36168.1	3,00E-21	32%	37%	NS
					LM393614.1	928 (nt)	nr	Decapod penstyldensovirus 1	Penstyldensovirus	Densovirinae	AFA36168.1	7,00E-19	30%	70%	NS
					LM395168.1	697 (nt)	nr	Decapod penstyldensovirus 1	Penstyldensovirus	Densovirinae	CDS34279.1	3,00E-21	34%	73%	NS
					LM394712.1	755 (nt)	nr	Decapod penstyldensovirus 1	Penstyldensovirus	Densovirinae	ALE60543.1	8,00E-13	32%	51%	NS
					LM389664.1	2747 (nt)	nr	Decapod penstyldensovirus 1	Penstyldensovirus	Densovirinae	CAI58631.1	1,00E-09	33%	10%	NS
Platyhelminthes	Cestoda	Cyclophyllidea	Hymenolepididae	Hymenolepis microstoma	CDJ12116	569 (aa)	nr	Infectious hypodermal and hematopoietic necrosis virus	Penstyldensovirus	Densovirinae	AFA36168	9,00E-26	35%	55%	NS
					CDI96179	495 (aa)	nr	Infectious hypodermal and hematopoietic necrosis virus	Penstyldensovirus	Densovirinae	AFA36168	1,00E-18	34%	31%	NS
					CDJ13943	339 (aa)	nr	Infectious hypodermal and hematopoietic necrosis virus	Penstyldensovirus	Densovirinae	AFA36168	7,00E-22	30%	53%	NS
					CDJ15564	218 (aa)	nr	Infectious hypodermal and hematopoietic necrosis virus	Penstyldensovirus	Densovirinae	AFA36168	2,00E-21	34%	70%	NS
					CDJ12117	195 (aa)	nr	Infectious hypodermal and hematopoietic necrosis virus	Penstyldensovirus	Densovirinae	AFA36168	2,00E-11	36%	45%	NS
					CDJ12115	162 (aa)	nr	Infectious hypodermal and hematopoietic necrosis virus	Penstyldensovirus	Densovirinae	AFA36168	2,00E-09	38%	44%	NS
					CDJ14557	152 (aa)	nr	Infectious hypodermal and hematopoietic necrosis virus	Penstyldensovirus	Densovirinae	AFA36168	1,00E-10	35%	78%	NS
					CDJ11897	151 (aa)	nr	Infectious hypodermal and hematopoietic necrosis virus	Penstyldensovirus	Densovirinae	AFA36168	2,00E-12	39%	62%	NS
					CDJ13946	146 (aa)	nr	Infectious hypodermal and hematopoietic necrosis virus	Penstyldensovirus	Densovirinae	AFA36168	2,00E-14	32%	80%	NS
					CDI96352	139 (aa)	nr	Infectious hypodermal and hematopoietic necrosis virus	Penstyldensovirus	Densovirinae	AFA36168	6,00E-09	39%	57%	NS
					CDJ13509	85 (aa)	nr	Infectious hypodermal and hematopoietic necrosis virus	Penstyldensovirus	Densovirinae	AFA36168	1,00E-08	33%	100%	NS
					CDS35108.1	495 (aa)	nr	Decapod penstyldensovirus 1	Penstyldensovirus	Densovirinae	AII19291.1	2,00E-18	34%	31%	NS
					CDS34279.1	218 (aa)	nr	Decapod penstyldensovirus 1	Penstyldensovirus	Densovirinae	AFA36168.1	5,00E-21	34%	70%	NS
					CDS30831.1	569 (aa)	nr	Decapod penstyldensovirus 1	Penstyldensovirus	Densovirinae	AFA36168.1	2,00E-25	35%	31%	NS
					CDS33272.1	152 (aa)	nr	Decapod penstyldensovirus 1	Penstyldensovirus	Densovirinae	AFA36168.1	2,00E-10	35%	78%	NS
					CDS30612.1	151 (aa)	nr	Decapod penstyldensovirus 1	Penstyldensovirus	Densovirinae	ALE60551.1	2,00E-12	40%	62%	NS
					CDS30832.1	195 (aa)	nr	Decapod penstyldensovirus 1	Penstyldensovirus	Densovirinae	AFA36168.1	4,00E-11	36%	45%	NS
					CDS32658.1	339 (aa)	nr	Aedes albopictus C6/36 cell densovirus	Brevidensovirus	Densovirinae	AAM28943.1	5,00E-15	27%	78%	NS
					CDS30830.1	162 (aa)	nr	Decapod penstyldensovirus 1	Penstyldensovirus	Densovirinae	AFA36168.1	3,00E-09	38%	44%	NS
					CDS32661.1	146 (aa)	nr	Turkey parvovirus TP1-2012/HUN	Aveparvovirus	Parvovirinae	AHF54687.1	1,00E-09	36%	78%	NS
					CDS26228.1	118 (aa)	nr	Decapod penstyldensovirus 1	Penstyldensovirus	Densovirinae	AFA36168.1	2,00E-25	29%	50%	NS
					CDS26229.1	166 (aa)	nr	Decapod penstyldensovirus 1	Penstyldensovirus	Densovirinae	AFA36168.1	3,00E-21	32%	37%	NS
					CDS27819.1	196 (aa)	nr	Decapod penstyldensovirus 1	Penstyldensovirus	Densovirinae	AFA36168.1	7,00E-19	30%	70%	NS
					CDS29136.1	316 (aa)	nr	Decapod penstyldensovirus 1	Penstyldensovirus	Densovirinae	ALE60543.1	3,00E-21	34%	73%	NS
					CDS33099.1	397 (aa)	nr	Decapod penstyldensovirus 1	Penstyldensovirus	Densovirinae	AII19291.1	2,00E-32	28%	93%	NS
					CDS33100.1	293 (aa)	nr	Decapod penstyldensovirus 1	Penstyldensovirus	Densovirinae	ABR23509.1	4,00E-28	29%	71%	NS
					CDS25721.1	163 (aa)	nr	Decapod penstyldensovirus 1	Penstyldensovirus	Densovirinae	ABR23509.1	4,00E-34	28%	80%	NS
					CDS26524.1	83 (aa)	nr	Decapod penstyldensovirus 1	Penstyldensovirus	Densovirinae	AFA36168.1	5,00E-25	43%	88%	NS
					CDS28210.1	157 (aa)	nr	Decapod penstyldensovirus 1	Penstyldensovirus	Densovirinae	AFA36168.1	2,00E-19	27%	75%	NS
					CDS29534.1	113 (aa)	nr	Decapod penstyldensovirus 1	Penstyldensovirus	Densovirinae	AFA36168.1	3,00E-19	29%	100%	NS
Platyhelminthes	Cestoda	Cyclophyllidea	Hymenolepididae	Hymenolepis nana	LM406075.1	4839 (nt)	nr	Decapod penstyldensovirus 1	Penstyldensovirus	Densovirinae	CAI58631.1	2,00E-16	40%	6%	NS
					LM409198.1	2518 (nt)	nr	Decapod penstyldensovirus 1	Penstyldensovirus	Densovirinae	CAI58631.1	2,00E-16	39%	11%	NS
Platyhelminthes	Cestoda	Cyclophyllidea	Taeniidae	Echinococcus granulosus	EUB54832	216 (aa)	nr	Infectious hypodermal and hematopoietic necrosis virus	Penstyldensovirus	Densovirinae	AGK30043	1,00E-24	35%	73%	NS
					W6UKP4	195 (aa)	UNIPROT	Penaeus stylirostris densovirus	Penstyldensovirus	Densovirinae	KM593913.1	8,00E-12	35%	68%	NS
					CDS25081.1	240 (aa)	nr	Decapod penstyldensovirus 1	Penstyldensovirus	Densovirinae	AYS1527.1	1,00E-27	26%	95%	NS
Platyhelminthes	Cestoda	Cyclophyllidea	Taeniidae	Echinococcus multilocularis	U6HDP5	431 (aa)	UNIPROT	Infectious hypodermal and hematopoietic necrosis virus	Penstyldensovirus	Densovirinae	AAG10007	1,00E-25	26%	95%	NS
					U6HF69	431 (aa)	UNIPROT	Infectious hypodermal and hematopoietic necrosis virus	Penstyldensovirus	Densovirinae	AAG10007	1,00E-25	26%	95%	NS
					CDI70288	431 (aa)	nr	Infectious hypodermal and hematopoietic necrosis virus	Penstyldensovirus	Densovirinae	ABR23509	7,00E-28	26%	95%	NS
					U6HGG3	431 (aa)	UNIPROT	Infectious hypodermal and hematopoietic necrosis virus	Penstyldensovirus	Densovirinae	AFA36168	1,00E-27	26%	95%	NS
					U6FUW9	431 (aa)	UNIPROT	Infectious hypodermal and hematopoietic necrosis virus	Penstyldensovirus	Densovirinae	AAG10007	2,00E-26	26%	95%	NS
					U6H8G0	404 (aa)	UNIPROT	Infectious hypodermal and hematopoietic necrosis virus	Penstyldensovirus	Densovirinae	AAG10007	1,00E-25	26%	96%	NS
					CDI70553	394 (aa)	nr	Infectious hypodermal and hematopoietic necrosis virus	Penstyldensovirus	Densovirinae	ADB12609	4,00E-22	32%	46%	NS
					U6HA22	377 (aa)	UNIPROT	Infectious hypodermal and hematopoietic necrosis virus	Penstyldensovirus	Densovirinae	AFA36168	1,00E-25	33%	48%	NS
					CDI70496	376 (aa)	nr	Infectious hypodermal and hematopoietic necrosis virus	Penstyldensovirus	Densovirinae	AFA36168	7,00E-16	28%	44%	NS
					CDI70555	371 (aa)	nr	Infectious hypodermal and hematopoietic necrosis virus	Penstyldensovirus	Densovirinae	AAG10007	1,00E-16	26%	90%	NS
					U6HLE6	392 (aa)	UNIPROT	Infectious hypodermal and hematopoietic necrosis virus	Penstyldensovirus	Densovirinae	AFA36168	7,00E-25	24%	97%	NS
					U6H9K2	343 (aa)	UNIPROT	Infectious hypodermal and hematopoietic necrosis virus	Penstyldensovirus	Densovirinae	AFA36168	2,00E-24	32%	53%	NS
					U6H9L2	313 (aa)	UNIPROT	Aedes aegypti densovirus	Brevidensovirus	Densovirinae	YP_002854229	2,00E-10	22%	97%	NS
					U6HNK1	303 (aa)	UNIPROT	Infectious hypodermal and hematopoietic necrosis virus	Penstyldensovirus	Densovirinae	AFA36168	2,00E-24	32%	60%	NS
					U6HA06	272 (aa)	UNIPROT	Infectious hypodermal and hematopoietic necrosis virus	Penstyldensovirus	Densovirinae	AFA36168	6,00E-24	32%	67%	NS
					U6HHY9	272 (aa)	UNIPROT	Infectious hypodermal and hematopoietic necrosis virus	Penstyldensovirus	Densovirinae	AFA36168	5,00E-25	32%	67%	NS
					U6HC26	272 (aa)	UNIPROT	Infectious hypodermal and hematopoietic necrosis virus	Penstyldensovirus	Densovirinae	AFA36168	6,00E-25	32%	67%	NS
					U6H8N8	251 (aa)	UNIPROT	Infectious hypodermal and hematopoietic necrosis virus	Penstyldensovirus	Densovirinae	AFA36168	2,00E-24	32%	72%	NS
					CDI70556	247 (aa)	UNIPROT	Infectious hypodermal and hematopoietic necrosis virus	Penstyldensovirus	Densovirinae	AFA36168	4,00E-24	32%	74%	NS
					U6H9Z7	184 (aa)	UNIPROT	Infectious hypodermal and hematopoietic necrosis virus	Penstyldensovirus	Densovirinae	AFA36168	5,00E-23	33%	95%	NS
					U6HD97	184 (aa)	UNIPROT	Infectious hypodermal and hematopoietic necrosis virus	Penstyldensovirus	Densovirinae	AFA36168	2,00E-22	33%	95%	NS
					U6H9K6	184 (aa)	UNIPROT	Infectious hypodermal and hematopoietic necrosis virus	Penstyldensovirus	Densovirinae	AFA36168	3,00E-22	33%	95%	NS
					U6H948	174 (aa)	UNIPROT	Infectious hypodermal and hematopoietic necrosis virus	Penstyldensovirus	Densovirinae	AFA36168	9,00E-22	34%	90%	NS
					U6H9C9	165 (aa)	UNIPROT	Infectious hypodermal and hematopoietic necrosis virus	Penstyldensovirus	Densovirinae	AFA36168	6,00E-21	34%	91%	NS
					U6HA02	169 (aa)	UNIPROT	Infectious hypodermal and hematopoietic necrosis virus	Penstyldensovirus	Densovirinae	AFA36168	4,00E-20	34%	89%	NS
					CDI70562	118 (aa)	nr	Infectious hypodermal and hematopoietic necrosis virus	Penstyldensovirus	Densovirinae	AFA36168	1,00E-10	29%	90%	NS
					U6HFK7	107 (aa)	UNIPROT	Infectious hypodermal and hematopoietic necrosis virus	Penstyldensovirus	Densovirinae	AAG10007	6,00E-05	28%	90%	NS
					A0A087VWP3	377 (aa)	UNIPROT	Penaeus stylirostris densovirus	Penstyldensovirus	Densovirinae	AID76764.1	5,00E-26	34%	48%	NS
					A0A068XWU9	392 (aa)	UNIPROT	Penaeus stylirostris densovirus	Penstyldensovirus	Densovirinae	AID76764.1	4,00E-25	34%	46%	NS
					A0A077RCT5	272 (aa)	UNIPROT	Penaeus stylirostris densovirus	Penstyldensovirus	Densovirinae	AID76764.1	3,00E-25	33%	66%	NS
					CDS35386.1	431 (aa)	nr	Decapod penstyldensovirus 1	Penstyldensovirus	Densovirinae	AYS1527.1	1,00E-27	26%	95%	NS
					CDS35653.1	371 (aa)	nr	Decapod penstyldensovirus 1	Penstyldensovirus	Densovirinae	ALE60551.1	2,00E-16	26%	94%	NS
					CDS35660.1	431 (aa)	nr	Decapod penstyldensovirus 1	Penstyldensovirus	Densovirinae	AAG10007.2	3,00E-26	26%	95%	NS
					CDS35651.1	394 (aa)	nr	Decapod penstyldensovirus 1	Penstyldensovirus	Densovirinae	AFA36168.1	8,00E-23	32%	46%	NS
					CDS36723.1	392 (aa)	nr	Decapod penstyldensovirus 1	Penstyldensovirus	Densovirinae	AFA36168.1	1,00E-24	24%	97%	NS
					CDS35594.1	376 (aa)	nr	Decapod penstyldensovirus 1	Penstyldensovirus	Densovirinae	AFA36168.1	1,00E-15	28%	44%	NS
					CDS36692.1	303 (aa)	nr	Decapod penstyldensovirus 1	Penstyldensovirus	Densovirinae	AFA36168.1	4,00E-24	32%	60%	NS
					CDS36816.1	272 (aa)	nr	Decapod penstyldensovirus 1	Penstyldensovirus	Densovirinae	AFA36168.1	1,00E-24	32%	67%	NS
					CDS36689.1	247 (aa)	nr	Decapod penstyldensovirus 1	Penstyldensovirus	Densovirinae	AFA36168.1	9,00E-24	32%	74%	NS
Platyhelminthes	Cestoda	Cyclophyllidea	Taeniidae	Hydatigera taeniaeformis	LL723621.1	3111 (nt)	nr	Decapod penstyldensovirus 1	Penstyldensovirus	Densovirinae	AFA36168.1	1,00E-08	30%	10%	NS
					LL725419.1	2418 (nt)	nr	Decapod penstyldensovirus 1	Penstyldensovirus	Densovirinae	AFA36168.1	2,00E-18	34%	18%	NS
Platyhelminthes	Cestoda	Cyclophyllidea	Taeniidae	Taenia asiatica	LM127006.1	12071 (nt)	nr	Penaeus stylirostris densovirus	Penstyldensovirus	Densovirinae	AID76764.1	7,00E-18	29%	5%	NS
Platyhelminthes	Cestoda	Cyclophyllidea	Taeniidae	Taenia multiceps	JR938527.1	452 (nt)	TSA	Decapod penstyldensovirus 1	Penstyldensovirus	Densovirinae	CAI58631.1	1,00E-07	38%	53%	NS
					JR929085	439 (nt)	TSA	Infectious hypodermal and hematopoietic necrosis virus	Penstyldensovirus	Densovirinae	AAG10007	1,00E-06	38%	65%	NS
Platyhelminthes	Monogenea	Monopisthocotylea	Capsalidae	Neobenedenia melleni	GW918566	1014 (nt)	EST	Infectious hypodermal and hematopoietic necrosis virus	Penstyldensovirus	Densovirinae	AFA36168	3,00E-06	31%	37%	NS
Platyhelminthes	Trematoda	Echinostomida	Echinostomatidae	Echinostoma caproni	LL274983.1	3576 (nt)	nr	Decapod penstyldensovirus 1	Penstyldensovirus	Densovirinae	AEV46269.1	6,00E-35	41%	31%	NS
					LL284665.1	1785 (nt)	nr	Decapod penstyldensovirus 1	Penstyldensovirus	Densovirinae	AII19291.1	3,00E-32	41%	63%	NS
					LL285499.1	1709 (nt)	nr	Decapod penstyldensovirus 1	Penstyldensovirus	Densovirinae	AII19291.1	8,00E-35	42%	65%	NS
					LL286487.1	1623 (nt)	nr	Decapod penstyldensovirus 1	Penstyldensovirus	Densovirinae	AII19291.1	2,00E-32	42%	70%	NS
					LL259483.1	8082 (nt)	nr	Decapod penstyldensovirus 1	Penstyldensovirus	Densovirinae	AFA36168.1	3,00E-16	29%	9%	NS
Platyhelminthes	Trematoda	Opisthorchiida	Opisthorchiidae	Clonorchis sinensis	GAA57639	928 (aa)	nr	Infectious hypodermal and hematopoietic necrosis virus	Penstyldensovirus	Densovirinae	AEV46269	2,00E-29	31%	32%	NS
					GAA57641	511 (aa)	nr	Infectious hypodermal and hematopoietic necrosis virus	Penstyldensovirus	Densovirinae	AFA36168	8,00E-36	29%	72%	NS
					GAA32750	500 (aa)	nr	Infectious hypodermal and hematopoietic necrosis virus	Penstyldensovirus	Densovirinae	AFA36168	4,00E-29	33%	43%	NS
					GAA57954	496 (aa)	nr	Infectious hypodermal and hematopoietic necrosis virus	Penstyldensovirus	Densovirinae	AAM93906	1,00E-37	29%	79%	NS
					GAA40146	495 (aa)	nr	Infectious hypodermal and hematopoietic necrosis virus	Penstyldensovirus	Densovirinae	AAM93906	8,00E-38	29%	77%	NS
					GAA56553	420 (aa)	nr	Infectious hypodermal and hematopoietic necrosis virus	Penstyldensovirus	Densovirinae	AGW23423	1,00E-26	30%	76%	NS
					GAA57952	317 (aa)	nr	Infectious hypodermal and hematopoietic necrosis virus	Penstyldensovirus	Densovirinae	AAM93906	1,00E-10	25%	88%	NS
					GAA57638	273 (aa)	nr	Infectious hypodermal and hematopoietic necrosis virus	Penstyldensovirus	Densovirinae	AFA36168	2,00E-36	34%	94%	NS
					GAA57640	249 (aa)	nr	Infectious hypodermal and hematopoietic necrosis virus	Penstyldensovirus	Densovirinae	AFX58966	1,00E-26	31%	95%	NS
Platyhelminthes	Trematoda	Opisthorchiida	Opisthorchiidae	Opisthorchis felineus	GBJA01005901.1	2230 (nt)	TSA	Decapod penstyldensovirus 1	Penstyldensovirus	Densovirinae	AFA36168.1	2,00E-20	31%	30%	NS
					GBJA01002120.1	1210 (nt)	TSA	Decapod penstyldensovirus 1	Penstyldensovirus	Densovirinae	AFA36168.1	9,00E-32	30%	65%	NS
					GBJA01008850.1	1509 (nt)	TSA	Decapod penstyldensovirus 1	Penstyldensovirus	Densovirinae	AII19291.1	5,00E-37	28%	80%	NS
					GBJA01006694.1	1706 (nt)	TSA	Decapod penstyldensovirus 1	Penstyldensovirus	Densovirinae	AFR11649.1	1,00E-30	27%	68%	NS
					GBJA01002122.1	894 (nt)	TSA	Decapod penstyldensovirus 1	Penstyldensovirus	Densovirinae	AFA36168.1	3,00E-21	40%	50%	NS
					GBJA01002124.1	1551 (nt)	TSA	Decapod penstyldensovirus 1	Penstyldensovirus	Densovirinae	AII19291.1	1,00E-35	28%	72%	NS
					GBJA01010281.1	478 (nt)	TSA	Decapod penstyldensovirus 1	Penstyldensovirus	Densovirinae	AFA36168.1	5,00E-20	38%	74%	NS
					GBJA01002117.1	692 (nt)	TSA	Decapod penstyldensovirus 1	Penstyldensovirus	Densovirinae	AII19291.1	3,00E-08	26%	80%	NS
					GBJA01010476.1	884 (nt)	TSA	Decapod penstyldensovirus 1	Penstyldensovirus	Densovirinae	AFA36168.1	5,00E-08	40%	32%	NS
Platyhelminthes	Trematoda	Opisthorchiida	Opisthorchiidae	Opisthorchis viverrini	XM_009172370.1	792 (nt)	nr	Decapod penstyldensovirus 1	Penstyldensovirus	Densovirinae	AFA36168.1	2,00E-20	32%	86%	NS
					XM_009178560.1	1533 (nt)	nr	Decapod penstyldensovirus 1	Penstyldensovirus	Densovirinae	ABR23509.1	4,00E-28	29%	71%	NS
					XM_009172391.1	2220 (nt)	nr	Decapod penstyldensovirus 1	Penstyldensovirus	Densovirinae	AAR15379.1	4,00E-09	24%	37%	NS
					XM_009179278.1	1738 (nt)	nr	Decapod penstyldensovirus 1	Penstyldensovirus	Densovirinae	ABR23509.1	4,00E-34	28%	80%	NS
					XM_009175397.1	1185 (nt)	nr	Decapod penstyldensovirus 1	Penstyldensovirus	Densovirinae	AII19291.1	2,00E-32	28%	93%	NS
					XM_009179534.1	420 (nt)	nr	Decapod penstyldensovirus 1	Penstyldensovirus	Densovirinae	AFA36168.1	5,00E-25	43%	88%	NS
					XM_009179438.1	902 (nt)	nr	Decapod penstyldensovirus 1	Penstyldensovirus	Densovirinae	AFA36168.1	1,00E-30	31%	82%	NS
					XM_009179266.1	2535 (nt)	nr	Decapod penstyldensovirus 1	Penstyldensovirus	Densovirinae	AFA36168.1	2,00E-26	30%	30%	NS
					XM_009179678.1	594 (nt)	nr	Decapod penstyldensovirus 1	Penstyldensovirus	Densovirinae	AFA36168.1	3,00E-19	29%	100%	NS
					XP_009170634	263 (aa)	nr	Decapod penstyldensovirus 1	Penstyldensovirus	Densovirinae	AFA36168	2,00E-29	32%	87%	NS
					XP_009176824	510 (aa)	nr	Decapod penstyldensovirus 1	Penstyldensovirus	Densovirinae	ABR23509	2,00E-36	29%	71%	NS
					XP_009177702	300 (aa)	nr	Decapod penstyldensovirus 1	Penstyldensovirus	Densovirinae	AFA36168	8,00E-31	31%	83%	NS
					XP_009177942	198 (aa)	nr	Penaeus monodon penstyldensovirus 2	Penstyldensovirus	Densovirinae	AAM94165	1,00E-19	30%	100%	NS
					XP_009177808	278 (aa)	nr	Decapod penstyldensovirus 1	Penstyldensovirus	Densovirinae	AFA36168	5,00E-20	27%	80%	NS
					XP_009170655	740 (aa)	nr	Decapod penstyldensovirus 1	Penstyldensovirus	Densovirinae	AAR15379	3,00E-09	24%	38%	NS
					XP_009177798	140 (aa)	nr	Decapod penstyldensovirus 1	Penstyldensovirus	Densovirinae	AFA36168	3,00E-25	43%	88%	NS
					XP_009177971	72 (aa)	nr	Decapod penstyldensovirus 1	Penstyldensovirus	Densovirinae	AFA36168	2,00E-14	49%	94%	NS
					XP_009178033	184 (aa)	nr	Decapod penstyldensovirus 1	Penstyldensovirus	Densovirinae	AII19291	9,00E-05	24%	94%	NS
					XP_009177542	579 (aa)	nr	Decapod penstyldensovirus 1	Penstyldensovirus	Densovirinae	ABR23509	2,00E-34	28%	80%	NS
					XP_009177530	844 (aa)	nr	Decapod penstyldensovirus 1	Penstyldensovirus	Densovirinae	AFA36168	2,00E-26	30%	30%	NS
					XP_009173661	394 (aa)	nr	Penaeus monodon penstyldensovirus 2	Penstyldensovirus	Densovirinae	AAM93906	2,00E-32	28%	89%	NS
Platyhelminthes	Trematoda	Plagiorchiida	Dicrocoeliidae	Dicrocoelium dendriticum	LK428994.1	2134 (nt)	nr	Decapod penstyldensovirus 1	Penstyldensovirus	Densovirinae	AGK30043.1	4,00E-32	26%	56%	NS
					LK446595.1	1838 (nt)	nr	Decapod penstyldensovirus 1	Penstyldensovirus	Densovirinae	AFA36168.1	3,00E-22	41%	20%	NS
					LK459200.1	1697 (nt)	nr	Decapod penstyldensovirus 1	Penstyldensovirus	Densovirinae	AFA36168.1	5,00E-25	33%	33%	NS
					LK595374.1	994 (nt)	nr	Decapod penstyldensovirus 1	Penstyldensovirus	Densovirinae	AFA36168.1	4,00E-06	38%	23%	NS
Platyhelminthes	Trematoda	Strigeatida	Schistosomatidae	Schistosoma mansoni	XM_002571303	3204 (nt)	nr	Decapod penstyldensovirus 1	Penstyldensovirus	Densovirinae	AFA36168	[illegible]	[illegible]	[illegible]	NS
					XP_002571349	1067 (aa)	nr	Infectious hypodermal and hematopoietic necrosis virus	Penstyldensovirus	Densovirinae	AFA36168	2,00E-33	29%	33%	NS
					CCD58408	362 (aa)	nr	Infectious hypodermal and hematopoietic necrosis virus	Penstyldensovirus	Densovirinae	AFA36168	2,00E-13	26%	74%	NS
Platyhelminthes	Trematoda	Strigeatida	Schistosomatidae	Schistosoma margrebowiei	LL888002.1	8085 (nt)	nr	Decapod penstyldensovirus 1	Penstyldensovirus	Densovirinae	ALE60543.1	5,00E-32	30%	14%	NS
Platyhelminthes	Trematoda	Strigeatida	Schistosomatidae	Schistosoma mattheei	LM181082.1	2250 (nt)	nr	Decapod penstyldensovirus 1	Penstyldensovirus	Densovirinae	AFA36168.1	7,00E-27	37%	10%	NS
Platyhelminthes	Turbellaria	Tricladida	Dugesiidae	Schmidtea mediterranea	GAKN01010353	660 (nt)	TSA	Snake parvovirus 1	Dependoparvovirus	Parvovirinae	YP_068093	1,00E-06	32%	65%	NS
Urochordata	Ascidiacea	Enterogona	Cionidae	Ciona intestinalis	BW334595	572 (nt)	EST	Minute virus of mice	Protoparvovirus	Parvovirinae	CAA24313	4,00E-15	39%	66%	VP
Chordata	Actinopterygii	Cyprinodontiformes	Fundulidae	Fundulus grandis	JW661434	3397 (nt)	TSA	Eidolon helvum parvovirus 2	Tetraparvovirus	Parvovirinae	AGL97807	1,00E-44	31%	28%	NS
					JW661431	3102 (nt)	TSA	Eidolon helvum parvovirus 2	Tetraparvovirus	Parvovirinae	AGL97807	2,00E-39	37%	21%	NS
Chordata	Amphibia	Anura	Pipidae	Xenopus tropicalis	CF784470.1	874 (nt)	EST	Adeno-associated virus 2	Dependoparvovirus	Parvovirinae	AF043303.1	3,00E-24	100%	6%	NS
Chordata	Amphibia	Caudata	Hynobiidae	Hynobius chinensis	GAQK01079305.1	207 (nt)	TSA	Human parvovirus B19	Erythroparvovirus	Parvovirinae	KM393169.1	2,00E-04	85%	25%	NS
Chordata	Amphibia	Caudata	Hynobiidae	Hynobius retardatus	LB005884.1	403 (nt)	TSA	Human parvovirus B19	Erythroparvovirus	Parvovirinae	M24682.1	3,00E-07	90%	12%	NS
Chordata	Aves	Galliformes	Phasianidae	Gallus gallus	AM071684	639 (nt)	EST	Turkey parvovirus 1078	Aveparvovirus	Parvovirinae	ACA28962	1,00E-127	100%	85%	NS
Chordata	Mammalia	Artiodactyla	Bovidae	Bos taurus	CO892248	264 (nt)	EST	Adeno-associated virus - 2	Dependoparvovirus	Parvovirinae	YP_680428	2,00E-31	91%	98%	VP
					CO888893	195 (nt)	EST	Adeno-associated virus - 2	Dependoparvovirus	Parvovirinae	YP_680428	3,00E-32	94%	98%	VP
Chordata	Mammalia	Artiodactyla	Bovidae	Capra hircus	GAOD01023083	552 (nt)	TSA	Adeno-associated virus	Dependoparvovirus	Parvovirinae	AAS99238	3,00E-06	58%	28%	VP
					GAOD01006658	1197 (nt)	TSA	Porcine parvovirus 4	Copiparvovirus	Parvovirinae	AGM20648	5,00E-58	35%	98%	VP
Chordata	Mammalia	Artiodactyla	Suidae	Sus scrofa	EV965154	287 (nt)	EST	Eidolon helvum parvovirus 2	Tetraparvovirus	Parvovirinae	AGL97807	2,00E-33	62%	96%	NS
Chordata	Mammalia	Diprotodontia	Macropodidae	Macropus eugenii	FY713038	729 (nt)	EST	Feline panleukopenia virus	Protoparvovirus	Parvovirinae	P04864	6,00E-33	47%	66%	VP
Chordata	Mammalia	Diprotodontia	Phalangeridae	Trichosurus vulpecula	DY609048	916 (nt)	EST	Mouse parvovirus 5a	Protoparvovirus	Parvovirinae	YP_002332921	2,00E-10	40%	26%	VP
					EC348320	777 (nt)	EST	Bufavirus-3	Protoparvovirus	Parvovirinae	YP_009058894	6,00E-64	49%	96%	NS
Chordata	Mammalia	Lagomorpha	Leporidae	Oryctolagus cuniculus	XM_008261525.1	3660 (nt)	nr	Adeno-associated virus - 8	Dependoparvovirus	Parvovirinae	YP_077179.1	3,00E-97	40%	40%	NS
					GBCK01348906	3071 (nt)	TSA	Adeno-associated virus - 2	Dependoparvovirus	Parvovirinae	YP_680422	8,00E-83	49%	28%	NS-VP
					GBCI01152991	1263 (nt)	TSA	Adeno-associated virus - 5	Dependoparvovirus	Parvovirinae	YP_068409	1,00E-40	40%	91%	VP
					GBCI01025461	201 (nt)	TSA	Adeno-associated virus	Dependoparvovirus	Parvovirinae	AAS99314	4,00E-17	59%	95%	VP
					GBCI01079387	415 (nt)	TSA	Adeno-associated virus 13	Dependoparvovirus	Parvovirinae	ABZ10812	4,00E-23	51%	79%	VP
					GBCK01194261	205 (nt)	TSA	Adeno-associated virus - 2	Dependoparvovirus	Parvovirinae	1U0J_A	2,00E-18	60%	92%	NS
					XP_008259747	361 (aa)	nr	Adeno-associated virus 12	Dependoparvovirus	Parvovirinae	ABI16638	4,00E-94	47%	92%	NS
Chordata	Mammalia	Primates	Hominidae	Homo sapiens	Z30129	333 (nt)	EST	Canine parvovirus 2a	Protoparvovirus	Parvovirinae	AFL55672	2,00E-63	95%	89%	VP2
Chordata	Mammalia	Rodentia	Chinchillidae	Chinchilla lanigera	GBDF01076442	595 (nt)	TSA	Adeno-associated virus - 7	Dependoparvovirus	Parvovirinae	YP_077177	2,00E-06	31%	46%	NS
					GBDF01076446.1	280 (nt)	TSA	Adeno-associated virus -2	Dependoparvovirus	Parvovirinae	AYE95375.1	1,00E-10	74%	47%	NS
					GBDF01269873	806 (nt)	TSA	Bovine adeno-associated virus	Dependoparvovirus	Parvovirinae	YP_024970	2,00E-55	51%	70%	NS
					GBDF01150884	850 (nt)	TSA	Bovine adeno-associated virus	Dependoparvovirus	Parvovirinae	YP_024970	3,00E-119	61%	94%	NS
Chordata	Mammalia	Rodentia	Octodontidae	Octodon degus	XM_004634678	1527 (nt)	nr	Adeno-associated virus - 4	Dependoparvovirus	Parvovirinae	NP_044926	0.0	63%	97%	NS
					XP_004634735	508 (aa)	nr	Adeno-associated virus - 4	Dependoparvovirus	Parvovirinae	NP_044926	0.0	63%	97%	NS
Chordata	Mammalia	Rodentia	Muridae	Rattus norvegicus	DW307647	340 (nt)	EST	Minute virus of mice	Protoparvovirus	Parvovirinae	CAA24313	3,00E-24	64%	74%	VP
					DW305306	335 (nt)	EST	Autonomous rat parvovirus RV-Y	Protoparvovirus	Parvovirinae	AAK27438	3,00E-41	96%	64%	NS
					DW318245	363 (nt)	EST	Minute virus of mice	Protoparvovirus	Parvovirinae	CAA24313	1,00E-14	75%	42%	VP
					DW316788	360 (nt)	EST	Porcine parvovirus	Protoparvovirus	Parvovirinae	AFV25457	2,00E-16	63%	51%	VP
					DW295519	318 (nt)	EST	Kilham rat virus	Protoparvovirus	Parvovirinae	AAB38325	7,00E-54	88%	90%	NS
					DW328103	407 (nt)	EST	Autonomous rat parvovirus RV-Y	Protoparvovirus	Parvovirinae	AAK27438	4,00E-32	90%	63%	NS
					DW397086	575 (nt)	EST	Kilham rat virus	Protoparvovirus	Parvovirinae	AAB38325	3,00E-31	87%	46%	NS
					DW327620	404 (nt)	EST	Autonomous rat parvovirus RV-Y	Protoparvovirus	Parvovirinae	AAK27439	2,00E-18	70%	44%	NS
					DW327790	404 (nt)	EST	Kilham rat virus	Protoparvovirus	Parvovirinae	AAM93273	3,00E-51	86%	77%	VP
Asterids	x	Asterales	Asteraceae	Karelinia caspia	GAN01103541	412 (nt)	TSA	Culex pipiens densovirus	Ambidensovirus	Densovirinae	YP_002887625	2,00E-15	48%	61%	NS
Magnoliophyta	Magnoliopsida	Urticales	Cannabinaceae	Humulus lupulus	GAAW01123813	304 (nt)	TSA	Dysaphis plantaginea densovirus	Ambidensovirus	Densovirinae	ACI01073	7,00E-27	53%	90%	NS
Magnoliophyta	Liliopsida	Cyperales	Poaceae	Oryza sativa	CX103603.1	616 (nt)	EST	Periplaneta fuliginosa densovirus	Ambidensovirus	Densovirinae	NP_051017.1	2,00E-141	99%	99%	VP
Apicomplexa	Conoidasida	Eugregarinorida	Gregarinidae	Gregarina niphandrodes	XM_011135496.1	888 (nt)	nr	Blattella germanica densovirus-like virus	Ambidensovirus	Densovirinae	AFC75861.1	0.0	100%	94%	VP
					XM_011135495.1	702 (nt)	nr	Blattella germanica densovirus-like virus	Ambidensovirus	Densovirinae	AFC75860.1	1,00E-110	97%	99%	VP
					A0A023AWI8	295 (aa)	UNIPROT	Blattella germanica densovirus-like virus	Ambidensovirus	Densovirinae	AFC75861.1	0.0	100%	100%	VP
					XP_011133798.1	295 (aa)	nr	Blattella germanica densovirus-like virus	Ambidensovirus	Densovirinae	AFC75861.1	0.0	100%	100%	VP
					XP_011133797.1	233 (aa)	nr	Blattella germanica densovirus-like virus	Ambidensovirus	Densovirinae	AFC75860.1	1,00E-146	97%	91%	VP

Sub-family	Genus	Virus species or variant	Accession number
Densovirinae	Ambidensovirus	Acheta domesticus densovirus	HQ827781
		Acheta domesticus mini ambidensovirus	KF275669
		Blattella germanica densovirus	AY189948
		Blattella germanica densovirus-like virus	JQ320376
		Culex pipiens densovirus	FJ810126
		Diatraea saccharalis densovirus	AF036333
		Dysaphis plantaginea densovirus	FJ040397
		Galleria mellonella densovirus	L32896
		Helicoverpa armigera densovirus	JQ894784
		Junonia coenia densovirus	KC883978
		Mythimna loreyi densovirus	AY461507
		Myzus persicae densovirus	AY148187
		Periplaneta fuliginosa densovirus	AF192260
		Planococcus citri densovirus	AY032882
		Pseudoplusia includens densovirus	JX645046
		Solenopsis invicta densovirus	KC991097
	Brevidensovirus	Aedes aegypti densovirus 1	M37899
		Aedes aegypti densovirus 2	FJ360744
		Aedes albopictus densovirus 1	AY095351
		Aedes albopictus densovirus 2	X74945
		Aedes albopictus densovirus 3	AY310877
		Anopheles gambiae densovirus	EU233812
		Culex pipiens pallens densovirus	EF579771
		Haemagogus equinus densovirus	AY605055
		Mosquito densovirus BR/07	GU452720
	Hepandensovirus	Fenneropenaeus chinensis hepandensovirus	JN082231
		Penaeus chinensis hepandensovirus	AY008257
		Penaeus merguiensis hepandensovirus	DQ458781
		Penaeus monodon hepandensovirus 1	DQ002873
		Penaeus monodon hepandensovirus 2	EU247528
		Penaeus monodon hepandensovirus 3	EU588991
		Penaeus monodon hepandensovirus 4	FJ410797
	Iteradensovirus	Bombyx mori densovirus	AY033435
		Casphalia extranea densovirus	AF375296
		Danaus plexippus plexippus densovirus	KF963252
		Dendrolimus punctatus densovirus	AY665654
		Helicoverpa armigera densovirus	HQ613271
		Hordeum marinum itera-like densovirus	KM576800
		Papilio polyxenes densovirus	JX110122
		Sibine fusca densovirus	JX020762
	Penstyldensovirus	Penaeus monodon penstyldensovirus 1	GQ411199
		Penaeus monodon penstyldensovirus 2	AY124937
		Penaeus stylirostris penstyldensovirus 1	AF273215
		Penaeus stylirostris penstyldensovirus 2	GQ475529
Parvovirinae	Amdoparvovirus	Aleutian mink disease virus	JN040434
		Gray fox amdovirus	JN202450
	Aveparvovirus	Turkey parvovirus	GU214706
	Bocaparvovirus	California sea lion bocavirus 1	JN420361
		Canine bocavirus 1	JN648103
		Canine minute virus	FJ214110
		Feline bocavirus	JQ692585
		Human bocavirus 1	JQ923422
		Human bocavirus 4	FJ973561
		Porcine bocavirus 1	HM053693
		Porcine bocavirus 3	JF429834
		Porcine bocavirus 5	HQ223038
	Copiparvovirus	Bovine parvovirus 2	AF406966
		Porcine parvovirus 4	GQ387499
	Dependoparvovirus	Adeno-associated virus 1	AF063497
		Adeno-associated virus 5	AF085716
		Bat adeno-associated virus	GU226971
		California sea lion adeno-associated virus 1	JN420372
		Goose parvovirus	U25749
	Erythroparvovirus	Human parvovirus B19	M13178
	Protoparvovirus	Bufavirus 1	JX027296
		Canine parvovirus	M19296
		Mouse parvovirus 1	U12469
		Mouse parvovirus 3	DQ196318
		Porcine parvovirus PT4	U44978
		Rat parvovirus NTU1	AF036710
	Tetraparvovirus	Bovine hokovirus	EU200669
		Eidolon helvum parvovirus 1	JQ037753
		Human parvovirus 4	AY622943
		Porcine hokovirus	EU200677

Figure S1

```
>variole_D5_NC_001611
IINDIQPLTKKNRELYEKTLSSCL-CGATKGCLTFFFGETATGKSLLKSAISDLFVETGQTILTVLDKGPNIANMHKRSVFCSELPDFACSGTKKIRCVIGRPCFSNKINNRN-----HATIIIDTNYPVFDRIDNALMR----RIAVVRFRTHFS
>Penaeus_monodon_hepandensovirus_3_EU588991
RPKTIPIVSQNKTVQWIQDFMDIMHGNLPKVNCMMLYGNSNSGKTQLIEALTGLVNT-AIMTNVGDGGTFHFSNITMSTIVVGNETKIRTQTIEQWKGLCGGENVTMPMKYKEHKTHMRKPVFLTNQHPLMDISHYDDRRAIENRSFMYKVELGSE
>Hymenolepis_microstoma_CDS29136
LIIQYFKYR-----GVVFEVGTELHEEWQQAQDMPPIQPHVTCDSELRRARTKSTIA----ERTSRHTKFTLDELKRSDIRQFEEIFSAFSVNEIWI--------NMDKKTTKVNKIAKQQIQSLNAERLQSEKQNSYLQNL--QMMKHDCTVKHP
>Hymenolepis_microstoma_CDS33099
LTKNLFEHDEIVSIEDQEPLINRADDENGRRSEAI---PSTSART-------------GLEQSS--SPSFL-----GH------DPYGAEEGQEIFPQQLG----DMETRTKGALSLR----VAAEDESMENILLGGISITPDQ-----NYHTVII
>Penaeus_monodon_hepandensovirus_2_EU247528
RPKTIPIVSQNKTVQWIQDFMDIMHGNLPKVNCMMLYGNSNSGKTQLIEALTGLVNT-AIMTNVGDGGTFHFSNITMSTIVVGNETKIRTQTIEQWKGLCGGENVTMPMKYKEHKTHMRKPVFLTNQHPLVDISHYDDRRAIENRSFMYKVELGSE
>Penaeus_merguiensis_hepandensovirus_DQ458781
RPKTIPIISKNKTVQWIQDFMDIMHGNLPKINCMMLYGNSNSGKTQLIDALTGLVNT-AVMTNVGDGGTFHFSNITMSTIVVGNETKIRTQTIEQWKGLCGGENVTMPMKYKEHKTHMRKPVFLTNQHPLVDISHYDDRRAIENRSFMYKVELGSE
>Penaeus_chinensis_hepandensovirus_AY008257
RPKTIPIISKNKTVQWIQDFMDIMHGNLPKINCMMLYGNSNSGKTQLIEALTGLVNT-AIMTNVGDGGTFHFSNITMSTIVVGNETKIRTQTIEQWKGLCGGENVTMPMKYKEHKTHMRKPVFLTNQHPLVDISHYDDRRAIENRSFMYKVELGKE
>Fenneropenaeus_chinensis_hepandensovirus_JN082231
RPKTIPIISKNKTVQWIQDFMDIMHGNLPKINCMMLYGNSNSGKTQLIEALTGLVNT-AIMTNVGDGGTFHFSNITMGTIVVGNETKIRTQTIEQWKGLCGGENVTMPMKYKEHKTHMRKPVFLTNQHPLVDISHYDDRRAIENRSFMYKVELGKE
>Penaeus_monodon_hepandensovirus_4__FJ410797
RPKTIPIISKNKTVQWIQDFMDIMHGNLPKINCMMLYGNSNSGKTQLIEALTGLVNT-AVMTNVGDGGTFHFSNITMSTIVVGNETKIRTQTIEQWKGLCGGENVTMPMKYKEHKTHMRKPVFLTNQHPLVEISNYDDRKAIENRCFMYKVELGSE
>Penaeus_monodon_hepandensovirus_1_DQ002873
RPKTIPIVSKNKTVQWIQDFMDIIHGNLPKINCMMLYGNSNSGKTQLIEALTGLINT-AIMTNVGDGGTFHFSNITMSTIVVGNETKIRTQTIEQWKGLCGGENITMPMKYKEHKTHMRKPVFLTNQHPLVEISNYDDRKAIENRCFMYKVELGSE
>Acheta_domesticus_mini_ambidensovirus__KF275669
IIEQLLMYQFESVSDFIHQLYYVLNKQNGKKNCIELIGPPSSYKSTFLHWVAEAFIAVGIINIVNKTAPFGFAPAVNKRVLIIDDYNFSSEYHETLLNILSGTTCNVNVKYKTNGYVTHTPVLMASNYTFTSTRFEH-------RMVTFYWKEYK-
>Acheta_domesticus_densovirus_HQ827781
FLNELILFQEEGVKELLMNIVAWFNLGWPKVNTVCIIGNHNCGKNYFWDAVCCLGLNVGFLGRVNKTNKFALQDCVHKRIVIGNEISLEDGAKEDMKKLCEGCPFNVSVKHQSDGIVARTPVCLISNN---------CIPIACDRMKVFYWRPCSY
>Planococcus_citri_densovirus_AY032882
FLDELLKFQEEIIKPFLSNLRNWFNREGPKMNALCVIGPPNSGKNYFFDAIIALANNVGHIGRVNKTNNFALQDVVNRRIVVGNEISMEEGAKEDFKKLCEGTAFNVRVKFKADCIFTKTPVLLISNNELDICWDPHFKDV---RLKTIRWNTAPL
>Solenopsis_invicta__densovirus_KC991097
FVDDLLRFQEENIVELLTNVRDWFNDGWPKMNALCVIGPPNSGKNYFWDMFCSVAYNVGHIGRVNKTNQFALQECYGRRLVVGNEVSMEDGAKEDFKKLCEGTAFNIRVKYQGDKIFTKAPVLLISNFELDICYDTHFKNV---RLHTIRWVKCPL
>Papilio_polyxenes_densovirus_JX110122
IVEELLDYQFDAKRRFITDLYEILEKKHQKTNTFQIVSPPSAGKNFFIETVLAFYWNTGVIQNFNRYHSFPLMEAVNRRVNYWDEPNFEPDATETLKKLFAGTALKASVKFQKEANVQKTPVIITANRKFTSEVWDD-------RIIKYEWYQCPM
>Casphalia_extranea_densovirus_AF375296
IVEELLDYQEEAKRKFITDLYEILEKKHQKTNTFQIVSPPSAGKNFFIETVLAFYWNTGVIQNFNRYNNFPLMEAVNRRVNYWDEPNFEPDATETLKKLFAGTALKATVKFQKEANVQKTPVIITANHKFTKEVWDD-------RIIKYYWYQCPK
>Sibine_fusca_densovirus_JX020762
IVEELLDFQEEAKRKFITDLYEILEKKHQKTNTFQIVSPPSAGKNFFIETVLAFYWNTGVIQNFNRYNNFPLMEAVNRRVNYWDEPNFEPDAVETLKKLFAGTALKATVKFQKEANVQKTPVIITANYKFTKEVWDD-------RIIKYYWYPCPK
>Hordeum_marinum_iteradensovirus_KM576800
IVEELLDFQEDAKRKFITDLYEILEKKHQKTNTFQIVSPPSAGKNFFIETVLAFYWNTGVIQNFNRYNNFPLMEAVNRRVNYWDEPNFEPDATETLKKLFAGTALKATVKFQKEANVQKTPVIITANFKFTKEVWDD-------RIIKYYWYSCPK
>Bombyx_mori_densovirus_AY033435
IVEELLDFQEDAKKRFITDLYEILEKKHQKTNTFQIVSPPSAGKNFFIETVLAFYWNTGVIQNFNRYNNFPLMEAVNRRVNYWDEPNFEPDATETLKKLFAGTALKATVKFQKEANVQKTPVIITANYKFTKEVWDD-------RIIKYYWYPCPK
>Danaus_plexippus_plexippus_densovirus_KF963252
IVEELLDFQEDAKRKFITDLYEILEKKHQKTNTFQIVSPPSAGKNFFIETVLAFYWNTGVIQNFNRYNNFPLMEAVNRRVNYWDEPNFEPDATETLKKLFAGTALKATVKFQKEANVQKTPVIITANYKFTKEVWDD-------RIIKYYWYPCPK
>Dendrolimus_punctatus_densovirus_AY665654
IIERLLNYQNDAKKVFINDLWDILEKKRPKQNTFQIIGEPSAGKNYFIDAILSFYLNTGIILNFNRFSQFPLMEAVNRRVNVWNEPNFEPAALDTIKMLLAGDPLKVNVKYQKEQYLQKTPIIVMTNKVFPNDLAFHD------RVCTYFWKRAPF
>Helicoverpa_armigera_densovirus_HQ613271
AIEELLDFQTDKKREFVTDLWDVLEKKVPKKNTFQIISPPSAGKNFLLDAIFAFYWNVGMIRNFNKYESFPLMEAVDKRVNCWNEPNFEPSAEDTIKMILGGDPIKAAYKYESERNIARTPVIVMSNKNVFKINDAFED-----RIITYYWDRAEF
>Blattella_germanica_densovirus_AY189948
VVTELLTFQGENLVQFCRNLVDTLECNIPKRNCFVVCSPPSAGKNFFFDGVKDYYLNSGQMNNPNKYNQFAYQDCHNRRIIIWNEPNYEPREMENLKMLFAGDNLSANVKCKPQANVKRTPVIVLTNS-LPNFCQQTAFND---RVITYHWTQATF
>Blattella_germanica_densovirus-like_virus__JQ320376
IINKLLDYQGLAKKQFVTDLINVTDMNIPKCNCFLIVSPPSAGKNFLCDAIKDYYINVGQMQNPNKYNTFAYQDCHNRRLLIWNEPNYEPRETENLKMLFGGDNLSANVKCKPQANVKRTPVICMSNV---------VPRFANHRVITYYWNAAPF
>Cherax_quadricarinatus_densovirus_KP410261
IMDELVNYQEEAIIDFVTTLYNVLERKVPKLNCIVIHSPPSAGKNFFFDAVKDYYLNCGHLCNANKYNNFPFQDAEGRRIVLWNEPNYAPEFLEQIKEILGGDSTSVNVKYQSDTPVYRTPVIVLTNNKVSFMNHSAFID----RIRVFNWMAAPF
>Sea_star_densovirus_KM052275
VMDKLVQYQEDATLDFVNTLYNVLERKIPKLNCICIYSPPSAGKNFFFDAIKDYYINCGHLSNANKYNAFPFQDAEGRRIVLWNEPNYSPEFLEPIKELLGGDSTSVNVKYMHDTPVYRTPMIVLTNNVVSFMTHPAFKD----RVRVFNWMSAPF
>Dysaphis_plantaginea_densovirus_FJ040397
IAEELLCYQGDLISKFLYDVYAIVDKLVPKRNSMCVVSPPSAGKNFFFDAVASYFLNYGMFGTANKTNNFSWADGAGKRLVLWNEPNYETFHVEKIKELLGGDTTRVHVKYKGDQPLQGPPIFLLTNNTLSICNDPAFAD----RLVTYEWKSAPF
>Myzus_persicae_densovirus_AY148187
IAKELLNYQPEVVIEFLTTLYNVIDKRVPKLNSICIKSPPSAGKNFFFDAVASYLLSYGMFGTANKNNNFSWADGAGKRLVLWNEPNYEQYHIEKIKELLGGDTTRIHVKYANDVSVQRVPIIILTNNHLNIISHPAFND----RLRSYEWMSAGF
>Periplaneta_fuliginosa_densovirus_AF192260
ILNKLLAYQVVQIKSFLNTFYNVLERKLPKCNTICVWSPPSAGKNFFFDVYLHYLMNYGQLGIMNKTNNFSLQEATSKRVLLWNEPNYEDAYTDTLKMLTGGDALCVRVKQKKDCHVYKTPLIVLTNNMIGFMHELAFVD----RVKVYRWKQAPF
>Culex_pipiens_densovirus_FJ810126
ILEDFLKFQDEKITQFLVDLIDVLDKRVPKQNAFAVISPPSSGKNFFFDMIMAICLNYGQLGQANRHNLFAFQEAPNKRLLLWNEPNYESAMTDTIKMMMGGDPYTVRVKYQGDTHVTRTPVLILTNNYVSFLQESAFKD----RIKVYKWREAPF
>Helicoverpa_armigera_densovirus__JQ894784
IVTELLTFQESLIVEFLTNLVNVLDRKKPKLNTFVVYSPPTAGKNFFFDMIFGFCLSYGQLGQANKQNLFAFQEAPNKRVLLWNEPNYESSLTDTVKMMLGGDPYTVRVKNRGDQHVKRTPVIVLTNNIVPFMYEIAFNE----RIVQYKWNVAPF
>Galleria_mellonella_densovirus_L32896
IIDELLKYQEGLIVEFLTNLVNVLDRRIPKLNAFLVMSPPSAGKNFFFDMIFGLLLSYGQLGQANRHNLFAFQEAPNKRVLLWNEPNYESSLTDTIKMMFGGDPYTVRVKNRMDAHVKRTPVIILTNNTVPFMYETAFAD----RIIQYKWNAAPF
>Mythimna_loreyi_densovirus_AY461507
IIDELLKYQEGLIVEFLTNLVNVLDRRVPKLNAFLVMSPPSAGKNFFFDMIFGLLLSYGQLGQANRHNLFAFQEAPNKRVLLWNEPNYESSLTDTIKMMFGGDPYTVRVKNRMDAHVKRTPVIILTNNTVPFMYETAFAD----RIIQYKWNAAPF
>Diatraea_saccharalis_densovirus_AF036333
IVNELLKYQEDLIVEFLTNLVNVLDRRIPKLNAFLIISPPSGGKNFFFDMIFGLLLSYGQLGQANRHNLFAFQEAPNKRVLLWNEPNYESSLTDTIKMMFGGDPYTVRVKNRMDAHVKRTPVIILTNNTVPFMYELAFSD----RIIQYKWNAAPF
>Junonia_coenia_densovirus__KC883978
IIIELLKFQEDLIVEFLTNLVNVLDRRIPKLNAFLIISPPSAGKNFFFDMIFGLLLSYGQLGQANRHNLFAFQEAPNKRVLLWNEPNYESSLTDTIKMMFGGDPYTVRVKNRMDAHVKRTPVIILTNNTVPFMYETAFSD----RIIQYKWNAAPF
>Pseudoplusia_includens_densovirus_JX645046
IIVELLKYQEDLIVEFLTNLVNVLDRRIPKLNAFLILSPPSAGKNFFFDMIFGLLLSYGQLGQANRHNLFAFQEAPNKRVLLWNEPNYESSLTDTIKMMFGGDPYTVRVKNRMDAHVKRTPVIILTNNTVPFMYETAFSD----RIIQYKWDAAPF
>Echinococcus_granulosus_CDS25081
-------------QKFLDLAEEIMRRKVRSMNDM-----------------NKKFTYQETFRLMADYGQSY--NMIRKALETVRMMNVAHQRATDYADLSQKEEY--NVRNRSPSHLP-----LSKNQ--------ENHKWAIT------------
>Aleutian_mink_disease__virus_JN040434
LLATIKDM-GLNEQYLKKVLCTILTKQGGKRGCIWFYGPGGTGKTLLASLICKATVNYGMVTTS--NPNFPWTDCGNRNIIWAEECGNFGNWVEDFKAITGGGDVKVDTKNKQPQSI-KGCVIVTSNTNITKVTVGCVETNAHARMIKIRCMKT--
>Gray_fox_amdovirus_JN202450
-LINIINQMGLNEKVLKNIIATVLTKQSGKRGCIWFYGPGGTGKTLLANLICTAVKNFGMVTTS--NQNFPWTDCGNRNMIWLEECGNLGNFIEDFKAITGGGDIKVDTKNKQPQAI-KGVTVITSNKDITKVTIGAVETNVHSRIVKIRCVKT--
>Bufavirus_1_JX027296
-AWKLIKNNNMNPQKVYHAIMCCLNKQMGKRNTILLCGPASTGKSLLAQKIAKLVGNTGCYNPS--NANFPFNDCVNKNLIWIEEAGNLGTQVNSFKAIMSGQAIRLDQKGKGSKSIEPTPVVMTTNEDITRVIIGTELKVEHRRCLRFELKNKLS
>Porcine_parvovirus_PT4_PPU44978
---KIFSMHNWNYIKVCHAITCVLNRQGGKRNTILFHGPASTGKSIIAQHIANLVGNVGCYNAA--NVNFPFNDCTNKNLIWIEEAGNFSNQVNQFKAICSGQTIRIDQKGKGSKQIEPTPVIMTTNEDITKVRIGCEERPEHTRMLNINLTRKLP
>Canine_parvovirus_M19296
---QIFRMHGWNWIKVCHAIACVLNRQGGKRNTVLFHGPASTGKSIIAQAIAQAVGNVGCYNAA--NVNFPFNDCTNKNLIWIEEAGNFGQQVNQFKAICSGQTIRIDQKGKGSKQIEPTPVIMTTNENITIVRIGCEERPEHTRMLNIKLVCKLP
>Mouse_parvovirus_1_MPU12469
---KIFAFHGWNYIKVCHAICCVLNRQGGKRNTVLFHGPASTGKSIIAQAIAQAVGNVGCYNAA--NVNFPFNDCTNKNLIWVEEAGNFGQQVNQFKAICSGQTIRIDQKGKGSKQIEPTPVIMTTNENITVVKIGCEERPEHTRMLNIHLTHTLP
>Mouse_parvovirus_3_NC_008185
---KIFAFHGWNYIKVCHAICCVLNRQGGKRNTVLFHGPASTGKSIIAQAIAQAVGNVGCYNAA--NVNFPFNDCTNKNLIWVEEAGNFGQQVNQFKAICSGQTIRIDQKGKGSKQIEPTPVIMTTNENITVVKIGCEERPEHTRMLNIHLTHTLP
>Rat_parvovirus_NTU1_AF036710
---RIFAGHGWNYIKVCHAIACVLNRQGGKRNTVLFHGPASTGKSIIAQAIAQAVGNVGCYNAA--NVNFPFNDCTNKNLIWVEEAGNFGQQVNQFKAICSGQTIRIDQKGKGSKQIEPTPVIMTTNENITVVRIGCEERPEHTRMLNIHLTNTLP
>Bovine_parvovirus_2_AF406966
-VFDLLQFQGYDPVVAGYIIYAWSIRATGRRGALWFYGPGQTGKSIMARAMATCSVRYGCVNWT--NSNFPFQDLANCQIGWWEEGVITEDIVESAKALLSGGKIRVDRKCRDSVEITPPPFVITSNNDMTLVQGGNQVSFVHKRMIKFNFNKRLP
>Porcine_parvovirus_4_GQ387499
-MHYIFAINNYDPKIASVIMYFWSMKQTGKRNCVWFYGPATTGKTNMAQAICHSSANYGNVNWN--NANFPFQDIAGAQVGWWEEGKMTGDMVEAAKALLGGTALRIDRKCMQSIEVNSPPFLITSNVDMTIVQEGSFVSFEHQRMIKFSFNMTLP
>Eidolon_helvum_parvovirus_1_JQ037753
---DIFRLNGYEPSLVARYMACWAVGHWPKRRALWLWGPASTGKTVIAAAIAAQAPSYGCVNWT--NANFPFNDCHCQPLVWWEEGRMTENIVEVAKAILGGSPVRLDVKNKGSEDFLPTAVIITSNGDLTVTVDGPVISDAHQRMCMIRLERVLP
>Human_parvovirus_4_AY622943
-VAQLFSLNGYNPVDAAWYFAAWARGVWPKRRAIWLWGPASTGKTLLAAAIANLSPSYGCVNWT--NQNFPFNDCHCQSLVWWEEGRMTENIVEVAKAVLGGAPVRLDVKNKGSEDYIPTCVIITSNGDLTVTVDGPVVSTQHQRITMFQFQRMVP
>Bovine_hokovirus_EU200669
-VIELFKLNAYDPEDAAWYFAAWAQGIWPKRRALWLWGPASTGKTLLAAAIANISPSYGCVNWT--NQNFPFNDCHCQSLVWWEEGRMTENIVEVAKAILGGAPVRLDVKNKGSEDFIPTCVIITSNGDLTVTVDGPVVSTVHQRITMFQFNRLVP
>Porcine_hokovirus_EU200677
-VTELFRINGYDPEDAAWYFAAWAAGAWAKRRAMWLWGPASTGKTLLAAAIAAVAPSYGCVNWT--NQNFPFNDCHCQSLVWWEEGRMTENIVEVAKAILGGAPVRLDVKNKGSEDFIPTCVIITSNGDLTVTVDGPVVSTAHQRITMFQFQRLVP
>Human_parvovirus_B19
```

-IVKLLLCQNYDPLLVGQHVLKWIDKKCGKKNTLWFYGPPSTGKTNLAMAIAKSVPVYGMVNWN--NENFPFNDVAGKSLVVWDEGIIKSTIVEAAKAILGGQPTRVDQKMRGSVAVPGVPVVITSNGDITFVVSGNTTTTVHARMVKLNFTVRCS
>Goose_parvovirus__GPU25749
-VYQILKMNNYNPQYIGSILCGWVKREFNKRNAIWLYGPATTGKTNIAEAIAHAVPFYGCVNWT--NENFPFNDCVDKMLIWWEEGKMTNKVVESAKAILGGSAVRVDQKCKGSVCIEPTPVIITSNTDMCMIVDGNSTTMEHRRMFQIVLSHKLE
>Adeno-associated_virus_1_AF063497
-IYRILELNGYEPAYAGSVFLGWAQKRFGKRNTIWLFGPATTGKTNIAEAIAHAVPFYGCVNWT--NENFPFNDCVDKMVIWWEEGKMTAKVVESAKAILGGSKVRVDQKCKSSAQIDPTPVIVTSNTNMCAVIDGNSTTFEHQRMFKFELTRRLE
>Bat_adeno-associated_virus_GU226971
-IYRLFRMNGYDPAYAGSVLLGWCRTGFGKRNTVWLFGPATTGKTNLAEAISHSVPFYGCVNWT--NENFPFNDCVDKMIIWWEEGKMTSKVVESAKAILGGSKVRVDQKCKNSQQIEPTPVIITSNTNMCEVVDGNSTTFEHRRMFKFELTVRLQ
>California_sea_lion_adeno-associated_virus_1_JN420372
-IYQLFKMNGYDPAYLGSILLGWCQGRFGKRNTVWLYGPATTGKTNLAEAIAHSVPFYGCVNWT--NENFPFNDCVDKMLIWWEEGKMTSKVVESAKAILGGSKVRVDQKCKSSVQIDSTPVIITSNTDMCCVIDGNSTTFEHRRMFRINLEQRLS
>Adeno-associated_virus_5_AF085716
-IWQIFEMNGYDPAYAGSILYGWCQRSFNKRNTVWLYGPATTGKTNIAEAIAHTVPFYGCVNWT--NENFPFNDCVDKMLIWWEEGKMTNKVVESAKAILGGSKVRVDQKCKSSVQIDSTPVIVTSNTNMCVVVDGNSTTFEHQRMFKFELTKRLP
>Turkey_parvovirus__GU214706
-AIRLCSYQGYSPKYVARLILCWLSGQAGKKNALYFHGPANTGKTMMAESICKMVQIYGNVNHN--NKNFPFNDCHNKAVLWWEECSMTEEHVESAKCIMGGSSVRIDKKNQDSVLLCKTPIVITSNNDITQVSSRNAISTVHARCLKFTFNNWLT
>Porcine_bocavirus_3_JF429834
KVVRLLNIQGYNPIQVGHWVATVLSKKAGKQNTICFFGPASTGKTNLAKAIANAVKVYGCVNHL--NKSFVFNDCQNKLICWWEEAVMHNDWVEPAKCLMGGTSFRVDRKHKDSAEQPHTPLILSTNHDIYTVVGGNTVTTVHERVVQFNFMKTLP
>Porcine_bocavirus_5_HQ223038
-VIRLLNFQGYNYWQVGHWLCCVLDKKSGKQNTVSFYGPASTGKTNLAKAIVNAVNLFGNVNHL--NKNFVSNDCSNKLIVWWEEALMHTDWVEPAKCVLGGTTVRVDRKHKDSQLLPQTPCIISTNNNIYECVGGNHVSHVHCRVVQLNFMKTLP
>Human_bocavirus_1_JQ923422
-ALQLLLIQGYNPLAVGHALCCVLNKQFGKQNTVCFYGPASTGKTNMAKAIVQGIRLYGCVNHL--NKGFVFNDCRQRLVVWWEECLMHQDWVEPAKCILGGTECRIDVKHRDSVLLTQTPVIISTNHDIYAVVGGNSVSHVHARVIQLNFMKQLP
>Human_bocavirus_4_FJ973561
-ALKLLLIQGYNPLQVGHAICCVLNKQMGKQNTICFYGPASTGKTNFAKAIVQGVRLYGCVNHL--NKGFVFNDCRQRLIIWWEECLMHQDWVEPAKCILGGTECRIDVKHKDSVLLQQTPVIISTNHDIYSVVGGNTVSHVHARVLQLNFMKQLP
>Feline_bocavirus_JQ692585
-AWRLLLKQGYNPLQVGHWICCVLHKKAGKQNTLNFFGPASTGKTNLAKAIVNAIKLYGCVNHQ--NKNFIFNDCAAKLVVWWEECLMHSDWVEQAKCILGGTEFRIDRKHRESHLLPQTPVIISTNNNIYQTLGGNSVSHVHERVVQFNFMTRLE
>Canine_minute_virus_FJ214110
-VWKLLTLQGYNPWQFGHWLLCVLDKKAGKQNTINFYGPASTGKTNLAKAIVNAVQLYGCVNHQ--NKNFVFNDCTAKLICWWEECIMTTDWVEQAKCIMGGTQFRIDRKHKDSHLLPQTPLIISTNHNIYEVTGGNTTTHVHSRVVQFNFMKQLE
>Porcine_bocavirus_1_HM053693
RVFRLLNFQGYNPWQAGHWVCCVLDKKSGKQNTLCFYGPASTGKTNLAKSIVQACKLYGCVNHQ--NKNFVFNDCAAKLIVWWEEALMHNDWVEQAKCILGGTEFRIDRKNRDSMLLPKTPVVISSNNDLYTVVGGNTVSQVHSRIVQWNFMKMLE
>Canine_bocavirus_1_JN648103
-AIQLLIFQGYNPWQVGHWLCCVLHKTAGKQNTVCFFGPASTGKTNFAKAIVNAVKLYGCVNHQ--NKNFVFNDCASKLVNWWEECLMHNDWVEQAKCLLGGTEFRIDRKHKDSQLLPQTPVVISTNHDVYTVVGGNTTTMVHARIVQFNFMKQLS
>California_sea_lion_bocavirus_1_JN420361
-AYQLFAIQGYNAWQAGHWLCCVLNKTAGKQNTVNFYGPASTGKTNMAKAIVQAVKLYGCVNHQ--NKNFLFNDCASKLVCWWEECVMNSEWVEQSKCILGGTEFRIDRKHKDSMLLPQTPVMISTNNDIYTVVGGNTVTGVHSRVVQFNFMKQLP
>Schmidtea_mediterranea_GAKN01010353
---------------------IYLQGNILYDNLFYSIGPSNTRKTFFVQLLTVDMKV-GCIHNMNNKNTFWTQDVDGKDVVVAEELMLNMENVDYFKLMLEGSNMRIEIKNKPAVNWKRIPVIVTSNQ-WIWQFVSNHQEALQNRMLVIKFNQPID
>Neobenedenia_melleni_GW918566
ILNEIFIKNKINKNFFISSLKNIIFKKYFKINTLLFYGNPNSGKSLIANLISQVYKS-KIVSNVASSSEFYLSNFDDANIIICEELFITPANVDDFKNILGGTSIVVNKKYERRGRINRIPILITSNYDLTHGFCSQLDTSALLRMIVFHFNYDIT
>Aedes_albopictus_densovirus_1_AY095351
WIEYLFKENNIDIIHFLAWNEIIKTKRYKKINGMVLEGITNAGKSLILDNLLAMVKP-EEIPRERDNSGFHLDQVPGAGSILFEEPMITPVNVGTWKLLLEGKTIKTDVKNKDKEPIERTPTWITTATPITNNIDMNETSQILQRIKLYIFKKSIQ
>Aedes_aegypti_densovirus_1_M37899
WIEYLFKENNINIIHFLAWNEIIKTKRYKKINGMVLEGITNAGKSLILDNLLAMVKP-EEIPRERDNSGFHLDQVPGAGSILFEEPMITPVNVGTWKLLLEGKTIKTDVKNKDKEPIERTPTWITTATPITNNIDMNETSQILQRIKLYILKKSIQ
>Anopheles_gambiae__densovirus_EU233812
WIEYLFKENNINIIHFLAWNEIIKTKRYKKINGMVLEGITNAGKSLILDNLLAMVKP-EEIPRERDNSGFHLDQVPGAGSILFEEPMITPVNVGTWKLLLEGKTIKTDVKNKDKEPIERTPTWITTATPITNNIDMNETSQILQRIKLYIFKKSIQ
>Aedes_aegypti_densovirus_2_FJ360744
WIEYLFKENNINIIHFLAWNEIIKTKRYKKINGMVLEGITNAGKSLILDNLLAMVKP-EEIPRERDNSGFHLDQVPGAGSILFEEPMITPVNVGTWKLLLEGKTIKTDVKNKDKEPIERTPTWITTATPITNNIDMNETSQILQRIKLYIFKKSIQ
>Mosquito_densovirus_BR/07_GU452720
WIEYLFKENNINIIHFLAWNEIIKTKRYKKINGMVLEGITNAGKSLILDNLLAMVKP-EEIPRERDNSGFHLDQVPGAGSILFEEPMITPVNVGTWKLLLEGKTIKTDVKNKDKEPIERTPTWITTATPITNNIDMNETSQILQRIKLYIFKKSIQ
>Culex_pipiens_pallens_densovirus_EF579771
WIEYLFKENNINIIHFLAWNEIIKTKRYKKINGMVLEGITNAGKSLILDNLLAMVKP-EEIPRERDNSGFHLDQVPGAGSILFEEPMITPVNVGTWKLLLEGKTIKTDVKNKDKEPIERTPTWITTATPITNNIDMNETSQILQRIKLYIFKKSIQ
>Aedes_albopictus_densovirus_2_X74945
WIEYMFKENNINIIHFLAWNEIIKPKRYKKINGMVLEGITNAGKSLILDNLLAMVKP-EEIPRERDNSGFHLDQLPGAGSVLFEEPMITPVNVGTWKLLLEGKTIKTDVKNKDKEPIERTPTWITTATPITNNVHMNETSQILQRIKLYIFKKSIQ
>Haemagogus_equinus_densovirus_AY605055
WIEYMFKENNINMIHFLAWNEIIKTKRYKKINGMVLEGITNAGKSLILDNLLAMVKP-EEIPRERDNSGFHLDQLPGAGSVLFEEPMITPVNVGTWKLLLEGKTIKTDVKNKDKEPIERTPTWITTATPITNNVDMNETSQILQRIKLYIFKKSIQ
>Aedes_albopictus_densovirus_3_AY310877
WIEYMFKENNINIIHFLAWNEIIKTKRYKKINGMVLEGITNAGKSLILDNLLAMVKP-EEIPRERDNSGFHLDQLPGAGSVLFEEPMITPVNVGTWKLLLEGKTIKTDVKNKDKEPIERTPTWITTATPITNNVDMNETSQILQRIKLYIFKKSIQ
>Taenia_multiceps_JR938527
---MTPFINGFSVLVMPYRHISLS-LIQHFQFDNLLNDAGALMEEPRITMTTKDDFKLLLGGIGLEVDVKYSGHKWLERVLVIQATNEDIIFRLSGQNRKCFTN------------
>Taenia_multiceps_JR929085
---------ITTFLLLYTKVVDVVDEKMDKVNMLCFYRTTNAGKSLLAGLITAPLVV-AAITRCGYQTAFQFDNLLHKTGALME----------------S---
>Penaeus_monodon_penstyldensovirus_2_AY124937
WIKYMLANNDIRVPEILAWILIVADKKLDKINTLVLQGPTGTGKSLTIGALLGKLNT-GLVTRTGDSNTFHLQNLIGKSYALFEEPRISQITVDDFKLLFEGSDLEVNIKHQESEIMGRIPIFISTNKDIDYWVPPADGKALQTRTKTFHLTSQIK
>Penaeus_monodon_penstyldensovirus_1_GQ411199
WIKYMLANNDIRVPEILAWIIIVADKKLDKINTLVLQGPTGTGKSLTIGALLGKLNT-GLVTRTGDSNTFHLQNLIGKSYALFEEPRISQITVDDFKLLFEGSDLEVNIKHQESEIMGRIPIFISTNKDIDYWVPPADGKALQTRTKTFHLTRQIK
>Penaeus_stylirostris_penstyldensovirus_2_GQ475529
WIKYMLANNDIRVPEILAWIIIVADKKLDKINTLVLQGPTGTGKSLTIGALLGKLNT-GLVTRTGDSNTFHLQNLIGKSYALFEEPRISQITVDDFKLLFEGSDLEVNIKHQESEIMGRIPIFISTNKDIDYWVPPADGKALQTRTKTFHLTRQIK
>Hymenolepis_diminuta_LM393614
-LYSIFTNNDIDFGLFLAEVDKIIRMTYPRINALVLRGPTSTGKTLIAKNIVKPYNY-ETVSRDGDATAFYLQNLLDHDVALMEEPHISMTTVQNFKELFAGSPLIVQVKNHAPRELKRIPCIVTTNQSLTDSLIDAESEPIRRRIIEYLLYRPIS
>Hymenolepis_diminuta_LM395168
-LYSNFTNNGIDFGLFIAEVDKIIRMSYPRINALVLRGPTSTGKTLIAKNIVKPYNY-GTVSRDGDATAFYLQNLLDHDVALMEEPHISMTTVQNFKELFAGSPLIVQVKNHAPRELKRIPCIITTNQSLTDSLIDAESEPIKKRIIEYLLYRPIS
>Schistosoma_mansoni_XP_002571349
WFDMLMEKNDIDKKVFCASVSTIMNKKVKRVNTLCLEGPTTTGKSLLLKLICGEYNY-GTVQRSGDHSQFFLQNLLKKTVALMEEPRITPITVQDFKLLLGGEPLDIHVKHQDDCRLPRIPVLISTNHELGFYTSSLDKDALYNRTHYYKFKCQLG
>Opisthorchis_viverrini_XP_009173661
WFDMLMEKNDIDKKVFCASVSTIMNKKVKRVNTLCLEGPTTTGKSLLLKLICGEYNY-GTVQRSGDHSQFFLQNLLKKTVALMEEPRITPITVQDFKLLLGGEPLDIHVKHQDDCRLPRIPVLISTNHELGFYTSSLDKDALYNRTHYYKFKCQLG
>Schistosoma_mansoni_CCD58408
WFDMLMEKNDIDKKVFCASVSTIMNKKVKRVNTLCLEGPTTTGKSLLLKLICGEYNY-GTVQRSGDHSQFFLQNLLKKLLPSWK---------------------NQE--
>Dicrocoelium_dendriticum_LK428994
FLDNLLAANKIDKDKFCKAIHEIMNKKIDRLNCLCIEGPTTTGKSLVLKMICQNYHC-GTVQRSGDHSQFFLQNLVDKSIALMEEPRMTIATVNDFKELLGGSPFDIHVKHSPDVTLNRLPVLISTNHSLGTYITSIDAAAIYERCHTFHFRVRVG
>Dicrocoelium_dendriticum_LK459200
FLDNLLAANKIDKDKFCKAIHEVMNKKIDRLNTLCIEGPTTTGKSLVLKMICQNYHC-GTVQRSGDHSQFFLQNLVDKSVALMEEPRITLATVNDFKELLGGSPFDIHVKHSPDVTLSRLPVLISTNHSLGAYITSIDAAAIY-------------
>Schistosoma_mattheei_LM181082
-------MN----------------RQKFGINTLCIEGPTTTGKTLILKLITQNYTF-GTVRMSGNHSKFFLQNLNNKSVALMEQPRITPVNVNDFKQLLGGSSLDIHIKHQSDVTLQRIPVLISTNSNLGLYINSEDKKAIYKRCITYHFTQSIG
>Schistosoma_margrebowiei_LL888002
WFETFFAANEINPKQFLNDIQTIIDKTKLRVNTFCLEGPTTTGKTLILKLIVQNYTY-GTVQRSGDHSQFFLQNLNNKSVALMEEPRITPITVNDFKELLGGSPFDIHITHQPDVTLPRIPVLISTNSNLGTYINSIDKDAIYKRCITYHLTKQIG
>Clonorchis_sinensis_GAA57952
WLDLLLHVNRINKQNFLTDLTRVMNKQIDRKNAFVLEGPTTTGENIIRKTHC------GQLHIW------------DRTEIWRSQSVLPNEPTE--
>Opisthorchis_viverrini_XP_009170655
WLDQLMSANKINKQQFLTDLTLVMNKVCTRKNASVIEGPTTTGKTLFVKLIAENYIY-GTVQRPEEQETKPATTTATTTVAPKKEEPETVSIMETKRQRLDGPNLAVAKHAQSLFGIHYKTDVTVTRKHLTLAQYKPADTGITQTCIPYQIFEFWT
>Opisthorchis_felineus_GBJA01006694
WLDLLFHANKINKRDFLVNLTQIMNKDIDGRNAFVIEGPTTTGKTLLVKLIAENYIY-GTVKRSGDRSQCFLMKLLDKALALVEEPRITQLTVNHFKALLGGNAFDIRVKHQKDERLTRLPVLITTNTDLTYQCLGEDAKAIKERCFIYRFFVHVG
>Opisthorchis_felineus_GBJA01010281
WLDQLMQVNNIDKKDILHSLTLIMNKSLKRKNAFVIEGPTTTGKTLFVKLIAENYVY-GTVQRSGDHSQFFLMNLLNKTLALMEEPRITQLSVNDFKELLGGNPFDIHVKHQKDELHY-------GTH----------------RRSFWCW-----
>Clonorchis_sinensis_GAA40146
WLDLLLHVNRINKQEFLTNITKIMNREIDRKNAFVIEGPTTTGKTLFVKLIAESYIY-GTVQRSGDHSQFFLMNLLNKALALMEEPRITQLTVNDFKELLGGNAFDIHVKHQKDERLTRFPVLITTNNDLTYYVLGEDDKAIKERCFYYKFFVSMR
>Opisthorchis_felineus_GBJA01005901
WLDLLFYVNKINKQEFLIDITRIMNKEVDRTNAFVIEGPTTTGKTLFVKLIAENYIY-GTVQKSGDHSQFFLMNLLNKALALMEKPRITQLTVNYFKELLGGNPFDIHVKHQKDERLNRLPVLITTNNDLTYYVLGEDGKAIKERCFTYKFFVKVG
>Opisthorchis_felineus_GBJA01002122
WLDLLFQVNKINKQTFLCNITRIMNKEIDRKNAFVIEGPTTTGKTLFVKLIAENYIY-GTVQRSGDHSQFFLMNLLNKALALMEEPRITQLTVNDFKELLGGNPFDIHVKHQKDERLNRLPVLITTNNDLTYYVLGEDGKAIKERCFAYKFYVKVG
>Clonorchis_sinensis_GAA32750
WLDLLLHVNRINKQDFLCNLTKIMNKQIDRKNAFVIEGPTTTGKTLFVKLIAESYIY-GTVQRSGDHSQFFLMNLLNKALALMEEPRITQLTVNDFKELLGGNAFDIHVKHQKDERLTRLPVLITTNNDLTYYVLGEYDIYMTWRLRQLLIRNATK
>Clonorchis_sinensis_GAA56553
WLDLLLFVNRINKQQFLADLTRVMNKEVDRKNAFVLEGPTTTGKTLFVKLIADSYIY-GTVQRSGDHSQFFLMNLLNKALALMEEPRITQLTVNDFK---
>Clonorchis_sinensis_GAA57641
WLDLLLFVNRINKQQFLTDLTRVMNKQIDRKNAFVLEGPTTTGKTLFVKLIADNYIY-GTVQRSGDHSQFFLMNLLNKALALMEEPRITQLTVNDFKELLGGNAFDIHVKHQKDERLTRLPVLITTNNDLTYYVLGEDGKAIKERCFYYKFFVKVG
>Clonorchis_sinensis_GAA57639
WLDRLLFVNRINKQQLLADLTRVMNKQVDRKNAFVLEGPTTTGKTLFVKLIADNYIY-GTVQRSGDHSQFFLMNLLNKALALMEEPRITQLTVNDFKELLGGNAFDIHVKHQKDERLTRLPVLITTNNDLTYYVLGEDGKAIRERCFYYKFFVKVG
>Clonorchis_sinensis_GAA57638
WLDRLLFVNRINKQEFLADLTRVMNKQVDRKNAFVLGGPTTTGKTLFVKLIADNYIY-GTVQRSGDHSQFFLMNLLNKALALMEEPRITQLTVNDFKELLGGNAFDIHVKHQKDERLTRLPVLITTNNDLTYYVLGEDGKAIKERCFYYKFFVKVG

>Clonorchis_sinensis_GAA57954
WLDRLLFVNRINKQKFLADLTRVMNKQVDRKNAFVLEGPTTTGKTLFVKLIADNYIY-GTVQRSGDHSQFFLMNLLNKALALMEEPRITQLTVNDLKELLGGNAFDIHVKHQKDERLTRLPVRITTNNDLTYYVLGEDGKVIKERCFYYKFFVKVG
>Clonorchis_sinensis_GAA57640
WLDRLLFVNRIDKQKFLCDLTRVMNKQVDRKNAFVLEGPTTTGKTLFVKLIADNYIY-GTVQRSGDHSQFFLMNLLNKALALMEEPRITQLTVNDFKELLGGNAFDIHVKHQKDERLTRLPVLITTNNDLTY------------------------
>Opisthorchis_viverrini_XP_009177542
WLDQLMWVNRIDKKQFLIDLTQIMNKAYKRKNAFVIEGPTTTGKTLFVKLIAENYIY-GTVQRSGDHSQFFLMNLLNKSLGLMEEPRITQLTVNDFKELLGGNPFDIHVKHQKDERLQRLPVLITTNNNLVYYVLDPDGKAILERCFYYKFVVKVG
>Opisthorchis_viverrini_XP_009170634
WLDQLMSANKINKQQFLTDLTLVMNKVCTRKNAFVIEGPTTTGKTLFVKLIAENYIY-GTVQRSGDHSQFFLMNLLNKALALMEEPRITQLTVNDFKELLGGNPFDIHVKHQKDERLQRLPVLITTNNNLVYYVLDPDGKAILERCFYYKFLVKVG
>Opisthorchis_viverrini_XP_009176824
WLDQLMAANKINKQQFLTDLTLVMNKVCTRKNAFVIEGPTTTGKTLFVKLIAENYIY-GTVQRSGDHSQFFLMNLLNKALALMEEPRITQLTVNDFKELLGGNPFDIHVKHQKDERLQRLPVLITTNNNLVYYVLDPDGKAILERCFYYKFLIKVG
>Opisthorchis_felineus_GBJA01008850
WLDRLMLVNNINKRELLTSLTLVMNKSMNRKNAFVIEGPTTTGKTLFVKLVAENYVY-GTVQRSGDHSQFFLMNLLNKTLALMEEPRITQLTVNDFKELLGGNPFDIHVKHQKDERLERLPVLITTNNRLTYYVLDSDAKAILERCFYYTFTVKVG
>Opisthorchis_viverrini_XP_009177530
WLDLLINVNHINKRELLVSLTAVMNKLCTRKNAFVIEGPTTTGKTLFVKLVAENYVY-GTVQRSGDHSQFFLMNLLNKTLALMEEPRITQLTVNNFKELLGGNPFDIHVKHQKDERLERLPVLITTNNPLTYYVLDADGKAILERCFYFKFHVKVG
>Opisthorchis_viverrini_XP_009178033
WLDELIRVNNINKRELLVSLTAIMNKLCTRKNAFVIEGPTTTGKTLFVKLIAENYVY-GTVQRS------------------------------------GDH---
>Opisthorchis_viverrini_XP_009177702
WLDQLIQVNNINKRELLVCLTSVMNKLCTRKNAFVIEGPTTTGKTLFVKLVAENYVY-GTVQRSGDHSQFFLMNLLNKTLALMEEPRITQLTVNDFKELLGGNPFDIHVKHQKDERLERLPVLITTNNPLTYYVMDADGKAIL-------------
>Opisthorchis_viverrini_XP_009177971
--MKLLNKTLALMEEPRITQLTVNDFKELLGGNPFDIHVKHQKDERLERLPVLITTNNPLTYYVMDADGKAILE------------
>Opisthorchis_viverrini_XP_009177942
WLDELIRVNNINKRDLLVALTAVMNKLCTRKNAFVIEGPTTTGKTLFVKLVAENYVY-GTVQRSGDHSQFFLMNLLNKTLALMEEPRITQLTVNDFKELLGGNPFDIHVKHQKDE---
>Opisthorchis_viverrini_XP_009177808
WLDQLIRVNNINKRELLACLTSVMNKLCTRKNAFVIEGPTTTGKTLFVKLVAENYE----------------------------EPRITQLTVNDFKELLGGNPFDIHVKHQKDERLDRLPVLITTNNPLTYYVMDADGKAILER-----------
>Opisthorchis_viverrini_XP_009177798
-----------------------------RKNAFVIEGPTTTGKTLFVKLVAENYVY-GTVQRSGDHSQFFLMNLLNKTLALMEEPRITQLTVNDFKELLGGNAFDIHVKHQKDERLDRLPVLITTNNPLTYYVMDADGKAILERCFYFKFHVKVG
>Opisthorchis_felineus_GBJA01002124
WLDQLMAVNNINKKEMLHSLTLVMNKTMKRKNAFVIEVPTTTGKTLFVKLIAENYVY-GTVQRSADHSPFFLMNLLNKTLALMEEPRITQLTVNDFKELLGGNPFDIHVKHQKDERLDRLPVLITTNNRLTYYVLDSDAKAILERCFYYHFNVKVG
>Opisthorchis_felineus_GBJA01002120
WLDQLMLVNNINKKDMLHALTLVMNKTMKRKNAFVIEGPTTTGKTLFVKLIAENYVY-GTVQRSGDHSQFYLMNLLNKTLALMEEPRITQLTVNDFKELLGGNPFDIHVKHQKDERLERLPVLITTNNRLTYYVLDSDAKAILERCFYYHFTAQLR
>Opisthorchis_felineus_GBJA01002117
WLDQLMLVNNINKKEMLHSLTLVMNKTMKRKNAFVIEGPTTTGKTLFVKLIAENYVY-GTVQRSGDHSQFFLMNLLNKTLGFS---
>Moniezia_expansa_JL291017
----------------------------------VLQGPSNTGKSLLAKLIVSGYNY-ATVARSTESNNFIFQNLLGKTAALMEEPFITKATVNDFKQLLGGERMEIGIKHRDREWLERVPIICTTNDQLADRCNSVDCQAIQNRCVYYRLFKTIK
>Echinostoma_caproni_LL285499
WLDRLLAVNNIDKNKFLTQLTDIMNKKENRVNAFVIQGPTTTGKFQYSSHLSSHRQV-LDVKSP--HGELH----LNSPEEWRPLPVLPHELTKQGSCAYG----RTQDYAINGQRLQRTPGRKTIRYPCETPTRCPAQTPTRPHFYQYRHMSVFT
>Echinostoma_caproni_LL284665
WLDRLLAVNNIDKTRFLTQISNIMNKREQRINALVIQGPTTTGNL---
>Echinostoma_caproni_LL286487
WLDRLLAVNNIDKNRFLTQLTNIMNKKEQRINAFVIQGPTTTGK-FLCSSHLSSHRQILDAKSPHGELHLW-----NSSTKWGPFAILSHEFAEQGC--------RPHGRASNHPAHG--------------------------------------
>Echinostoma_caproni_LL274983
WLDQLLAVNHIDKNRFLTQVTNIMNKKEQRINAFVIQGPTTTGKL---
>Echinococcus_granulosus_W6UKP4
-LEDMFLANEIALVDFAITLRIIMNCEDEKINTLVLYGPTNTGKSLICKLTTTFLEH-GSVMRRQGASAFAYENLLNRKVALMEEPGICAANQQDLKQILGGETFKGPKDMQTTQQAPQ-PVQSTAQHPPAPATPTPH------------------
>Echinococcus_multilocularis_U6HF69
WLEDRFSANEIAVVDFAITLRIIMNCEDEKINALVLYGPTSTGKSLICKLTTTFLEH-GSVMRRQEASAFAHENLLNRKVALMEEPKICAANQQDLKQILGGEPFEVHIKYQNPDLLERLPVIVTTNEPLGVRLSDVDAAATEGRCKIYTLDKQIC
>Echinococcus_granulosus_EUB54832
-LEDMFSANEIAVVDFAITLRIIMNCEDEKINTLVLYGPTNTGKSLICKLTTTFLEH-GCVTRRQEASAFAYENLLNREVALMEEPRICAANQQDLKQILGGEPFEVSVKYQNPDLLERLPVIVTTNEPLGVRLSDVDAAAIEGRCKIYTLDKQIC
>Echinococcus_multilocularis_CDI70496
WLEDMFSANEIAVVDFAITLRIIMNCEDEKINTLVLYGPTNTGKSLICRLTTTFLEH-GSVMRRQEASAFA----------------ICAANQQDLKQILGGEPFEVSVKYQNPDLLERLPVIVTTNEPLDVRLSDVDAAAIEGRCKIYTLDKQIC
>Echinococcus_multilocularis_CDS35594
WLEDMFSANEIAVVDFAITLRIIMNCEDEKINTLVLYGPTNTGKSLICRLTTTFLEH-GSVMRRQEASAFA----------------ICAANQQDLKQILGGEPFEVSVKYQNPDLLERLPVIVTTNEPLDVRLSDVDAAAIEGRCKIYTLDKQIC
>Echinococcus_multilocularis_U6HA22
WLEDMFSANEIAVVDFAITLRIIMNCEDEKINTLVLYGPTNTGKSLICRLTTTFLEH-GSVMRRQEASAFAYENLLNRKVALMEEPRICAANQQDLKQILGGEPFEVSVKYQNPDLLERLPVIVTTNEPLDVRLSDVDAAAIEGRCKIYTLDKQIC
>Echinococcus_multilocularis_A0A087VWP3
WLEDMFSANEIAVVDFAITLRIIMNCEDEKINTLVLYGPTNTGKSLICRLTTTFLEH-GSVMRRQEASAFAYENLLNRKVALMEEPRICAANQQDLKQILGGEPFEVSVKYQNPDLLERLPVIVTTNEPLDVRLSDVDAAAIEGRCKIYTLDKQIC
>Echinococcus_multilocularis_U6HLE6
WLEDMFSANEIAVVDFAITLRIIMNCEDEKINTLVLYGPTNTGKSLICRLTTTFLEH-GSVMRRQEASAFAYENLLNRKVALMEEPRICAANQQDLKQILGGEPFEVHIKYQNPDLLERLPVIVTTNEPLGVRLSDVDAAAIEGRCKIYTLDKQIC
>Echinococcus_multilocularis_A0A068XWU9
WLEDMFSANEIAVVDFAITLRIIMNCEDEKINTLVLYGPTNTGKSLICRLTTTFLEH-GSVMRRQEASAFAYENLLNRKVALMEEPRICAANQQDLKQILGGEPFEVHIKYQNPDLLERLPVIVTTNEPLGVRLSDVDAAAIEGRCKIYTLDKQIC
>Echinococcus_multilocularis_CDS36723
WLEDMFSANEIAVVDFAITLRIIMNCEDEKINTLVLYGPTNTGKSLICRLTTTFLEH-GSVMRRQEASAFAYENLLNRKVALMEEPRICAANQQDLKQILGGEPFEVHIKYQNPDLLERLPVIVTTNEPLGVRLSDVDAAAIEGRCKIYTLDKQIC
>Echinococcus_multilocularis_U6HHY9
WLEDMFSANEIAVVDFAITLRIIMNCEDEKINTLVLYGPTNTGKSLICRLTTSFLEH-GSVMRRQEASAFAYENLLNRKVALMEEPKICAANQQDLKQILGGEPFEVHIKYQNPDLLERLPVIVTTNEPLGVRLSDVDAAAIEGRCKIYTLDKQIC
>Echinococcus_multilocularis_CDS36816
WLEDMFSANEIAVVDFAITLRIIMNCEDEKINTLVLYGPTNTGKSLICRLTTSFLEH-GSVMRRQEASAFAYENLLNRKVALMEEPKICAANQQDLKQILGGEPFEVHIKYQNPDLLERLPVIVTTNEPLGVRLSDVDAAAIEGRCKIYTLDKQIC
>Echinococcus_multilocularis_U6HDP5
WLEDMFSANEIAVVDFAITLRIIMNCEDEKINTLVLYGPTNTGKSLICRLTTSFLEH-GSVMRRQEASAFAYENLLNRKVALMEEPKICAANQQDLKQILGGEPFEVHIKYQNPDLLERLPVIVTTNEPLGVRLSDVDAAAIEGRCKIYTLDKQIC
>Echinococcus_multilocularis_U6HC26
WLEDMFSANEIAVVDFAITLRIIMNCEDEKINTLVLYGPTNTGKSLICRLTTSFLEH-GSVMRRQEASAFAYENLLNRKVALMEEPKICAANQQDLKQILGGEPFEVHIKYQNPDLLERLPVIVTTNEPLGVRLSDVDAAAIEGRCKIYTLDKQIC
>Echinococcus_multilocularis_A0A077RCT5
WLEDMFSANEIAVVDFAITLRIIMNCEDEKINTLVLYGPTNTGKSLICRLTTSFLEH-GSVMRRQEASAFAYENLLNRKVALMEEPKICAANQQDLKQILGGEPFEVHIKYQNPDLLERLPVIVTTNEPLGVRLSDVDAAAIEGRCKIYTLDKQIC
>Echinococcus_multilocularis_U6H9L2
WLEDMFSANAIAVVDFAITLRIIMNCEDEKINTLVLYGPTNTGKSLICKLMTSFLEP-GSVMRR--------------------PGR--------------RQPS---
>Echinococcus_multilocularis_U6HNK1
WLEDMFSANAIAVVDFAITLRIIMNCEDEKINTLVLYGPTNTGKSLICKLMTSFLEH-GSVMRRQEASAFAYEDLLNRKVALMEEPKICAANQQDLKQILGGEPFEVHIKYQNPDLLERLPVVVTTNEPLGVRLSDVDAAATEGRCKSYTLDKQIC
>Echinococcus_multilocularis_CDS36692
WLEDMFSANAIAVVDFAITLRIIMNCEDEKINTLVLYGPTNTGKSLICKLMTSFLEH-GSVMRRQEASAFAYEDLLNRKVALMEEPKICAANQQDLKQILGGEPFEVHIKYQNPDLLERLPVVVTTNEPLGVRLSDVDAAATEGRCKSYTLDKQIC
>Echinococcus_multilocularis_CDI70288
WLEDMFSANAIAVVDFAITLRIIMNCEDEKINTLVLYGPTNTGKSLICKLMTSFLEH-GSVMRRQEASAFAYENLLNRKVALMEEPKICAANQQDLKQILGGEPFEVHIKYQNPDLLERLPVVVTTNEPLGVRLSDVDAAATEGRCKSYTLDKQIC
>Echinococcus_multilocularis_CDS35386
WLEDMFSANAIAVVDFAITLRIIMNCEDEKINTLVLYGPTNTGKSLICKLMTSFLEH-GSVMRRQEASAFAYENLLNRKVALMEEPKICAANQQDLKQILGGEPFEVHIKYQNPDLLERLPVVVTTNEPLGVRLSDVDAAATEGRCKSYTLDKQIC
>Echinococcus_multilocularis_CDI70556
WLEDMFSANAIAVVDFAITLRIIMNCEDEKINTLVLYGPTNTGKSLICKLMTSFLEH-GSVMRRQEASAFAYENLLNRKVALMEEPKICAANQQDLKQILGGEPFEVHTKYQNPDLLERLPVVVTTNEPLGVRLSDVDAAATEGRCKIYTLDKQIC
>Echinococcus_multilocularis_CDS36689
WLEDMFSANAIAVVDFAITLRIIMNCEDEKINTLVLYGPTNTGKSLICKLMTSFLEH-GSVMRRQEASAFAYENLLNRKVALMEEPKICAANQQDLKQILGGEPFEVHTKYQNPDLLERLPVVVTTNEPLGVRLSDVDAAATEGRCKIYTLDKQIC
>Echinococcus_multilocularis_U6FUW9
WLEDMFSANAIAVVDFAITLRIIMNCEDEKINTLVLYGPTNTGKSLICKLMTSFLEH-GSVMRRQEASAFAYENLLNRKVALMEEPKICAANQQDLKQILGGEPFEVHTKYQNPDLLERLPVVVTTNEPLGVRLSDVDAAATEGRCKIYTLDKQIC
>Echinococcus_multilocularis_CDS35660
WLEDMFSANAIAVVDFAITLRIIMNCEDEKINTLVLYGPTNTGKSLICKLMTSFLEH-GSVMRRQEASAFAYENLLNRKVALMEEPKICAANQQDLKQILGGEPFEVHTKYQNPDLLERLPVVVTTNEPLGVRLSDVDAAATEGRCKIYTLDKQIC
>Echinococcus_multilocularis_U6HA06
WLEDMFSANAIAVVDFAITLRIIMNCEDEKINTLVLYGPTNTGKSLICKLMTSFLEH-GSVMRRQEASAFAYENLLNRKVALMEEPKICAANQQDLKQILGGEPFEVHTKYQNPDLLERLPVVVTTNEPLGVRLSDVDAAATEGRCKIYTLDKQIC
>Echinococcus_multilocularis_U6H9Z7
-----FSANAIAVVDFAITLRIIMNCEDEKINTLVLYGPTNTGKSLICKLMTSFLEH-GSVMRRQEASAFAYENLLNRKVALMEEPKICAANQQDLKQILGGEPFEVHIKYQNPDLLERLPVVVTTNEPLGVRLSDVDAAATEGRCKSYTLDKQIC
>Echinococcus_multilocularis_U6H948
--------------DFAITLRIIMNCEDEKINTLVLYGPTNTGKSLICKLMTSFLEH-GSVMRRQEASAFAYENLLNRKVALMEEPKICAANQQDLKQILGGEPFEVHIKYQNPDLLERLPVVVTTNEPLGVRLSDVDAAATEGRCKIYTLDKQIC
>Echinococcus_multilocularis_U6HBN8
WLEDMFSANAIAVVDFAITLRIIMNCEDEKINTLVLYGPTNTGKSLICKLMTSFLEH-GSVMRRQEASAFAYENLLNRKVALMEEPKICAANQQDLKQILGGEPFEVHIKYQNPDLLERLPVVVTTNEPLGVRLSDVDAAATEGRCKIYTLDKQIC
>Echinococcus_multilocularis_U6H9K2
WLEDMFSANAIAVVDFAITLRIIMNCEDEKINTLVLYGPTNTGKSLICKLMTSFLEH-GSVMRRQEASAFAYENLLNRKVALMEEPKICAANQQDLKQILGGEPFEVHIKYQNPDLLERLPVVVTTNEPLGVRLSDVDAAATEGRCKIYTLDKQIC
>Echinococcus_multilocularis_U6HGG3
WLEDMFSANAIAVVDFAITLRIIMNCEDEKINTLVLYGPTNTGKSLICKLMTSFLEH-GSVMRRQEASAFAYENLLNRKVALMEEPKICAANQQDLKQILGGEPFEVHIKYQNPDLLERLPVVVTTNEPLGVRLSDVDAAATEGRCKIYTLDKQIC
>Echinococcus_multilocularis_U6HBG0
WLEDMFSANAIAVVDFAITLRIIMNCEDEKINTLVLYGPTNTGKSLICKLMTSFLEH-GSVMRRQEASAFAYENLLNRKVALMEEPKICAANQQDLKQILGGEPFEVHIKYQNPDLLERLPVVVTTNEPLGVRLSDVDAAATEGRCKIYTLDKQIC
>Echinococcus_multilocularis_U6HD97

```
-----FSANAIAVVDFAITLRIIMNCEDEKINTLVLYGPTNTGKSLICKLMTSFLEH-GSVMRRQEASAFAYENLLNRKVALMEEPKICAANQQDLKQILGGEPFEVHIKYQNPDLLERLPVVVTTNEPLGVRLSDVDAAATEGRCKIYTLDKQIC
>Echinococcus_multilocularis_U6H9K6
-----FSANAIAVVDFAITLRIIMNCEDEKINTLVLYGPTNTGKSLICKLMTSFLEH-GSVMRRQEASAFAYENLLNRKVALMEEPKICAANQQDLKQILGGEPFEVHIKYQNPDLLERLPVVVTTNEPLGVRLSDVDAAATEGRCKIYTLDKQIC
>Echinococcus_multilocularis_CDI70555
WLEDMFSANAIAVVDFAITLRIIMNCEDEKINTLVLYGPTNTGKSLICKLMTSFLEH-GSVMRRQEASAFAYENLLNRKVALMEEPKICAANQQDLKQILGGEPF---------------------------------------E-----------
>Echinococcus_multilocularis_CDS35653
WLEDMFSANAIAVVDFAITLRIIMNCEDEKINTLVLYGPTNTGKSLICKLMTSFLEH-GSVMRRQEASAFAYENLLNRKVALMEEPKICAANQQDLKQILGGEPF---------------------------------------E-----------
>Echinococcus_multilocularis_CDI70553
WLEDMFSANAIAVVDFAITLRIIMNCEDEKINTLVLYGPTNTGKSLICKLMTSFLEH-GSVMRRQEASAFAYENLLNGKVALMEEPKICAANQQDLKQILGGEPFEVPVKYQNPDLLERLPVVVTTNEPLGVRLSDVDAAATEGRCKIYTLDKQIC
>Echinococcus_multilocularis_CDS35651
WLEDMFSANAIAVVDFAITLRIIMNCEDEKINTLVLYGPTNTGKSLICKLMTSFLEH-GSVMRRQEASAFAYENLLNGKVALMEEPKICAANQQDLKQILGGEPFEVPVKYQNPDLLERLPVVVTTNEPLGVRLSDVDAAATEGRCKIYTLDKQIC
>Echinococcus_multilocularis_U6HA02
-------MN----------------CEDEKINTLVLYGPTNTGKSLICKLMTSFLEH-GSVMRRQEASAFAYENLLNGKVALMEEPKICAANQQDLKQILGGEPFEVPVKYQNPDLLERLPVVVTTNEPLGVRLSDVDAAATEGRCKIYTLDKQIC
>Hydatigera_taeniaeformis_LL723621
-------------------------MHM------------------------------------------------DRTVAVLEEPRMNAPNVNDMKQLFGGEPFEINVKYKPMQYLNRLPVIVTTNECIGSIKTHIDCAALESRYCQFNFSLADS
>Hymenolepis_microstoma_CDJ13946
-------------MQYLQRLPVIITNEY--LGCRLPDVDAAAGKSLLCKVMTEFLLT-GTISRQSENTNFAFENLLDRSVAILEEPKINASNANDMKQLLGGESFEVAVKYKPMQFLPRLPVIITTNEYLGCRLPDVDAAALESRYYQFTFSTQIA
>Hymenolepis_microstoma_CDS32661
-------------MQYLQRLPVIITNEY--LGCRLPDVDAAAGKSLLCKVMTEFLLT-GTISRQSENTNFAFENLLDRSVAILEEPKINASNANDMKQLLGGESFEVAVKYKPMQFLPRLPVIITTNEYLGCRLPDVDAAALESRYYQFTFSTQIA
>Hydatigera_taeniaeformis_LL725419
WINNLFHSNGISPHAFCRKMECIMDKKDDKVNTLVLYGPTNTGKSLLCKLMTEFLLT-GTISRRSENTAFAFENLLDRSVGILEEPKINSANMNDMKQLLGGEEFEVAVKYKPMQ-----------------------------------------
>Hymenolepis_diminuta_LM390786
WLDNLFDANGIIPSEFINKLDRVMNRNDSKINSIVLYGPTNTGKSLLCKIMTEFLLT-GTIIRRSENSNFAYENLLDRSVAILEEPKINAANVNDMKQLLGGESFEVAVKYKPMQFLHRIPVIITTNEYLGCRIPDVDAAALESRYFQYTFSSQIA
>Hymenolepis_diminuta_LM388960
-LDCLFQSNGIVALEFCNQLECVMNKRDNKVNTIVLYGPTNIGKSLVCKIATEFLLT-GTISRRSENTAFAFENLLDRSVAILEEPKINAGNANEMKQLLGGESFEVSVKYKPMQYLPRLPVIITTNEYLGCRLPDVDAAALESRYHQFNFATQIA
>Hymenolepis_microstoma_CDS32658
WLECLFKSNGIEPLEFVSRLERVMNKVDNKVNCIVLYGPTNTGKSLLCKIMTEFLLT-GTINRRSENSNFAFENLLDRSVAILEEPKINAANANDMKQLLGGEAFEVSVKYKPMQYLQRLPVIVTTNEYLGCRLPDVDAAALESRYYQYTFSTQIA
>Hymenolepis_microstoma_CDJ13943
WLECLFKSNGIEPLEFVSRLERVMNKVDNKVNCIVLYGPTNTGKSLLCKIMTEFLLT-GTINRRSENSNFAFENLLDRSVAILEEPKINAANANDMKQLLGGEAFEVSVKYKPMQYLQRLPVIVTTNEYLGCRLPDVDAAALESRYYQYTFSTQIA
>Hymenolepis_diminuta_LM389152
WLNQIFHANGIMPFEFCRQVERVLNRQDNKVNTIVLYGPTNTGKSLLCKIMTEFMLT-GTISRRSENTAFAFENLLDRSVAILEEPRINTGNANDMKQLLGGESFEVAVKYKPMQYLLRLPVIITTNEYLGCRLPDVDAD----------------
>Hymenolepis_diminuta_LM390089
WLDTLFRSNGIVPVEFCKTVEKVLDKKDNKVNTIVLYGPTNTGKSLLCKLMTEFLLT-GTISRRSENTVFAFENLLDRTVAILEEPRINAANANDMKQLLGGEAFEVAVKYKPMQYLNRLPVVITTNEYLGCRLPDVDAAALESRYYQFTFSSQIA
>Hymenolepis_diminuta_LM389639
WLETLFRSNGIVPIEFCKMVEKVLDKKDNKVNTIVLYGPTNTGKSLLCKLMTEFLLT-GTISRRSENTVFAFENLLDRTVAILEEPRINAANANDMKQLLGGEAFEVAVMYKPMQYLNRLPVVITTNE-----YLGCRLPDVDARYYQFTFSSQIA
>Hymenolepis_microstoma_CDS30830
---------KWNL---------------LTSCLLWGEGEEEVGGE----HADQHVHTGGIVCQRGEQKAFHFENLLNRTVALIEEPLITIETKNDYKCLLGGERLEIDIKCGARRFLQRVPVIVTTNEDLGSQLTSLA------------------
>Hymenolepis_microstoma_CDJ12115
---------KWNL---------------LTSCLLWGEGEEEVGGE----HADQHVHTGGIVCQRGEQKAFHFENLLNRTVALIEEPLITIETKNDYKCLLGGERLEIDIKCGARRFLQRVPVIVTTNEDLGSQLTSLA------------------
>Hymenolepis_microstoma_CDS30832
-------------RQLVRNVYRIIQ--------------------QFKHQVL------GIVCQRGEQKAFHFENLLNRTVALIEEPLITIETKNDYKCLLGGERLEVDIKCGARRFLQRVPVIVTTNEDLGSQLTSMDKAALYSRV----------
>Hymenolepis_nana_LM406075
WLIAMLNTNSIDIGALLGDIVQIMDKRKAKINTLCFRGQTNTGKTLLANLLTSHLLVRLHLKYS-----FHFFVFAHKTLILVL-------------ALFN--------RLERCADVVRPPFISTT------------------------------
>Hymenolepis_nana_LM409198
WLIAMLNANSIDIGALLGDIVQIMDKRKTKINTLCFRGQTNTGKTLLANLVTSHLLVRLHLKYP---SHFF---VFCKTLILVL---------------------ALFYRLERCAVVVRPPFIS--------------------------------
>Hymenolepis_diminuta_LM389526
WLEFMFNMNHIPVYALLTDIIKIMDMKVNKVNCLCFYGQTNTGKTLLANLITSHLTV-------SDHNLFYFCNIF--------------------------------------------------------------------------------
>Hymenolepis_diminuta_LM391469
WLMMLLNQNGINIKELLADIITIMDKKTTKVNTLCFKGQTNTGKTLLANLITSHLTV-----RN-------IDNLI---------------------------SY---------------------------------------------------
>Hymenolepis_microstoma_CDS33272
WLMMMLNQSGININELLTDIINIIYTKTTKMDTFCFKGQTNTGKTLLANLIASHLIL-GPVCRRGDQTAFHFDNLPNRTVALMGKPRITMITKNDY-VSLE----EVDLKS---------------------------------------------
>Hymenolepis_microstoma_CDJ14557
WLMMMLNQSGININELLTDIINIIYTKTTKMDTFCFKGQTNTGKTLLANLIASHLIL-GPVCRRGDQTAFHFDNLPNRTVALMGKPRITMITKNDY-VSLE----EVDLKS---------------------------------------------
>Hymenolepis_microstoma_CDS30612
---MLLNQNGISINELLTDIINIMDKRSTKMNTLCFKGQTNAGKTLCANLITSHLTL-GTVCRRGYQTAFHFDNLLNRTAALMEEPRIAMITLEEVD--------LKAMSIDRAALYS--------------------------GVKQHTLNEPSS
>Hymenolepis_microstoma_CDJ11897
---MLLNQNGISINELLTDIINIMDKRSTKMNTLCFKGQTNAGKTLCANLITSHLTL-GTVCRRGYQTAFHFDNLLNRTAALMEEPRIAMITLEEVD--------LKAMSIDRAALYS--------------------------GVKQHTLNEPSS
>Hymenolepis_microstoma_CDJ13509
------------------------------MNTLCFKGQTNTGKTLLANPITSHLTL-ATACCRGDQTAFHFDNLLNRTVALMEESRNTMITRNDYKCLLGGGRFKIDVNYGAREV----------------------------------------
>Hymenolepis_microstoma_CDI96352
------------------------------MNTLCFKVQTNTGKTLLANLITSHLTL-GTVCRRGYQTAFHFDNLVNRTVALMEELRITMITRNDYKCLLGGGRFESHVKYGARRVCN-----------------------VFRGVKQHTLNEPSA
>Hymenolepis_microstoma_CDS35108
WLMMMLSQHGIDIDELLKDIAYIMDKKTTKVNSLCFKGQTNTGKKLLANFITPHLTL-GTVCRRGDRTAFHFDNLLNRTVTLMEEPKITTTTKSAYKCLLGGDRFEIDVNYGARRFLQRIPVVDTTNEDVGALLTSIDRAALYSRVKQYTLNEQDI
>Hymenolepis_microstoma_CDI96179
WLMMMLSQHGIDIDELLKDIAYIMDKKTTKVNSLCFKGQTNTGKKLLANFITPHLTL-GTVCRRGDRTAFHFDNLLNRTVTLMEEPKITTTTKSAYKCLLGGDRFEIDVNYGARRFLQRIPVVDTTNEDVGALLTSIDRAALYSRVKQYTLNEQDI
>Hymenolepis_microstoma_CDS34279
WLMMMLSQHGIDIDELLKDIAYIMDKKTTKVNSLCFKGQTNTGKKLLANFITPHLTL-GTVCRRGDRTAFHFDNLLNRTVTLMEEPKITTTTKSAYKCLLGGDRFEIDVNYGARRFLQRIPVVDTTNEDVGALLTSIDRAALYSRVKQYTLNEQDI
>Hymenolepis_microstoma_CDJ15564
WLMMMLSQHGIDIDELLKDIAYIMDKKTTKVNSLCFKGQTNTGKKLLANFITPHLTL-GTVCRRGDRTAFHFDNLLNRTVTLMEEPKITTTTKSAYKCLLGGDRFEIDVNYGARRFLQRIPVVDTTNEDVGALLTSIDRAALYSRVKQYTLNEQDI
>Hymenolepis_microstoma_CDS30831
WLTLMLNQNGIDIYELLNDIIAIMEKKTTKANTLCFKSQTNTGKTLLANLITSHLTV-GTVCRRGDQTAFHFDNLLNRTAALMEEPRITMTTKNDYKCLFGGDRFEIDVKYGARRFLQRIPVVDTTNEDLGALLALVDRAALYSRVKQYNLNEQIS
>Hymenolepis_microstoma_CDJ12116
WLTLMLNQNGIDIYELLNDIIAIMEKKTTKANTLCFKSQTNTGKTLLANLITSHLTV-GTVCRRGDQTAFHFDNLLNRTAALMEEPRITMTTKNDYKCLFGGDRFEIDVKYGARRFLQRIPVVDTTNEDLGALLALVDRAALYSRVKQYNLNEQIS
```

Figure S2

```
>Fundulus_grandis_JW661431
SSEQNPIVRDILHVMHAYSALSLQEMVTKAPHIMSKHLHRPSLQTIIQNCRLFYMNSSRKANLLVDLPDGRPDPTRNQVLVTQHEQFAHDFTLWCTKRSSKRNTLVLHGPSNTGKTSFIRPLLALYTFSFSGCSRDLIVWENNIDLCKLMFEGSTTELPHKFRDPIKTDRTPILITTNRECAAIQNRCYIY
>Fundulus_grandis_JW661434
SSEQNPIVRDILHVMHAYSALSLQEMVTKAPHIMSKHLHRPSLQTIIQNCRLFYMNSSRKANLLVDLPDGRPDPTRNQVLVTQHEQFAHDFTLWCTKRSSKRNTLVLHGPSNTGKTSFIRALYAEATFSFSGCSRDLIVWENNIDLCKLMFEGSTTELPHKFRDPIKTDRTPILITTNRECAAIQNRCYIY
>Diaphorina_citri_XP_008482940
--------MQYALHELWKITPGYKRYKWLRMQYALHVYHHKRGPARISNEGDLSIIQHTQYRGTVFIIAEHGDHWHCTYTSSQCVKAVTRCFGIRI-KRRTIRSLDFN------TSYATNVIYGEYGPGVGDLSGKSAESGTVENEMRIFLGCTDRGEGAAEGSGTPLSNQPTHKRRKGEAHPCAPVLSTR
>Drosophila_persimilis_XP_008482940
-------------------VQKANQESWKSKR------CKFDNSKSDVPSGRRTFLTAGMCNTSLDTTISIGSECDDNFISSTEDIQCEEMFTTID-GHTLIRTKAVSSPEE--REVFVE------EFSERNLKQRIVHDVNRQRDFRM----DYEQLCRKFTGTI------LIIASHGHDCTYSNRCSFY
>Hymenolepis_microstoma_CDS25721
-------------------------------------------------MSVHFETAYNSTKTEHEFSCPLRQAQPLPTFGSHHTRHCANVC-----KHPHHEFVCA-GVGN--EAIRVT---GAVAWYHQRCDSTGLLFCAYANCVRLHAAAAVGAIEVSL----QVVVKAMVVFVSDDGDLFVC-----
>Feline_bocavirus_JQ692585
MSKKESLILDCLKRCEDNLWLTYEDLVGGCADLILMLESMPGGSKLIESVLNMLHVRITQTHSALSYLHVRYDMKEADSLHANKLQVGHWICCVLHKKAGKQNTLNFFGPASTGKTNLAKAIVGCVNFIFNDCAKLVVWWEDWVEQAKCILGGTEFRIDRKHRESHLLPQTPVIISTNNHEAPLRERVVQF
>Canine_minute_virus_FJ214110
ITKRETLMIDCMNRCEQNYWLTYEDLVNGCADLVVMLECQSGGNKLIEQILSMVHIKMTQKYTALSYILAKYDPIPWKLLTLQGWQFGHWLLCVLDKKAGKQNTINFYGPASTGKTNLAKAIVGCVNFVFNDCTKLICWWEDWVEQAKCIMGGTQFRIDRKHKDSHLLPQTPLIISTNHHSMPLRERVVQF
>California_sea_lion_bocavirus_1_JN420361
MTKREMLMLDCIDRCEKDNLLTYEDLVDNCSALLVMLESQPGGGRLIENLLQMVHIRICQKHTALSYIQLRYSDEPYQLFAIQGWQAGHWLCCVLNKTAGKQNTVNFYGPASTGKTNMAKAIVGCVNFLFNDCAKLVCWWEEWVEQSKCILGGTEFRIDRKHKDSMLLPQTPVMISTNNHSKPLREQFNFM
>Canine_bocavirus_1_JN648103
ITKKETLMIDCIDRCEKNHLLTYEDLVNECSDLVIMLGSQPGGTKLIETLLQMVHIKICQKYTALSYVLSRYSAIEIQLLIFQGWQVGHWLCCVLHKTAGKQNTVCFFGPASTGKTNFAKAIVGCVNFVFNDCAKLVNWWEDWVEQAKCLLGGTEFRIDRKHKDSQLLPQTPVVISTNHHAKPLRERIVQF
>Scylla_olivacea_GDRN01091983
GNERPPTPPTPRCFKRRRDASTSPDRVRKSKNTAHSPEDDSPEEETTRPTATTSGQNTTRKLGRTTNNKPRGGQRK--------KKNGPKTIDLCERSTRGQDQMDARSEAN--QQVSFY------TFIVRERPPRQGGRADHGDHWHITFASNRTNLNRKRKTICLTSGARVEASASTTLIKMLK-----
>Human_parvovirus_B19
GTKASIKFQTMVNWLCENRVFTEDKWKLVDFNQYTLLSSSHSGSFQIQSALKLAIYKATNLVPTSTFLLHTDFEQVVKLLLCQNQHVLKWIDK----KCGKKNTLWFYGPPSTGKTNLAMAIAGMVNFPFNDVAKSLVVWDTIVEAAKAILGGQPTRVDQKMRGSVAVPGVPVVITSNGERMVKLNRCSPD
>Bufavirus_1_JX027296
VSAKEKSIKETVDSLTDKRIITREKWMLQDPDSYIQFIAQPGGEQTCSAVLDIATLRIATEKSAYDLVKEHPGKLKWKLIKNNNQKVYHAIMCCLNKQMGKRNTILLCGPASTGKSLLAQKIAGCYNFPFNDCVKNLIWIETQVNSFKAIMSGQAIRLDQKGKGSKSIEPTPVVMTTNEHRQPIMDRCLRF
>Bactrocera_dorsalis_GAKP01023238
RNLVGSLLSHESSTTFTEATVTMETSSWILNNYAENFQ-GPSCSSRIMETKSTLSTTAVIPITPVDVHSTISSQTSEDMLEESFRSSTGSICQSIC-RRTQGCTYTSTSPGE--RGFQVV---DVYDFA-KICKPKTEWWK------TFLLQS---PVDPNY----QLVMKLLLVVSNQKEERETK-----
>Bactrocera_dorsalis_GAKP01023412
----------------------------------------MWTPDFLLHAISTLGKMLPLALVKKEYEA---------------KRVIHTLMEYLLVQKQLLSQKKELGKSS--MRNLVESLESSTTFTTVTMETSSWILNSYAENFQGPSCSRSMATSLS-----MTAVIPTTLVDVHSTMSSQT-----
>Octopus_bimaculoides_KOF81493
------------------------------------------------------------------------------------HTFVNNVWYLLNKKHPKCNLLVLRGLPNTGKSMIARSIASTISFPFQNLIVEIGLIEETLQTFKKLAEGTDIKVSVKFKPDQIVHRTPLIITSNNERVAFITRYTE-
>Lamellibrachia_sp._x2
------------------------------------------------------------------------------------MELASASCGLIE------------------EPSFTD---------------------EALQSFKKLAEGTPTEVSVKNKAAVKIARLPLFVTANYEKQAFSTRMIE-
>Lamellibrachia_sp._x9
------------------------------------------------------------------------------------MELASASCGLIE------------------EPSFTD---------------------EALQSFKKLAEGTPTEVSVKNKAAVKIARLPLFVTANYEKQAFSTRMIE-
>Lamellibrachia_sp._x4
------------------------------------------------------------------------------------------------------------------MYKYPATVQGTTSFPFMELACSLGLIEEALQTFKKLAEGTQTEVSVKNKGAARVPRIPLFVTANYERQAFASRMIE-
>Lamellibrachia_sp._x6
------------------------------------------------------------------------------------------------------------------------TIQGTTSFPFMELASSLGLIEETLQTFKKLAEGTPTEVSVKNKGAARVGRIPLFITANYERQAFASRMIE-
>variole_D5_NC_001611
GNKLFNIAQRILDTNSVLLTERGDHIVWINNSWKFNSEITKLILSIRHQLPKEYSSELLCPRKRKTVEANIRDMLVSTGFKFDDINIINDIQPLTDNATKGCLTFFFGGKSTT-KRLLKSDLFGPNVFCLPDFARSDNIKKNNRNHATIIIDTNYKPVRIDN---ALMRRIAVVRFRTHDKVKLLDDGKIQ
>Aretaon_asperrimus_GAWC01079972
STDQ?PICIPYRNLAFW?Q??D?K??N?YDFRGNFFKF?GPSTGIIFHGAKLSISNQATLRQRLLQQ?QTTTTTWDQNLI?AKGPPF?RSDYSFMP-NY?KQDLLTYEGHT---KHMSFHPKMGKYEFQSNNVHKFLIPGRANDSELQDLTVGTYYEP----VELTHYINRPLLLIGPPSENGLMKRLKIQ
>Amblyomma_americanum_GAGD01002820
---------------------------------------------------------------------------------------------MME-NKKSTEEETFYGIKE--SDYIIS---GRSATPSQNNSRSTPHSTKHRDELRRALAT-----QRDYETAQ-------------DEVAKNR-----
>Rhipicephalus_appendiculatus_CD793420
--------------------------------------------------------------------------------------VLASGCYLFN-EDWGSSGRCIA------ASHLVA---------KPKDSYELSYWN--FEDFCHHYD-------------MVQPNPTFLAMTT-------------
>Hymenolepis_microstoma_CDS33100
PTKTVRVNVYLLLRYFKSRGQVREAGYELREEWEMAQELTALEPNQSCRSDIRPSRTKSLVAQRSDDRKARN------------TPLERWTIERWN-RHKHAQELFMQIKDE--KESLTQ---------FRDSLRAIRLHSDKMLSLNDIKGS-----IPRYDTRKPYTRSETATERKSNDASYEPAIR--
>Trichosurus_vulpecula_EC348320
-----QTITSTICNLYNKRIVSIENWMVDEPESYIQHIANPGGEIIIKTIIEMVLLRMAKYMTAYEVIAEKGKITKFKILKMNNIKVTHAVMCVLNKKLGKRNTILLSGPASTGKSLITQNICGCYNFPFNDCCKKHHMDR-------------EPINVRTKHQTRPKGKRKQ-INRTNSSNNDIK------
>Porcine_parvovirus_PT4_PPU44978
QTKKEVSIKCTIRDLVNKRCTSIEDWMMTDPDSYIEMMAQTGGENLIKNTLEITTLTLARTKTAYDLILEKAKPSMCKIFSMHNIKVCHAITCVLNRQGGKRNTILFHGPASTGKSIIAQHIAGCYNFPFNDCTKNLIWIENQVNQFKAICSGQTIRIDQKGKGSKQIEPTPVIMTTNEHTQPIRDNINLT
>Canine_parvovirus_M19296
QTKKEVSIKCTLRDLVSKRVTSPEDWMMLQPDSYIEMMAQPGGENLLKNTLEICTLTLARTKTAFELILEKADNTKCQIFRMHGIKVCHAIACVLNRQGGKRNTVLFHGPASTGKSIIAQAIAGCYNFPFNDCTKNLIWIEQQVNQFKAICSGQTIRIDQKGKGSKQIEPTPVIMTTNEHTQPIRDNIKLV
>Rat_parvovirus_NTU1_AF036710
QTKKEVSIKCTLRDLVAKRVTSPEDWMMQQPDSYIEMMAQPGGENLLKNTLEICTLTLARTKTAFDLILEKAEPSKCRIFAGHGIKVCHAIACVLNRQGGKRNTVLFHGPASTGKSIIAQAIAGCYNFPFNDCTKNLIWVEQQVNQFKAICSGQTIRIDQKGKGSKQIEPTPVIMTTNEHTQPIRDNIHLT
>Mouse_parvovirus_1_MPU12469
QTKKEVSIKTTLKELVHKRVTSPEDWMMMQPDSYIEMMAQPGGENLLKNTLEICTLTLARTKTAFDLILEKAETSKCKIFAFHGIKVCHAICCVLNRQGGKRNTVLFHGPASTGKSIIAQAIAGCYNFPFNDCTKNLIWVEQQVNQFKAICSGQTIRIDQKGKGSKQIEPTPVIMTTNEHTQPIRDNIHLT
>Mouse_parvovirus_3_NC_008185
QTKKEVSIKTTLKELVHKRVTSPEDWMMMQPDSYIEMMAQPGGENLLKNTLEICTLTLARTKTAFDLILEKAETSKCKIFAFHGIKVCHAICCVLNRQGGKRNTVLFHGPASTGKSIIAQAIAGCYNFPFNDCTKNLIWVEQQVNQFKAICSGQTIRIDQKGKGSKQIEPTPVIMTTNEHTQPIRDNIHLT
>Gray_fox_amdovirus_JN202450
DEQCSDMADDSQDWDDSQEPPKTEPPKVTTETISNVTYTSTIWASCSTKATKPKTITETAKQPSKKLTTAKSTLDSECIMKFNDEKVLKNIIATVLKQSGKRGCIWFYGPGGTGKTLLANLICGMVNFPWTDCGRNMIWLENFIEDFKAITGGGDIKVDTKNKQPQAIKGVTVITSNKDHSEPLKQRIVKI
>Aleutian_mink_disease__virus_JN040434
MYLDEQSSDIMDANIDWEDGQDAPKVTDQTDSATTKTGTSLIWKSCATKVTSKKEVANPVQQPSKKLYSAQSTLDADCIIKFNEEQYLKKVLCTILKQGGKRGCIWFYGPGGTGKTLLASLICGMVNFPWTDCGRNIIWAENWVEDFKAITGGGDVKVDTKNKQPQSIKGCVIVTSNTNHAEPLKQRMIKI
>Acheta_domesticus_mini_ambidensovirus__KF275669
ISRAGQLIHFLLNNVYSPLDLIKQSNEWYTSPFATCKETGVEGAHTFLKSLSVSEMENFVKGNHIQYKNNIVRYSCEQLLMYQFSDFIHQLYYVLNKQNGKKNCIELIGPPSSYKSTFLHWVAGIIPFGFAPAVKRVLIIDEYHETLLNILSGTTCNVNVKYKTNGYVTHTPVLMASNYTSTRFEHRMVTF
>Aretaon_asperrimus_GAWC01012962
PSQRRYSLEELLSMKLTPALIAKKNLCFVCTPLMLLTKLVNLNKTSIQQGLETAKCLINSFTCNELVSYVQGTHPISQLLSYQFLYFKRCFFDVIT-VADKKNVLEIVSLPSAYKTTFITMVAGYCRFGFQNIITRLAIIDPSKDILLCLFSGDKINLNQKNLGDDTAVKTSCVVLSNTIGQRWNDRMYRF
>Extatosoma_tiaratum_GAWG01046839
--------------------------------------------------------------------------------------MVKLYEVVIG-INNLKNVVEIISPPSAYKSTFATMIAGYCRFGFQNIAVRLAVIDAGKETLLCLFSGDKVNVARKNLPDHVQLNTPFLVLSNR----WDVRMERF
>Penaeus_merguiensis_hepandensovirus_DQ458781
YLENLQVLEKYLVKHKCYTIQDFKMMQRSDDEIWVNYMYDIQNLEKVIEKLNIMEYSLQQADYIEGNTWIGEDLWNKIIENMRVVQWIQDFMDIMHGNLPKINCMMLYGNSNSGKTQLIDALTGLVTFHFSNITMSTIVVGQTIEQWKGLCGGENVTMPMKYKEHKHMFRKPVFLTNQHDRRAIENRSFMY
>Penaeus_chinensis_hepandensovirus_AY008257
YLENLQVLEKYLVKHKCYTIQDFKMMQRNEDEIWVNYMYDIQNLEKVIEKLNIMEYSLQQADYIEGNTWIGEDLWNNIIENMKVVQWIQDFMDIMHGNLPKINCMMLYGNSNSGKTQLIEALTGLVTFHFSNITMSTIVVGQTIEQWKGLCGGENVTMPMKYEHKTHMFRKPVFLTNQHDRRAIENRSFMY
>Fenneropenaeus_chinensis_hepandensovirus_JN082231
YLENLQVLEKYLVKHKCYTIQDFKMMQRNEDEIWVNYMYDIQNLEKVIEKLNIMEYSLQQADYIEGNTWIGEDLWNNIIENMKVVQWIQDFMDIMHGNLPKINCMMLYGNSNSGKTQLIEALTGLVTFHFSNITMGTIVVGQTIEQWKGLCGGENVTMPMKYEHKTHMFRKPVFLTNQHDRRAIENRSFMY
>Penaeus_monodon_hepandensovirus_2_EU247528
YLENLQVLEKYLIKHKCYTIQDFKMMQKNEDEIWVNYMYDIQNLEKVIEKLNIMEYSLQQADYIEGNTWIGEDLWNKIIQNMKVVQWIQDFMDIMHGNLPKVNCMMLYGNSNSGKTQLIEALTGLVTFHFSNITMSTIVVGQTIEQWKGLCGGENVTMPMKYHKT-HMFRKPVFLTNQHDRRAIENRSFMY
>Penaeus_monodon_hepandensovirus_3_EU588991
YLENLQVLEKYLIKHKCYTIQDFKMMQRNEDEIWVNYMYDIQNLEKVIEKLNIMEYSLQQADYIEGNTWIGEDLWNKIIQNMKVVQWIQDFMDIMHGNLPKVNCMMLYGNSNSGKTQLIEALTGLVTFHFSNITMSTIVVGQTIEQWKGLCGGENVTMPMKYHKT-HMFRKPVFLTNQHDRRAIENRSFMY
>Penaeus_monodon_hepandensovirus_1_DQ002873
YLENLQVLEKYLVKHKCYTIQDFKMMQRSDDEIWVNYMYDIQNLEKVIEKLNIMEYSLQQADYIEGNTWIGEDLWNKIIENMKVVQWIQDFMDIIHGNLPKINCMMLYGNSNSGKTQLIEALTGLITFHFSNITMSTIVVGQTIEQWKGLCGGENITMPMKYKEHKHMFRKPVFLTNQHDRKAIENRCFMY
>Penaeus_monodon_hepandensovirus_4__FJ410797
YLENLQVLEKYLRKHKCYTIQDFKMMQRNDDEIWVNYMYEIQNLEKVIEKLNIMEYSLQQADYIEGNPWIGEDLWNENMRVESRVQWIQDFMDIMHGNLPKINCMMLYGNSNSGKTQLIEALTGLVTFHFSNITMSTIVVGQTIEQWKGLCGGENVTMPMKYKEHKHMFRKPVFLTNQHDRKAIENRCFMY
>Daphnia_pulex_EFX61411
DFRLEEEQKVNSVSSACNFLREISKRYWLSDFSSLLAVSDVMLKEDLQKVLMYNMLKVQQYMNNVFTLEYNIMRNQFSTVRMNDILFRKWFDR----ELGKKNTIILIGEPSCGKTWLAK---GKIQFIFSGCCGRIILHDAYSETLKKIYAGECCPISVKYQSGQLSCGAPVIGTCNSFRQAFDERITFL
>Neobenedenia_melleni_GW918566
EELTNNIILFLIKSVLNLSFHKFDLEIFIKNLDININN--KNILLYIIKNYYDYIKFYYDFYADIQLEPLYLHSFNTKILNEIFNFFISSLKNIIFKKYFKINTLLFYGNPNSGKSLIANQVYKIVEFYLSNFDANIIICEANVDDFKNILGGTSIVVNKKYERRGRINRIPILITSNYDTSALLRRMIV-
>Enchytraeus_crypticus_GALF01015487
LCKRWNAFTVTDMYQAQAEIPKEEDKDWREFWNRMVNK------PSIRTMMDNIRSSVHSDYMYKPFQDLVEEACTRQWLKDQGRDFVQDVVDILDRKLEKINTMCLIGPSNSGNTVMFSATIGRRKFVFQEIPTRLVCVDDNQENWKLLLGGEEMSVPVKFKGNARILRTPVLLTGNKNQDAFMNRMK--
>Planococcus_citri_densovirus_AY032882
EKVVEEVSSLLVKYCPVPLIGIKNLIPPNHADFRTIL-FDPRNKDNYISACDIHALHL-NKYSFKEFHELYSQADNDELLKFQLKPFLSNLRNWFNEGNPKMNALCVIGPPNSGKNYFFDAIIGHINFALQDVVRRIVVGNGAKEDFKKLCEGTAFNVRVKFKADCIFTKTPVLLISNNWDPHFKDRLKTI
>Solenopsis_invicta__densovirus_KC991097
LLNKYFVLPSNHIRDIMLGDSQYNFLFDPSNDKAYMASCEIFQKKFIPLKLKDLRDIFEGSTPVFYANNIDPNLYYDDLLRFQYVELLTNVRDWFNDKNPKMNALCVIGPPNSGKNYFWDMFCGHIQFALQECYRRLVVGNGAKEDFKKLCEGTAFNIRVKYQGDKIFTKAPVLLISNFYDTHFKNRLHTI
>Bemisia_tabaci_EZ941734
---------------------------------------------------------------------------------------------------------------------------------FRNFK----------------TGGDTCNVKVKYCMDHILHRTPVIILSNNKDPAFNSRIIRH
>Lamellibrachia_sp._x7
--------------------------------------------------------------------------------------MYQAVYPWLA-----RDQFHLYVPRVNNNSHWSDPALRMVNITFGDKAMHCCILTVDVATKGATIHSASHKLFVSKRDAHVVYHGP-------NECTDQK-----
>Lamellibrachia_sp._x8
-----------------------------------------------------------LVLLTTPYTQEYREATG--------PTLGSASLKLIS-FINRCDVHVVYGPDTCGKKPWCR------TF-ISKCRGLDTVWANVVHQHKALTKNCQIPSTTKF--PGYLSQSP--------RVTWHM-----
>Argulus_siamensis_JW950843
DKIFKEIRALYAANLVVPIENIVDTQAFMDNSIFWEPNYRLKNEFNQYSLADIRSYLENKVVDPLFTCGWLQTDKYKTLLIHQFKEFLTDVVTIYNNLNPKMNCMIIHGPPNAGKNYFMDTLFGYITFSLSNAYCRVVCANGAIEDFKKLCDG---------SG---------------------------
>Sus_scrofa_EV965154
--------------------------------------------RHRQLRRDRVRVDHQKERQTQHHRAVGSEQH--------LRPGTETGRQLR-RVRQRTDLLLRGPVR--QGHRHL---GRA---ADQSG-------VRGESQTGLRGG---------------------------SDTGAGQ-----
>Triatoma_infestans_GBBI01004972
ASRKEHAVATVYGWLEKHIIIPDHDITSSDKWLSSDFKFITERDGIFQSALKIHRTVLSKLKLNEMIDYINKAEYVHMILSFQMVQFLQFLYKLLNHQTGKMNCLLICGPPSVGKTWFANIIGGYIQFPFQDCPRNLLIFDASLETFKLLFAGTPTPANIKYESQNLINRTPVIVTCNHQNSEYRTRINRF
>Halyomorpha_halys_GBHT01013004
EENIIVPDHDITRHSIWLDHQYFRFITDRSPDFQNALKRTILSNMVLDDLREYIRDKNVYYYPPKETVLTLDESYNQQMTAYQLSEFLHNVYLMLNHRSGKHNCILIKGVPSVGKTWFANLLGGYIQFPFQDCPRNLLIFDSSYENFKLLFAGTPLSANIKYESRNLVNRTPVIVCCNSHNPEFNSRIYRY
>Rhodnius_prolixus_T1I3J1
TDESTDEGTSNVYPITSGIYNIIQRWKGANGSQRRSMATPGSNLVQIRIMPYQQAKTMDPTQRYKCTKKYAQVVLN--------RQLFREMEKVTDTGSGLESNMPLSGPPSVGKTWFANIVGGYIQFPFQDCPRNLQIFDMALENFKLLFAGTPTPANIKYESQNLINRTPVIVTCNRKTPEFQTRIRRF
>Rhodnius_prolixus_T1HE18
-----------------MTAHTPTKAVAAD--------LSTQFNNNIKEVTNNTITDESSDEETSNVYPITSGIHNQRWKGANGRSMATPGASLVQRIMPYQQAKTMD--PT--KRYKCT---KRY---AQDCPRNLLIFDMALENFKLLFAGTPTPANIKYESQNLINRTPVIVTCNRKTPEFQKRIRRI
>Graminella_nigrifrons_GAQX01015625
-----------------------------------------MYECRSKSEINSYYYDMKTSTVAI-----------LRLLYHQFRNFLVTLYSFLNSDEGKVNTILIVGPPNSGKNFFIDPLCGKIAFPWMDGFRRVNKWNYFEENVLDLMQGNMLSCNIKYEAPQPLLKTPLIVMSNNNQERFNIRHIRY
>Acheta_domesticus_densovirus_HQ827781
SKFQTFAEEAKVLFSKLNCIPVRDARIIIDRKHPMYFEYTAAISLYVRDINEMSLNDFYLQTLENTCVYQAGNIDPNELILFQCKELLMNIVAWFNRLNPKVNTVCIIGNHNCGKNYFWDAVCGFLKFALQDCVKRIVIGNGAKEDMKKLCEGCPFNVSVKHQSDGIVARTPVCLISNNDAHFVDVRMKVF
>Raphidia_ariadne_GACX01003907
QTKGQKLVEFFKQYPCTPIDNVRQTVQYLNSEF-----ADVCVTDKLFMRARELYSCYFLNKTVIELYQVFQKSTPRELLLFQFSLFLQNLYRVLNRTNGKKNAIMLVGPANACKSMFANVVAGQITFSLDDCVKRILIWDEYLETIKLLFSGDPCPANVKYEKQSIIHKTPVIITGNTNNDEFNNRLNIY
>Drosophila_sechellia_DK330728
------------------------------------------------------------------------------------------------------------------MSHWNR------SFPLQMCGVRLIFWNSVERTLLRLLGGDALNAAQKNQNDTNISKVPVIVTANHKSDEFKVRIQYH
>Drosophila_sechellia_DK334580
--------------------------------------------------------------------------------------------------------------------------------------------------MLRLLGGDALNAAQKNQNDTNISKVPVIVTANHKSDEFKVRIQYH
>Tetranychus_urticae_T1KBZ7
------------------------------------------------------------------------------------------------------------------------MVAGHVNFPLNDCDRRILIWNSAYDTVKMLTEGDPFPASVKHRNDATILKTAIIFTANNDSLKQID-----
>Metaseiulus_occidentalis_JI999665
----------------------------------------------------MSTFYYSIEESIVILEEFLRYQYG--------FFFCQKLYNITE-LIPKRNCMYILGPTNAGKTWFMN------CVSYLNVG--------------------------------YVKNPV-------KGARFPY-----
>Metaseiulus_occidentalis_JL018010_1
```

```
KVTHQTLMCFILTYLPVPFHAACELPAWLDDEDLSYFYKSDRDYCRAIQQVQRRVATMTLHQIHDLTKDCTS----EELLKFQYFFFLQKLYNITERLIPKRNCMYILGPANAGKTWFINCVAGYVHFPYNDCTRRILIWNSALEDVKMLAGGDPFTCNVKYESNVCVNRTPLFFTANSELPEFKSRIYLE
>Metaseiulus_occidentalis_JL018010_2
KVTHQTLMCFILTYLPVPFHAACELPAWLDDEDLSYFYKSDRDYCRAIQQVQRRVATMTLHQIHDLTKDCTS----EELLKFQYFFFLQKLYNITERLIPKRNCMYILGPANAGKTWFINCVAGYVHFPYNDCTRRILIWNSALEDVKMLAGGDPFTCNVKYESNVCVNRTPLFFTANSELPEFKSRIYLE
>Orchesella_cincta_GAMM01017564_1
KSIPDKIIKFLRLYPVVPLYATPDIREYIESPYRLYRAKSPTFQIAVDILENTINRMTICELAELYHNKHCKPIWSESFLLTQFYHFLTILTSILDKSIPKLNTLIVYSPPNAGKNWFFDSVTGQLNFAYQDLYKRVVVWNEELDMLKMVLGGDRTAIKVKHEKGKQSSRVPVIILTNNDDIAFGTRVRMF
>Orchesella_cincta_GAMM01017564_2
KSIPDKIIKFLRLYPVVPLYATPDIREYIESPYRLYRAKSPTFQIAVDILENTINRMTICELAELYHNKHCKPIWSESFLLTQFYHFLTILTSILDKSIPKLNTLIVYSPPNAGKNWFFDSVTGQLNFAYQDLYKRVVVWNEELDMLKMVLGGDRTAIKVKHEKGKQSSRVPVIILTNNDDIAFGTRVRMF
>Papilio_polyxenes_densovirus_JX110122
GNTVEKFVEQFKLRPTWPLEHILDTKDWLESEWCTYIA----GDKQIQRTFQILMKIT----MNMTFEELRMLYQNEELLDYQLRRFITDLYEILEKKHQKTNTFQIVSPPSAGKNFFIETVLGVISFPLMEAVRRVNYWDDATETLKKLFAGTALKASVKFQKEANVQKTPVIITANRTSEVWDDRIIKY
>Bombyx_mori_densovirus_AY033435
GNPVEKFVEQFKLRPTWPLEHILDTKDWLESEWGTYIA----SDKQIQRTFQILMKIT----MNMTYSDLKMLYQNEELLDFQMKRFITDLYEILEKKHQKTNTFQIVSPPSAGKNFFIETVLGVINFPLMEAVRRVNYWDDATETLKKLFAGTALKATVKFQKEANVQKTPVIITANYTKEVWDDRIIKY
>Hordeum_marinum_iteradensovirus_KM576800
GNSVEKFVEQFKLRPTWPLEHILDTKDWLESEWGTYIA----SDKQIQRTFQILMKIT----MNMTYSELKMLYQNEELLDFQMRKFITDLYEILEKKHQKTNTFQIVSPPSAGKNFFIETVLGVINFPLMEAVRRVNYWDDATETLKKLFAGTALKATVKFQKEANVQKTPVIITANFTKEVWDDRIIKY
>Sibine_fusca_densovirus_JX020762
GNPVEKFVEQFKLRPTWPLEHILDTKDWLESEWGTYIA-------SDKQIQRTFQILMKITMNMTYSELKMLYQNEELLDFQLRKFITDLYEILEKKHQKTNTFQIVSPPSAGKNFFIETVLGVINFPLMEAVRRVNYWDDAVETLKKLFAGTALKATVKFQKEANVQKTPVIITANYTKEVWDDRIIKY
>Casphalia_extranea_densovirus_AF375296
GNPVEKFVEQFKLRPTWPLEHILDTKDWLESEWGTYIASDKQIQRTFQILMKITMNMTYSELKMLYQNEHTMPIWGEELLDYQMRKFITDLYEILEKKHQKTNTFQIVSPPSAGKNFFIETVLGVINFPLMEAVRRVNYWDDATETLKKLFAGTALKATVKFQKEANVQKTPVIITANHTKEVWDDRIIKY
>Danaus_plexippus_plexippus_densovirus_KF963252
GNPVEKFVEQFKLRPTWPLEHILDTKDWLESEWGTYIA----SDKQIQRTFQILMKIT----MNMTYDELKMLYQNEELLDFQMRKFITDLYEILEKKHQKTNTFQIVSPPSAGKNFFIETVLGVINFPLMEAVRRVNYWDDATETLKKLFAGTALKATVKFQKEANVQKTPVIITANYTKEVWDDRIIKY
>Priacma_serrata_GACO01003330
--MSNVLYTRQWMQSKYKFLPDTSVLLRSCFKIINYEIANMSVEELWEYIERIEPQNRLFACHSSSISEVYYSVENEQLLLFQYYVFLYDLYLLLNRSVPKYNCICVIGPPNSGKNFFDAVVHSCINYG------------------------------------------------------------
>Blattella_germanica_densovirus_AY189948
GGATGIILDLLSKCAVCPLTEIVFTKEYLQDPIV----ACKRLDSKEVKDAIDTRASVINTWEREDFVAFYNNPNTTELLTFQMVQFCRNLVDTLECNIPKRNCFVVCSPPSAGKNFFFDGVKGQMQFAYQDCHRRIIIWNREMENLKMLFAGDNLSANVKCKPQANVKRTPVIVLTNSQQTAFNDRVITY
>Blattella_germanica_densovirus-like_virus__JQ320376
RGDTRVILDLIKRYAVCPLSEIVYTREYLENPIAVKRL-----DDRDVKNAIDCHAAIINTWNREDYVKFYEDPNTNKLLDYQCKQFVTDLINVTDMNIPKCNCFLIVSPPSAGKNFLCDAIKGQMTFAYQDCHRRLLIWNRETENLKMLFGGDNLSANVKCKPQANVKRTPVICMSNVNHEAFADRVITY
>Tetramorium_bicarinatum_GASM01014809
RGDTGVILDLIKRYAVCPLSEIVYTCEYLENPIAVKRL-----DDRDVKNAIDCHAAIINTWNREDYVKFYEDPNTNKLLDYQCKQFVTDLINVTDMNIPKCNCFLIVSPPSAGKNFLCDAIKGQMTFAYQDCHRRLLIWNRETENLKMLFGGDNLSANVKCKPQANVKRTPVICMSNVNHEAFADRVITY
>Dysaphis_plantaginea_densovirus_FJ040397
GLDSERSAGGHQGEEKHEDGPGVRVWPKTLDELLVKYPCCPPEAFYNVKEFYNNPHLNHIDDNCKKIRVALRNWCAEELLCYQFSKFLYDVYAIVDKLVPKRNSMCVVSPPSAGKNFFFDAVAGMFNFSWADGAKRLVLWNFHVEKIKELLGGDTTRVHVKYKGDQPLQGPPIFLLTNNNDPAFADRLVTY
>Acyrthosiphon_pisum_J9LI55
YEDLIVQLGRQLANKFVSGIHRYISTVIVTSGTRGVQECLRELDSLIERYPPAHFYIISEHEDHIHISHVCGYSRQSFLVRGAFRRGLRRISRAVDSATDYRNAQRYMGQAQ--GGLLAREMSGSV---QKHKTKKNSWVDTLIGTYRAYWG-IKLMHFIDFEKVHAYNRLKEQVYYDINELLLYQNSENV
>Acyrthosiphon_pisum_J9M820
DKLVGAPSSFEAHFGAKTEDVDFVGLLEQTTCNQEITSADASISLEGSEKMSDYLIDINIYKRKDIWDKPKGDYWRRGTFPANHVTYASKIVTLLGKMMPMKNAQRYMGQAE--GGLLAW------SYSSWDVPRDVCIENNDADEKRKKDTQPGLQSTLAI----LAPPNEGTANKTNNFASMEARLVLW
>Acyrthosiphon_pisum_J9LBV1
------------------------------------------------------------------------------------------------------MEAA-G-------------------KRSVLWNNHVNELKALLGGDSCRI-----SDTDMCNAPNIVHVHIREGVIEE-----
>Acyrthosiphon_pisum_J9LI54
EEHIHIGHICGYTGQTCRCSFLIRGTFWSLYRRRGLRRISRGIDLGPSDYRDILRYLSSGSRRIHCVGGFGENGRL--------KYLSVNLINIIDKRIPKCNSLAILAPPHAGENYFFDAVAGVLNFAFMEAAKRLVLWNNHVNELKSLLGGDSCRISVKYKTDQ-----PI-----------------
>Acyrthosiphon_pisum_J9LDI1
AAKFVSGIHRYVSRGVDGVENCLRELDSLIDRYPPRHFYIISAHGDHVHISHNCGYSGQTCRYPFILKLWRKDIWDDYWRGATAVTYASKRVTLLGKPMKNCNTLAILAPPNAGKNYFFDAVAGCLNFAFMEAAKRLVLWNNHVNELKALLGGDSCRVSVKYKSDQALQGPPIIILTNDGMTAFRPRIKLY
>Acyrthosiphon_pisum_J9LTF9
YTLPTFVDTQAKLTQRHLEQAKGGLLAWSYGEVSRDFPCQPSGSTVRVPNSHINGGENDSVKKSKKNQK-------NTWVDPKNGPVSKNLINIIDKRIPKCNTLAILAPPNAGKNYIFDAVAGVLTINFMEAAKRLVLWNNHVNELKALLGGDSCRISVKYKTDQPIQGPPIIILTNDGMSEFQPRIKLY
>Acyrthosiphon_pisum_XP_003246947
IRPENGPISITELLHQYPCCPPDAYKNIKEYYLNKNLNYLCARDKYVDRDLELWNIKL-MHMSLKDFDNYYKDDNVNELLLYQFFFLTDVLNVIDKRVPKCNTLAIHAPPNAGKNYFFDAVAGVLNFAFMEAVKRLVLWNNHVNELKALLGGDSCRVAVKYKSDQALQGPPIIILTNDGMSAFQPRIKLY
>Acyrthosiphon_pisum_J9KNE3
----------MPKAQRYLGQAEGGLLAWSDGEVSWDFSCEPSSSSVLVQDSKNAGGENGGPKGRKKDSK-------HAWIRPEN--GPINVLNVIDKRVPKCNTLAIHAPPNAGKNYFFDAVAGVLNFAFMEAVKRLVLWNNHVNELKALLGGDSCRVAVKYKSDQALQGPPIIILTNDGMSAFQPRIKLY
>Helicoverpa_armigera_densovirus_HQ613271
NISTENLLEHITSIVCYPMDHVFQTRDWYTGKYKYLIS----SDKLVKRCYSILQKQL-MNWSYNDFLNHYRKDDTEELLDFQLREFVTDLWDVLEKKVPKKNTFQIISPPSAGKNFLLDAIFGMISFPLMEAVKRVNCWNSAEDTIKMILGGDPIKAAYKYESERNIARTPVIVMSNKINDAFEDRIITY
>Myzus_persicae_densovirus_AY148187
GSQEGPRQKKSRRKQRFRMIREIDVSKPQSIEELIYRYCCPPEAFYNIPEFYANTNINWIDMKDFKVTIPLRNWAAKELLNYQFIEFLTTLYNVIDKRVPKLNSICIKSPPSAGKNFFFDAVAGMFNFSWADGAKRLVLWNYHIEKIKELLGGDTTRIHVKYANDVSVQRVPIIILTNNSHPAFNDRLRSY
>Dendrolimus_punctatus_densovirus_AY665654
GDSVQKIIRFIQQKPTWPLEHVFQTKEWLDGPFGTWVS----SDKTVKRAIEIFSLQT-MHYTYTQFVDYYRSLECERLLNYQCKVFINDLWDILEKKRPKQNTFQIIGEPSAGKNYFIDAILGIIQFPLMEAVRRVNVWNAALDTIKMLLAGDPLKVNVKYQKEQYLQKTPIIVMTNKNDLAFHDRVCTY
>Bemisia_tabaci_EZ966062
----------------------------------------------------------------------------------------KKTFKKNTLFILSPPNSGKNYFFD-----------CNIQAVINSGHIGNLTKIHLFLWKQLESFY----GMNRIL-------SRMHLRI-----
>Sitobion_avenae_GAPL01021454
GVQSDGSNSGAVEKIEKRRLKRKYNKSVVDVDPNYPKNCCPPEAFYNIPEFYANRNVNWIDLKDFKITIPLRNWAAKNLLMVQFTEFLTTLYNVLDKKIPKLNSICIKSPPSAGKNFFFDAVAGMFNFSWADGAKRLVLWNYHLEKIKELLGGDTTRIHVKYHNDVSIQGVPIIILTNNNHPAFRDRLRTY
>Lygus_hesperus_A0A0A9YTR0
RKLTVNQIGELVNSMAAIPIRNCILSKRWRFNKDVKFI---TEEDKIFRRAVIYVESKCIYRSTRDFVDFYKFINPNDMLLFQFREFLQSCYDIIEKIIPKKNCLEIVSPPSSGKNFFIDFMSGNINFPFQDCARRLLIWNSALDTCKMLFGGDPLPAKIKFCGDATIPRTPVFVLSNKNDTAFNDRMYKF
>Ixodes_scapularis_XP_002401871
------------------------------------------------------------MDVSASYDAM------VRLLEYQLQEFVHDLYNLLERRVPKKNCMEVVSPPSAGKNFFFDAVVGNINFPLQDAVKRVLIWNAAYETIKKIFGGDVDNVAVKYSPDMIVTRTPVIVLSNNRDEAFNHRMFRY
>Rhipicephalus_microplus_EW679313
---------------------------------------------------------------------------------------MSRRRYRKKNCMEIVSPRVQAKTFFSNLFFGTISFPLQDTVRRILVWSSAF-----------------------------------------------
>Rhipicephalus_appendiculatus_CD786958
TQPLDFIEMYKNTQPLFDGPHSDLATFYMDVPTSLDAM-------------------------------------MELLNFQFSAFVNNLYSLVKKAAPKKNCMDIVSPPSAGKKFFLNPIFGTISFPLQDTVRRILLWNSVFDTVKKKFGGDVDSVAVKYSADQTISRTAVIVLSNYDDEAFNHRMWRY
>Rhipicephalus_pulchellus_L7M443
---------------------------------------------MDVPASFEAM---------------------MELLNFQFSAFVNNLYSLVEKAVPKKNCMEIVSPPSAGKNFFFDPILGTISFPLQDTVRRILVWNSAFDTVKKIFGGDVDSVAVKYSADQTISRTPVIVLSNNDDDAFNHRMWRY
>Bemisia_tabaci_EZ941358
---------------------------------------ASDTPSEYYFGIIKSFDVCMELLSWQMGFENI-------------KHFLQDIFDVLEKVRPKVNTLLILGAPNSGKNFFFDSVIGQMNFPLQECVRRVILWN-------------------------------------ESRSFEN-----
>Bicyclus_anynana_GE699248
---------------------------------------YVADWNNVGNYYYDIQTSVSVL---------------EELVKYQNYIFLRELYWLVDKKIPKKNCMSIVGPPNSGKNYFFDCVSGQIRFPLQDAVRRILLWNGSEEVLKLLFGGDTCPARIKYEGDASIRRTPIIVQSNKNHPAFESRVWKY
>Chilo_suppressalis_GAJS01033341
---------------------------------------------------------------------------------------------------------------------------------------------------------------------MLFGGDTCPARIKYEGDASIRRTPIIVQTRAQTSAFTSRVWRY
>Cherax_quadricarinatus_densovirus_KP410261
ERIQLKTVELLELYPICPPEAIVKHRIWRCDPELRFKNISSFKDTLTTYSMNEYQKMYNNPDCIPIFSAGYGNYETDELVNYQCIDFVTTLYNVLERKVPKLNCIVIHSPPSAGKNFFFDAVKGHLNFPFQDAERRIVLWNEFLEQIKEILGGDSTSVNVKYQSDTPVYRTPVIVLTNNNHSAFIDRIRVF
>Sea_star_densovirus_KM052275
KRKLGAQERIQIRVVEMLQLNPICPPEAIIKHRVWRNDPDLRFKDMTHKEIKTAVNSFSDNLTTFSMSDYQSMYNNDKLVQYQCLDFVNTLYNVLERKIPKLNCICIYSPPSAGKNFFFDAIKGHLAFPFQDAERRIVLWNEFLEPIKELLGGDSTSVNVKYMHDTPVYRTPMIVLTNNTHPAFKDRVRVF
>Ampelisca_abdita_JZ096885
---------------------------------------PIFSAGFLAFENYYYNIENSVKAM-----------TEIIMYQCLNFVT?LYNVCERKKPKLNSICIYSPPSSGKNFFFDAITGELVFAFQNMERRIVLWNEYLDLIKKLLGGDTCTVQVKYAHDCVVYRTPVIITTNNNHHAFEDRLRTF
>Ampelisca_abdita_JZ088717
---------------------------------------------------------------------------MYQCLNFVTTLYNVCERKKPKLNSICIYSPPSSGKNFFFDAITGELVFAFQNMERRIVLWNEYLDLIKKLLGGDTCTVQVKYAHDCVVYRTPVIITTNNNHHAFEDRLRTF
>Ampelisca_abdita_JZ093383
---------------------------------------------------------------------------TEIIMYQCLNFVTTLYNVCERKKPKLNSICIYSPPSSGKNFFFDAITGELVFAFQNMERRIVLWNEYLDLIKKLLGGDTCTVQVKYAHDCVVYRTPVIITTNNNHHAFEDRLRTF
>Ampelisca_abdita_JZ096796
------AVGLLRKHPICPPEAICKTSIWREDPDLRFKNYCDREIKAAVASFRDELINYTIDDYQKMYNKETC----LPIFSAGFLAFENYYYNIEN-SVKAMTEIIMY-----------------QCGEEATL-------NFVTTLYNVCER------------KKPKLN-------SICIYSP-----
>Ampelisca_abdita_JZ093297
ERIQLRAVGLLRKHPICPPEAICKTSIWREDPDLRFKNVASFRDELINYTIDDYQKMYNKETCLPIFSAGFLAFENTEIIMYQCLNFVTTLYNVCERKKPKLNSICIYSPPSSGKNFFFDAITGELGTA-----RRNVF-----------------------------------------------
>Periplaneta_fuliginosa_densovirus_AF192260
SDIREEMEKIVNENPVCPLAGIISTRQWLTHKDLRYLR----ADNETVKSFLDAKSEEMCYWTLHDFNTFYSQPYCNKLLAYQFKSFLNTFYNVLERKLPKCNTICVWSPPSAGKNFFFDVYLGQLNFSLQEATKRVLLWNAYTDTLKMLTGGDALCVRVKQKKDCHVYKTPLIVLTNNHELAFVDRVKVY
>Bombus_impatiens_JI097654
----------------------------------------------------------------------------------------------------M---------GNFSFPMMDCIKRVIMWNSALETLKMVFGGDTCPVKVKYLGDKLLLCTPIICLSNNQDDAFTCRMFTY
>Teleopsis_dalmanni_GBBP01040481
-----------------------------------------------------------------------------------------------------------------MECVKRIILWNSATETLKMLFGGDTVTAKVKYCNDAVIMRTPIIILSNNNDIAFRSRMFTY
>Kerria_lacca_GBDP01003119
---------------------------------------------------------------------------------------MYAKKEAKRNTLFVLSPPNAGKNYFFDAIVGQLNFPMQECVKRIILWNEHFEEIKTLLGGDNANAKIKHQCDSIIPRTPIIMLGNQKDEAFNTRILKY
>Kerria_lacca_GBDO01030308
---------------------------------------------MQAVIRRISIEITEWQIENFMTKYNNCDPYLIFNAPKYYAIDKTYIMYAKKEAKRNTLFVLSPPNAGKNYFFDAIVGQLNFPMQECVKRIILWNEHFEEIKTLLGGDNANAKIKHQCDSIIPRTPIIMLGNQKDEAFNTRILKY
>Corydalinae_sp._GADH01010824
-----------LKYYCCPVSAVRDVKEFREDDLL----CDPKNKDYVQAAFDDFGKDL-IDLSLRDFYNMLKDRKPDQLIKFQFCIFLTSLVDVLDKRLPKCNSLCIKSPPSAGKNFFFDMIFGQLVFAFQEAPKRILLWNSLTDTIKMMLGGDPYTVRVKHAMDTHVKRTPVIILTNETNIAFRERIVKF
>Kerria_lacca_GBDP01040339
RSKGQKLYEWFQQFPTGPIKQIINTRHWSQGPYK----FMPKNKPILITCFENMAFEF-LDMNVKDYFKYFNNKDPDELLKFQYTGFLTNVLNIVDKKILKKNALFIHSKPNAGKNYFFDAIIGQLSFPMQECVKRIILRN----------------NI-------------------------------
>Kerria_lacca_GBDP01000144
RSKGQKLYEWFQQFPTGPIKQIINTRHWSQGPYK----FMPKNKPILITCFENMAFEF-LDMNVKDYFKYFNNKDPDELLKFQYTGFLTNVLNIVDKKILKKNALFIHSKPNAGKNYFFDAIIGQLSFPMQECVKRIILRN----------------NI-------------------------------
>Culex_pipiens_densovirus_FJ810126
AAIKQETKALLLKYYPCPLSAIRDVSEFRNNNLL----SDPKNREYIDAAFVDFGKDVNQMTIRQIYEILKGHTDPEDFLKFQFTQFLVDLIDVLD-RVPKQNAFAVISPPSSGKNFFFDAICGQLHNLFQEAPKRLLLWNAMTDTIKMMMGGDPYTVRVKYQGDTHVTRTPVLILTNNQESAFKDRIKVY
>Culex_pipiens_x
---------------------------------------------------------------------------------------------------------AMTDTIKMMMGGDPYTVRVKYQGDTHVTRTPVLI----------------
>Helicoverpa_armigera_densovirus__JQ894784
AGKFAYIRQKTKALLRKYYCSPIIAIRDVDEFRDDDLLCDPKNRDYVEAACEDFGKDL-NDMSLRQIYNLLTEDYNTELLTFQCVEFLTNLVNVLDRKKPKLNTFVVYSPPTAGKNFFFDMIFGQLLFAFQEAPKRVLLWNSLTDTVKMMLGGDPYTVRVKNRGDQHVKRTPVVILTNNYEIAFNERIVQY
>Diatraea_saccharalis_densovirus_AF036333
AGKFAYIRKKTKALLRKYYVSPISAICDVPEFRDDDLLCDPKNRDYIQAACEDFGKDL-NAMSLREIYNLLTEDYNNELLKYQCVEFLTNLVNVLDRRIPKLNAFLIISPPSGGKNFFFDMIFGQLLFAFQEAPKRVLLWNSLTDTIKMMFGGDPYTVRVKNRMDAHVKRTPVIILTNNYELAFSDRIIQY
>Junonia_coenia_densovirus__KC883978
AGKFAYIRKKTKALLRKYYVSPVSAICDVPEFRDDDLLCDPKNRDYIQAACDDFGKDL-NAMSLREIYNLLTEDYNIELLKYQCVEFLTNLVNVLDRRIPKLNAFLIISPPSAGKNFFFDMIFGQLLFAFQEAPKRVLLWNSLTDTIKMMFGGDPYTVRVKNRMDAHVKRTPVIILTNNYETAFSDRIIQY
>Pseudoplusia_includens_densovirus_JX645046
AGKFAYIRKKTKALLRKYYVSPVSAICDVPEFRDDDLLCDPKNRDYIQAACDDFGKDLNAMSLREIYNLLTEDYNFVELLKYQCVEFLTNLVNVLDRRIPKLNAFLILSPPSAGKNFFFDMIFGQLLFAFQEAPKRVLLWNSLTDTIKMMFGGDPYTVRVKNRMDAHVKRTPVIILTNNYETAFSDRIIQY
>Galleria_mellonella_densovirus_L32896
AGKFAYIRQKTKALLRKYYVSPISAICDVPEFRDDDLLCDPKNRDYIQAACDDFGKDL-NAMSLREIYDLLTEDYNDELLKYQCVEFLTNLVNVLDRRIPKLNAFLVMSPPSAGKNFFFDMIFGQLLFAFQEAPKRVLLWNSLTDTIKMMFGGDPYTVRVKNRMDAHVKRTPVIILTNNYETAFADRIIQY
>Mythimna_loreyi_densovirus_AY461507
AGKFAYIRQKTKALLRKYYVSPISAICDVPEFRDDDLLCDPKNRDYIQAACDDFGKDLNAMSLREIYDLLTEDCNFDELLKYQCVEFLTNLVNVLDRRVPKLNAFLVMSPPSAGKNFFFDMIFGQLLFAFQEAPKRVLLWNSLTDTIKMMFGGDPYTVRVKNRMDAHVKRTPVIILTNNYETAFADRIIQY
>Agrotis_segetum_GBCW01035751
---------------------------------------------------------------------------------MIMESLVNIHY-NIQRLTGL---TPQS-------------SFNYASLLTNACFMDNQFEQWKLLAARQPMPTDVKYKNRQKVTNCILYTASNYTADAEEARTIT-
>Chilo_suppressalis_GAJS01023148
----------MATFSKRNKGIKIMCTKQYNEKIRTYIDIARVLVFQESIKQRFERAKKKNKIRFEDREDVSNEHEDKEIISNNNECFFRNTYEHFMKKTGKRNNLFFLGPPSTGKTMIMNSLVGLVSFNFSGLLRNACLMDNQFEQWKMLASGMPMSADEKG--PM-------------------------
>Acromyrmex_echinatior_EGI68937
RKNVVEAITNLIEENEIWTYVEWELKINYDTKEELLKEFGLQTDTSSVPSDWWLPIQTSQNRRTFDKLCETAWGIDTFDFETSQQKFDATRFDALTRERKHRDSDILTGGRD--RRLSPNEVTKDVTVPWPKRKRRHRKQQSPLEALQRHIQRQALSVPPTSQDPASLGSIDIVIAAPTSDSKPSDRIDGL
>Acromyrmex_echinatior_F4WA07
RKNVVEAITNLIEENEIWTYVEWELKINYDTKEELLKEFGLQTDTSSVPSDWWLPIQTSQNRRTFDKLCETAWGIDTFDFETSQQKFDATRFDALTRERKHRDSDILTGGRD--RRLSPNEVTKDVTVPWPKRKRRHRKQQSPLEALQRHIQRQALSVPPTSQDPASLGSIDIVIAAPTSDSKPSDRIDGL
>Acromyrmex_echinatior_EGI69346
TTEVIEAITQSRQQLESRMEQHVQIGWKKNRVYIILGHLPPESESEEARVSRAAPAKPSGSKAWATIIARRKRKQKTELLEGDEEPTIGHASSIKKRTEKDESTEKEDNPPLE-RTSSSETTTSGTKIGLKDFDKAINELANYEECIQYIEQRREERASGTEKRKNVVEAITSLIEENDKEELLKE-----
>Acromyrmex_echinatior_F4W8Y7
TTEVIEAITQSRQQLESRMEQHVQIGWKKNRVYIILGHLPPESESEEARVSRAAPAKPSGSKAWATIIARRKRKQKTELLEGDEEPTIGHASSIKKRTEKDESTEKEDNPPLE-RTSSSETTTSGTKIGLKDFDKAINELANYEECIQYIEQRREERASGTEKRKNVVEAITSLIEENDKEELLKE-----
```

```
>Acromyrmex_echinatior_XP_011068621
GSPGTNITTGERKRGSESIARSTSETEWQQSVGDDHRKKKKKTKTNGDGTIGRRRGNSPSSNRTYHWTCILHKKNQKRRIDRKGIVFGATKDNNLVQRGHIAKHMCATGHTE--INITCQHLRGTFKIGLKDFDKAINELANYEECIQYIEQRREERASGTEKRKNVVEAITSLIEENDELKINYDELLKE
>Atta_cephalotes_W4VWX9
RKNVIEVITQLIKKIQNDNLRRLGIKNRFPNERTIIERTQNNTKQKRNNNTSIYKYVSYEEITYRDLTNKYAKILRYEIFEKNRIEFYAWLILIKNMDLVKINGMVLEGPTNAGKSMIIDCTKPVYPITLDNKDQRIKIIT-HEEEYTQIAGA---------ID--RIKRAPTYVTPYDKRDKIEKKLREK
>Atta_cephalotes_W4X5W1
KNRVYITIGRLAEGREERAQPLMETNEHARVARATTTIVAKPGGSKAWRIVQARRKRKREEHQDRVGDTNIRSRRPAHHIRSNAGRIAKHMRAITA-ETAEIAISCQRIKYL--RNFILY---GVETFHYTGAKPELKIIDKKKKERRKNGTTDTHETCVQYIEHRREERATAARLGSDKRKNVVE-----
>Atta_cephalotes_XP_012064512
RKNVVEAITHLIKKYKITTYADXELKIDFQTKEQLLKEFGLQTDTYAHKIIRNKRNNNTSMYKYVSYEEITYRDLTYEIFEKNKVEFYAWLISIKNMDLVKINGMVLEGPMNAGKSMIIDITAGAITYVYDFTTIYLIERDKYQENPERLKTSEKTTIDLEIEMGHATKRAKFTDDGEDLDAANRTTLKRQ
>Hymenolepis_microstoma_CDS26524
-------------------------------------------------------------------------------------------------MEQRHMYLNLL------EELRSR---EVRSFDFQKFT-------EEITAFNAYMGIQWCDVDMIR----ALNCER-------EAKERAN-----
>Hymenolepis_microstoma_CDS33099
GNVVIETELEVTLHSVPDNIAPEDKEVYINDNSIDLLHYMGAEGKQFKWEEPEFKQEEPEIEKVKPEEPEIKKIKKDSIFVDENYGFLGGGLAGTV-VGGIVNALTTGFTTT--SNAVQQTEI---PFPANIADDEIVSIEPLINRADADENGRSEAITSARSSPSFLGHDPEIFPSTQQLGDMET-----
>Thaumetopoea_pityocampa_GBZB01000242
QPNQHQELIFGKSYHFTLTNGLADFRHVLSSPSGYYLAHGIPWERLLMYISEGELLRLYRDYTALKVEEVICEIYSQTAYNGNFTEWSENFPNISASTSRDISNPVIVEYPI--PYGFSNDVGGTTAYGWEKRFNGIIYAENYMTPIPGLENG-YFMTTITERNDAQIRPIPKALSATKQKCAPQAFMIGF
>Thaumetopoea_pityocampa_GBYZ01001450
QPNQHQELIFGKSYHFTLTNGLADFRHVLSSPSGYYLAHGIPWERLLMYISEGELLRLYRDYTALKVEEVICEIYSQTAYNGNFTEWSENFPNISASTSRDISNPVIVEYPI--PYGFSNDVGGTTAYGWEKRFNGIIYAENYMTPIPGLENG-YFMTTITERNDAQIRPIPKALSATKQKCAPQAFMIGF
>Porcine_bocavirus_5_HQ223038
MSKRESLVLDCMNRAIESDCLTYEQLVDKHPELIIMMESQAGGNRLIEQTLNMVHIKLTQKYTAMTYLVKRFPTFEIRLLNFQGWQVGHWLCCVLDKKSGKQNTVSFYGPASTGKTNLAKAIVGNVNFVSNDCSKLIVWWEDWVEPAKCVLGGTTVRVDRKHKDSQLLPQTPCIISTNNHCKPLKDRVVQL
>Human_bocavirus_1_JQ923422
PNKKEKLMLNLLDKCKELNLLVYEDLVANCPELLLMLEGQPGGARLIEQVLGMHHINVCSNFTALTYLFHLHPVTSLQLLLIQGLAVGHALCCVLNKQFGKQNTVCFYGPASTGKTNMAKAIVGCVGFVFNDCRRLVVWWEDWVEPAKCILGGTECRIDVKHRDSVLLTQTPVIISTNHHAAPLKERVIQL
>Human_bocavirus_4_FJ973561
ANKKEKLMLNLLDKCDELNLLVYEDLVSACPDLLLMLEGQPGGARLIEQVLGMHHIKVCAKHTALSFLFHLHPDQLLKLLLIQGLQVGHAICCVLNKQMGKQNTICFYGPASTGKTNFAKAIVGCVGFVFNDCRRLIIWWEDWVEPAKCILGGTECRIDVKHKDSVLLQQTPVIISTNHHAAPLKERVLQL
>Porcine_bocavirus_1_HM053693
MTKRGGLMLDCMRRCFTENITSYEEMVRECPDLTIMIESQAGGSRLIEQVMGMTHIKIVQSYTALGYVQKLFGEKAFRLLNFQGWQAGHWVCCVLDKKSGKQNTLCFYGPASTGKTNLAKSIVGCVNFVFNDCAKLIVWWEDWVEQAKCILGGTEFRIDRKNRDSMLLPKTPVVISSNNHSRPLRERIVQW
>Porcine_bocavirus_3_JF429834
LTRKQHVMLDTLQRCQEEFICTKEELTMLHPDLVIMFESTPSGSRTLEEVLEMHRVRITRTETALSYIRRQFPDSKVRLLNIQGIQVGHWVATVLSKKAGKQNTICFFGPASTGKTNLAKAIAGCVSFVFNDCQKLICWWEDWVEPAKCLMGGTSFRVDRKHKDSAEQPHTPLILSTNHHEKPIRDRVVQF
>Lamellibrachia_sp._x5
----------------------------------------CQIPFTQITKFPGSWANYLSQKPRITWRVPICQMME--------WVFADYIERELV-PERKVDDAVA-GP----SNVSAN------DFQSGKSMYKYLKWLFGHDNRESLIKG-------------ALAAA--------DTSTFEK-----
>Messor_concolor_x
-------------------------------------------------------------------------------------------------RITNFLHTTPAGSAE--ITVTCQLLQGLKKFHYGTKIQELKEIDNLTQELLKECDE---------NDEIDLG----------ECKKYIE-----
>Hymenolepis_microstoma_CDS27819
RADDEPVPEEEVLRRETRDAKTVGGWEN-------------------KTTFMSNPTMPFGKLLTLDYTGH------SVVFRKRYMVTLPGLHKEIP-STENATAFVLN------TTFLESFVC---RYRFKSNKTVSTKWNARIKIIRLIPSTKQATGGVVSVDKVQFDNASYMLVGISNQIQPLQKMSTY
>Eidolon_helvum_parvovirus_1_JQ037753
EGKSAERFLNLIDWLVDQGIATEKKWLEADRDGYRSFLSSSGGVLQAKNALNIARREMVLSQPLVRYLAKGGAWDECDIFRLNGPSLVARYMACWAGHWPKRRALWLWGPASTGKTVIAAAIAGCVNFPFNDCHQPLVWWENIVEVAKAILGGSPVRLDVKNKGSEDFLPTAVIITSNGHQDALRTRMCMI
>Human_parvovirus_4_AY622943
AGKGTQRFMDLIDWLVENGIATEKRWLSVNKLSYRSFLGSSGGVLQATNALQIAKREMVLAHPLLSYLTKNASAFEAQLFSLNGWYFAAWARGVW----PKRRAIWLWGPASTGKTLLAAAIAGCVNFPFNDCHQSLVWWENIVEVAKAVLGGAPVRLDVKNKGSEDYIPTCVIITSNGGLAPLPERSFFK
>Bovine_hokovirus_EU200669
GGKGTDRFLSLIDWLVEQGIATERRWLSADKRSYRSFLGSSGGVLQARNALLVARREMVLAHPLLGYLQRGGSAVAIELFKLNAWYFAAWAQGIW----PKRRALWLWGPASTGKTLLAAAIAGCVNFPFNDCHQSLVWWENIVEVAKAILGGAPVRLDVKNKGSEDFIPTCVIITSNGHQEALQTRITMF
>Porcine_hokovirus_EU200677
AGKGTERFLNLIDWLVEEGIATERRWLATDKKSYRSFLGSSGGVLQARNALQVARREMVLARPLLGYLRRGGSDIATELFRINGWYFAAWAAGAWA----KRRAMWLWGPASTGKTLLAAAIAGCVNFPFNDCHQSLVWWENIVEVAKAILGGAPVRLDVKNKGSEDFIPTCVIITSNGHQEALQTRITMF
>Porcine_parvovirus_4_GQ387499
KCATGEKMLDIVQWLVDNNICSESRWENKNALSLYSFLATQAGGYMAKQCLRIAQQKLLKEKPLGLTLMDFKDMNAHYIFAINNPKIASVIMYFWSKQTGKRNCVWFYGPATTGKTNMAQAICGNVNFPFQDIAAQVGWWEDMVEAAKALLGGTALRIDRKCMQSIEVNSPPFLITSNVHQQPLEDRMIKF
>Oryctolagus_cuniculus_XP_008259747
RTKISQKYMAVVNWHVENGTTSGKPWMQENSKSYLSFSSTKNARSQIRSALDNFTKVMGLTIMVLDCLVGVLALPDHQILQMSGRACAGSVWAAWCCQYGRRNTTWLFGPVTTRRSSIAEATAGCAIFPFRGCVQMTIWWETVVDAVKATPRGGKVRADQICKSSAQVEPTPAVITGNAHQQRLQDQRSTF
>Oryctolagus_cuniculus_GBCK01348906
RTKISQKYMAVVNWHVENGTTSGKPWMQENSKSYLSFSSTKNARSQIRSALDNFTKVMGLTIMVLDCLVGVLALPDHQILQMSGRACAGSVWAAWCCQYGRRNTTWLFGPVTTRRSSIAEATAGCAIFPFRGCVQMTIWWETVVDAVKATPRGGKVRADQICKSSAQVEPTPAVITGNAHQQRLQDQRSTF
>Goose_parvovirus__GPU25749
SNRAAKNYSNLVDWLIEMGITSEKQWLTENRESYRSFQATSSNNRQVKAALENARAEMLLTKTATDYLIGKDPVLDYQILKMNNPQYIGSILCGWVREFNKRNAIWLYGPATTGKTNIAEAIAGCVNFPFNDCVKMLIWWEKVVESAKAILGGSAVRVDQKCKGSVCIEPTPVIITSNTHRIPLEEQIVLS
>California_sea_lion_adeno-associated_virus_1_JN420372
RNRTSQKYMALVSWLVENGITSEKQWIQEDQESYLSFNAAGSSRSQIKSALDNASRIMSLTKKASDYLVGQSVPEDYQLFKMNGPAYLGSILLGWCGRFGKRNTVWLYGPATTGKTNLAEAIAGCVNFPFNDCVKMLIWWEKVVESAKAILGGSKVRVDQKCKSSVQIDSTPVIITSNTHRQPLEDRINLE
>Adeno-associated_virus_5_AF085716
KSKTSQKYMALVNWLVEHGITSEKQWIQENQESYLSFNSTGNSRSQIKAALDNATKIMSLTKSAVDYLVGSSVPEDWQIFEMNGPAYAGSILYGWCRSFNKRNTVWLYGPATTGKTNIAEAIAGCVNFPFNDCVKMLIWWEKVVESAKAILGGSKVRVDQKCKSSVQIDSTPVIVTSNTHQQPLEDRMFKF
>Adeno-associated_virus_1_AF063497
RSKTSARYMELVGWLVDRGITSEKQWIQEDQASYISFNAASNSRSQIKAALDNAGKIMALTKSAPDYLVGPAPPADYRILELNGPAYAGSVFLGWAKRFGKRNTIWLFGPATTGKTNIAEAIAGCVNFPFNDCVKMVIWWEKVVESAKAILGGSKVRVDQKCKSSAQIDPTPVIVTSNTHQQPLQDKFELT
>Octodon_degus_XP_004634735
RTKRSQRYMDLVKWLVEKGITSEKQWMEEDEESFLSFNATGNTRSQIRAALDNTRQKLIWTKTAKDYLVGSSAPPEYQIFDMNGPLYAGSILVGWCHQFGKRNTIWLFGPATTGKTNMAEAIAGCVNFPFNDCLKMIIWWEKVVESAKAILGGSKVRVDQKGKSSAEVAQTPVIMTSNTPEEHLQPRMFKF
>Bat_adeno-associated_virus_GU226971
STKSASEYMALVRWLVDNGIATEREWMREDSDGYLSYNATGATRAQIKAALDNAARIMVNTKTAADYLVGRNPPLDYRLFRMNGPAYAGSVLLGWCTGFGKRNTVWLFGPATTGKTNLAEAISGCVNFPFNDCVKMIIWWEKVVESAKAILGGSKVRVDQKCKNSQQIEPTPVIITSNTHRQPLEDRMFKF
>Chinchilla_lanigera_GBDF01150884
----------LVQWLVRIGMTSEQAWAREDCDSYVSFHASKDSRGQVKAALRNARTLCLTKTAADYLVAERAPDDIYRLFSLNSPAYAGSLLLGWCREFGKRNTVWLFGPPSTGKTNLAEAIAGCVSFPFRDCVKMLIWWERMLESAKAVLGGSAVRVPQKREVSSQIEPTPVIITSSQHRKALEERMFKL
>Eunicella_cavolinii_x
GRSPRCILKLCLDHGMADTDELLQNLEDLEPNLNIDKFFHPTRARMEKQIDQQLKFKYVNMSWMQVMETVWGKRQGYKWIRANNEQFICDVFALMYKLHDKKNAMLLIGPSNSCKSKILHSITSMIEFPFERCQVAVIQHEEVVDLFKRVAEGAVVDCNKK-GTSFVLKRVPNIWCSNHDSVAMLNRVF--
>Latrodectus_hesperus_GBCS01014800
-------------------------------------------MNLVANSIPSAARTVETALNLVGRPDLAATAHS--------SSAIQGVQLIQE-SITSIPAFLTE------ATNLLS---GST---TTSEPDAAAARTGQGPSIQNLQSSTNPQLPFDS----AVNTDP-------SIPADQE-----
>Ascaris_suum_JI174103
SGRYCRAIWEAIQEILPRSITDISNHLYRSANIEMVID--ERFNAVASKLFDAFRENYLNKSWKDIMNGIPENYFDEKLVNFNGKVIIEEIYNVMDTIHMKKNCILFKGPPNSGKTLLANSIVQQMPFEFQSMMQRVILINITIETIKNICEGQAVSIDIKYMSNQVLPRTPLIITTNATNRAVNELTRSY
>Bovine_parvovirus_2_AF406966
RQASGDRMRDLVEWCIKNHVFTEDQYMAKFEESYYSFACTNQGRHMLQTSLELAAKRTTSMIPLGVYLAGFSNIQEFDLLQFQGPVVAGYIIYAWSRATGRRGALWFYGPGQTGKSIMAR---GCVNFPFQDLACQIGWWEDIVESAKALLSGGKIRVDRKCRDSVEITPPPFVITSNNHKKPLEDRMIKF
>Echinaster_spinulosus_GAVE01029446
SQSSNALMRSVMRHEDDRTKEIVKSMF-----------YKQNWNTLFNKVLGLVKIHNCDMNMAQMLDMAEREPSYKKWCAHQGKELYQSVQCILDKVLAKKNTLAFIGESNAGKSWVVETLCGEIGYAWQECPKRLIVFNAVIEKCKVVFEGSDCFVSVKQQGDAFLTRTPCIITSNNEETAIRNRCILY
>Luidia_clathrata_GAVQ01079362
----------------------------------------MTLDELSDRYRTASHWDEPNRYESLDRSCALVKRWC--------HTFVDDLYSIID-RLPKRNCLALVGQSNAGKTWLIN---------LAEVC-------CHVGEITQGTSS--------------------------------------
>Turkey_parvovirus__GU214706
TDWGNGGGETVIKTSIIEKLCMEALRLCRENHIFTMKAFKLKFPDKFMQFSSRNQGLVKLEETINLYCETIINEHNIRLCSYQGPKYVARLILCWLGQAGKKNALYFHGPANTGKTMMAESICGNVNFPFNDCHKAVLWWEEHVESAKCIMGGSSVRIDKKNQDSVLLCKTPIVITSNNHAAAIRARCLKF
>Schmidtea_mediterranea_GAKN01010353
------------------------------------------------------------------------------------RGGKINTIYLQG-NILYDNLFYSIGPSNTRKTFFVQLLTGCITFWTQDVDKDVVVAEENVDYFKLMLEGNMIRIEIKNKPAVNWKRIPVIVTSNQHQEALQNRMLVI
>Rhipicephalus_microplus_CK189748
-------------------------------------------------------------------------------------------------MHSEGRCLHSEGCPG--------------VFARGDTI------------SMQLFGGPPSRP--------GCTSCL-------QRPILPR-----
>Rhipicephalus_microplus_CK189747
-------------------------------------------------------------------------------------------------MHSEGRCLHSEGCPG--------------VFARGDTI------------SMQLFGGPPSRP--------GCTSCL-------QRPILPR-----
>Hymenolepis_microstoma_CDS26229
-------------MMVHDGYGQLDDTRVKHFPYFLYLASTNGTRFNCRKYHNWGHAEPELLCVAVGNPAICVNGQS--------TDGGAMAWNLAH-QRHGSNELLLC---------------ECAFFPFVCAIQRVSGYE---DVLDNIAGGGKVYAWRSS----GVLGAP--VKARK------------
>Hymenolepis_diminuta_LM393614
--MFELFQTLNIKSEEEFMQWIKFNPEQLPELWSRYPNWEKKLQQYVSVMIKTKSGLFDEPFHIKPYLVHVQHATCYSIFTNNDGLFLAEVDKIIRMTYPRINALVLRGPTSTGKTLIAKNIVETVAFYLQNLLHDVALMETTVQNFKELFAGSPLIVQVKNHAPRELKRIPCIVTTNQESEPIRRRIIE-
>Hymenolepis_diminuta_LM395168
-------------------------MKVIVDMPLLERKFALLQEENIYKDICKFVCILSDPDNIHDNDSLAGAKYLYSNFTNNGGLFIAEVDKIIRMSYPRINALVLRGPTSTGKTLIAKNIVGTVAFYLQNLLHDVALMETTVQNFKELFAGSPLIVQVKNHAPRELKRIPCIITTNQESEPIKKRIIE-
>Taenia_multiceps_JR938527
-------------------------------------------------------------------------------------------------MTPFINGFSVLVMPY--RHISLSLIQ---HFQFDNLLDAGALMETTKDDFKLLLGGIGLEVDVKYSGHKWLERVLVIQATNENRKCFTN-----
>Hymenolepis_nana_LM406075
RMHKYFNLLEELRIRQVRTFDEVFHKFNVDEITFMNSSIGIQWGEVARQQIRTLSNERLAKEKTNPYLQNLHELPHIAMLNTNSGALLGDIVQIMDKRKAKINTLCFRGQTNTGKTLLANLLT---SFHFFVFAKTLILVLALFNRL------ERCAD--------VVIRPPFISTTCS------------
>Hymenolepis_nana_LM409198
RMHKYFNLLEELRIRQVRTFDEVFHKFNVDEITLMNSSIGIQWGEVARQQIRALNNERLAKEKTNPYLQNLHELPHIAMLNANSGALLGDIVQIMDKRKTKINTLCFRGQTNTGKTLLANLVT---KYP-----SHFFVFATLILVLALFYRLERCAV--------VVIRPPFISTT--------------
>Hymenolepis_microstoma_CDS26228
----------------------------------------------MPPPSNVRLKLPSWLAAENAK------------------------------PDGELPVKEFH------QRYYLT------LIVYTNVPAISCLYVEHDDHMHVAFQA-SSNVKVER----LLNDCHVPLRTGSSAKVLSN-----
>Hymenolepis_diminuta_LM389526
VNNPVKMIQYFKFRNTPVIIGAELRDIFEIARSMPPLETAEQCYGIQLRTQTKAALNNKVDNRVSKYESLYEELKRYTIFTVHEMHFNANIGLQWR-EIAKQAIQAIN------EDLLLE---QTRTYLLQEK-RHDCYADRGVDWLEFMFNMNHIPVLLTD----IIKIMDMKVNKVNCLCFYGQ-----
>Hymenolepis_microstoma_CDS29136
YTPYRYYYTLVLRNYGKVPTSISCLYVEHEQHMHVLLQAKDNISRKVDRLLNDCLVPADNWWMIKNTRQLVRNVYLIQYFKYRGFEVGTELHEEWQADMPPIQPHVTCRRART-KSTIAE---RTEKFTLDELKSDIRQFEFSVNEIWINMDKKTTKVIAKQ----QIQSLNAERLQSEKQNSYLQ-----
>Hymenolepis_microstoma_CDS30830
------------------MYSVMHEADYLSSRIELIVS------------KWLPNSALHGSVSSVDYLRLSFAD--RACFRTHC-------------KWNLLTSCLLWGEGE--EEVGGE---GIVAFHFENLLRTVALIEETKNDYKCLLGGERLEIDIKCGARRFLQRVPVIVTTNESQLTSLA-----
>Hymenolepis_microstoma_CDJ12115
------------------MYSVMHEADYLSSRIELIVS------------KWLPNSALHGSVSSVDYLRLSFAD--RACFRTHC-------------KWNLLTSCLLWGEGE--EEVGGE---GIVAFHFENLLRTVALIEETKNDYKCLLGGERLEIDIKCGARRFLQRVPVIVTTNESQLTSLA-----
>Hymenolepis_microstoma_CDS30832
-------MNELTCYQPVRGDQENRVPNNEVPHKYYYTVVLRKYSKVPTSISCLYVEHDDHMHVLLHAKTNVSRKVNDCLIPPDDRQLVRNVYRIIQFKHQVLGIVCQRGEQK--------------AFHFENLLRTVALIEETKNDYKCLLGGERLEVDIKCGARRFLQRVPVIVTTNEDLG---------
>Hymenolepis_microstoma_CDJ12117
-------MNELTCYQPVRGDQENRVPNNEVPHKYYYTVVLRKYSKVPTSISCLYVEHDDHMHVLLHAKTNVSRKVNDCLIPPDDRQLVRNVYRIIQFKHQVLGIVCQRGEQK--------------AFHFENLLRTVALIEETKNDYKCLLGGERLEVDIKCGARRFLQRVPVIVTTNEDLG---------
>Hymenolepis_microstoma_CDS33272
------------------------------------------MAKQQIPSLNTERLQVEQASSYPNNLRNLKHDCTMMMLNQSGNELLTDIINIIYTKTTKMDTFCFKGQTNTGKTLLANLIAGPVAFHFDNLPRTVALMGITKNDYVSLEEV-----DLKS----------M------------------
>Hymenolepis_microstoma_CDJ14557
------------------------------------------MAKQQIPSLNTERLQVEQASSYPNNLRNLKHDCTMMMLNQSGNELLTDIINIIYTKTTKMDTFCFKGQTNTGKTLLANLIAGPVAFHFDNLPRTVALMGITKNDYVSLEEV-----DLKS----------M------------------
>Hymenolepis_microstoma_CDS35108
GVVSGSLMEYLSGSGEKLTISICGAAVQEITKFNSS--IGVQLREIAKQQMQSPNTQRLKDEQTKSHLENRRGMKHMMMLSQHGDELLKDIAYIMDKKTTKVNSLCFKGQTNTGKKLLANPHLGTVAFHFDNLLRTVTLMETTKSAYKCLLGGDRFEIDVNYGARRFLQRIPVVDTTNEDRAALYSRVKQY
>Hymenolepis_microstoma_CDI96179
GVVSGSLMEYLSGSGEKLTISICGAAVQEITKFNSS--IGVQLREIAKQQMQSPNTQRLKDEQTKSHLENRRGMKHMMMLSQHGDELLKDIAYIMDKKTTKVNSLCFKGQTNTGKKLLANPHLGTVAFHFDNLLRTVTLMETTKSAYKCLLGGDRFEIDVNYGARRFLQRIPVVDTTNEDRAALYSRVKQY
>Hymenolepis_microstoma_CDS34279
---------------------------------MQSPNTQRLKDEQTKSHLENRRGMKHEYSVGNTR---------MMMLSQHGDELLKDIAYIMDKKTTKVNSLCFKGQTNTGKKLLANFITGTVAFHFDNLLRTVTLMETTKSAYKCLLGGDRFEIDVNYGARRFLQRIPVVDTTNEDRAALYSRVKQY
>Hymenolepis_microstoma_CDJ15564
---------------------------------MQSPNTQRLKDEQTKSHLENRRGMKHEYSVGNTR---------MMMLSQHGDELLKDIAYIMDKKTTKVNSLCFKGQTNTGKKLLANFITGTVAFHFDNLLRTVTLMETTKSAYKCLLGGDRFEIDVNYGARRFLQRIPVVDTTNEDRAALYSRVKQY
>Hymenolepis_diminuta_LM391469
VKNVYLLLQYFKFRGEIHKVGQELSEEWLLAQELPAVEPRQPCFSDLRRTQTKSAATVRLESRHAKFTNLLEELKREDIFSKFSVKEITDLNTSMG-QWREIAKQQIQGMNA--ERLKME---KQCNYLLENLRHDCAVSNDLGANWLMMLLNNGINILLADKKTTKVNTLCFKGQTNTGKTLLAN-----
>Hymenolepis_microstoma_CDS30831
RAEIPGTTSGAGYERPSSPSFIGHDPFGIEEGQQLLPSTQRLGDVGSRTQNTLSSGMAEKDSSVDNILPTERIPQSTLMLNQNGYELLNDIIAIMEKKTTKANTLCFKSQTNTGKTLLANLITGTVAFHFDNLLRTAALMETTKNDYKCLFGGDRFEIDVKYGARRFLQRIPVVDTTNEDRAALYSRVKQ-
>Hymenolepis_microstoma_CDJ12116
RAEIPGTTSGAGYERPSSPSFIGHDPFGIEEGQQLLPSTQRLGDVGSRTQNTLSSGMAEKDSSVDNILPTERIPQSTLMLNQNGYELLNDIIAIMEKKTTKANTLCFKSQTNTGKTLLANLITGTVAFHFDNLLRTAALMETTKNDYKCLFGGDRFEIDVKYGARRFLQRIPVVDTTNEDRAALYSRVKQ-
>Hymenolepis_microstoma_CDS30612
----------------------------------------------------------------------------MMLLNQNGNELLTDIINIMDKRSTKMNTLCFKGQTNAGKTLCANLITGTVAFHFDNLLRTAALMEITLEEVDLKAMSDRAALGVKQNEPSSMGCPP-------APCPLAN-----
>Hymenolepis_microstoma_CDJ11897
----------------------------------------------------------------------------MMLLNQNGNELLTDIINIMDKRSTKMNTLCFKGQTNAGKTLCANLITGTVAFHFDNLLRTAALMEITLEEVDLKAMSDRAALGVKQNEPSSMGCPP-------APCPLAN-----
>Hymenolepis_microstoma_CDJ13509
```

```
-----------------------------------------------------------------------------------------------------MNTLCFKGQTNTGKTLLANPITATAAFHFDNLLRTVALMEITRNDYKCLLGGGRFKIDVNY-----------------GAREV-------
>Hymenolepis_microstoma_CDI96352
-----------------------------------------------------------------------------------------------------MNTLCFKVQTNTGKTLLANLITGTVAFHFDNLVRTVALMEITRNDYKCLLGGGRFESHVKY----GARRVCNVFRAALKQHTLNE-----
>Lepeophtheirus_salmonis_FK913417
----------------------------------------------------------------------------DIFFQENNAEFYTKLCIVANKEEPKVNAFILQGPANC----------------FSKLA-------------GLLLKGNACLV---------------------------------
>Lepeophtheirus_salmonis_JP304452
--------------------------------------------------------MVLLYWLLYIIL----------------YRILLHLCYFLG-RIYRAIPLLLEGADV--KASVKNSIL---KRTFITTNHSLCKWISAEDAAAIDERT-------------FVYNLSNKISSHRGRSSIPP-----
>Lepeophtheirus_salmonis_FK925681
--------------------------------------------------------MVLLYWLLYIIL----------------YRILLHLCYFLG-RIYRAIPLLLEGADV--KASVKNSIL---KRTFITTNHSLCKWISAEDAAAIDERT-------------FVYNLSNKISSHRGRSSIPP-----
>Lepeophtheirus_salmonis_JP302515
TKMSSALPALTILTTPCQTIPLLPNKPNIRLTPKLYIV--RLTNTVITSPTLKLSFKIIAEKGHNFYREPLNGHYPNVYAVLNGTHFAECLTNVTFQANGNTNWTQTYVPDLGTRETLEE---ASTAYSTLNELQEIKKWTRIYRAIPLLLEGADVKASVKNGPDSILKRTPFFITTNHDAAAIDERTFVY
>Lepeophtheirus_salmonis_FK918082
TKMSSALPALTILTTPCQTIPLLPNKPNIRLTPKLYIV--RLTNTVITSPTLKLSFKIIAEKGHNFYREPLNGHYPNVYAVLNGTHFAECLTNVTFQANGNTNWTQTYVPDLGTRETLEE---ASTAYSTLNELQEIKKWTRIYRAIPLLLEGADVKASVKNGPDSILKRTPFFITTNHSLCKWIS-----
>Caligus_rogercresseyi_GAZX01015685
NMRSATMDLIDKHSKYPYNLDDIVVRLENEEHEWLYETFGLSWKSHITDFIQNMNHRIRRETRLPLAEFHKAQIEDDHFFREQHKEFYTNLCMIANQEIPKVNAFVLQGPTNCFKSTLFRLLFTPMNFYLQSCLKDYIIWETDINEWKLLLEGAPVKASVK-SPDSILKRTPFFITTNHDAAALQERMYV-
>Caligus_rogercresseyi_GAZX01042735
------------------------------------------------------------------------------------PEFYTNLCIVANKELSKVNAFVLQGPTNCFKSSLFRLIFTPMTFYLQSCLKDYIIWEADINEWKLLLEGADVKASVKNGPDSMLKRTPFFITTNHDAAAINERTFVY
>Octopus_bimaculoides_KOF62817
----------------------------------------MKWNKFHIHQYHTVSTSLRIF---------------ENLLKIQTSNFVNNIWNILAQKIPKINTFALEGVPSSGKSYIARAMFSETSFPSQDMYTELCLFEKTLQTFK-LTEGADTAVAVKFKPSVTIKRTPIIITSNYEKEAFKQRIV--
>Euprymna_scolopes_DW283133
AVNVKHQAERYLERTPCLSTSNDDPWRHVPQERTAMHN--RMFLYEIKRPMPALKGWGVRELNPTMWLTVWRQYVMYQFQEEEDKRFTVQAVEGLS-PEKQAGYCKAR------RQLFHDLMAGEE---VEDWIAQPCELDHGCKAHRCPNAGRQSPSAPTFSQDTITEDTPVIILSDSETEARDD-----
>Euprymna_scolopes_DW277466
AVNVKHQAERYLERTPCLSTSNDDPWRHVPQERTAM--HNRMFLYEIKRPMPALKGWGVRELNPTMWLTVWRQYVMYQFQEEEDKRFTVQAVEGLS-PEKQAGYCKAR------RQLFHDLMAGEEVNNISDDSDVEDWIAELDETQPTQYCRHGCKACPNA----GNRQSP-------SAPTFSQ-----
>Euprymna_scolopes_DW277051
AVNVKHQAERYLERTPCLSTSNDDPWRHVPQERTAM--HNRMFLYEIKRPMPALKGWGVRELNPTMWLTVWRQYVMYQFQEEEDKRFTVQAVEGLS-PEKQAGYCKAR------RQLFHDLMAGEEVNNISDDSDVEDWIAELDETQPTQYCRHGCKACPNA----GNRQSP-------SAPTFSQ-----
>Lamellibrachia_sp._x3
-----------------MFQSVSRKAFDVEKALEIMQGFRARYEGINWGKFENDEKYLPVDTSHALF---------KHLLTFHDPAFVVDVWDILT-KRPKTNLLFIQGVPNSGKSFLVRSVCATVTFPFMELSASCGLIE--------------------------------------------------
>Lamellibrachia_sp._x1
-----------------LFDSVCTKAFTVDKSLEVMQGFRARYEQVRWEEFEGNDSYLSVDVSIRLL---------RHLLDHHEEAFVDDVWSILT-QRAKTNLLFVHGVPNSGKSFIVRSLAATVSFPFMELAASFGLIE--------------------------------------------------
>Lamellibrachia_sp._x10
------------------------------KSLETMQGFSARYDKIKWGEFEVNEAYLSVDVSIRLF---------RHLLAHHDPAFVEDVWNILTK-MPKTNLMFIHGVPNSGKSFIIRSIAATISFPFMELAASLGLIEEALQTFKKLCEG----T---------------------------------
>Armadillidium_nasatum_endogenous_virus_parvovirus_KT714028
AKTSHELLIRSSPTELSHVRNFLGKKYLVKIRNTMYLENIINFKREMREPIINRFIRVTPDNDNIQENWI------KNLFHVNGIDFLSWFVIIAD-RCFKKNTMVLLGPVNTGKTLLMR------------------------SLLQKENKG-------------NFVRCK-------GKLSFD------
>Sepia_officinalis_FO193416
------------------------------------------METNKKQKRILAANEGNMYWTIQDSCAIYEAR--CNFHNIDCEEFSKRLFDVCHHVLPKINSFHIQGPANAGKTYILS---GSMAFTFGGYVKALIFTDHNVDQAKCVLEGASTMVNVKNQNERLIRRTLCLSTSNEEEATLKARMYYY
>Hymenolepis_microstoma_CDS29534
-------------------------------------------------------------------------------------------------MNNSIVQATFETTTM--ESPLVK---SRLDWATWECARKVRYFLEHNDHMHVVFQAKE---NVSRKGGSLLNDCP--VPAEDSARQYSR-----
>Hymenolepis_microstoma_CDS28210
------------------------------------------------------MKKYQPPQPEIDMDDPIVK-----------VTFETTTMESQKKKFPSMPCKLVA------QNSLPDSMV---KVDLNDCPPAEDWFTQYARDIYLLLQYFKFHVTLKQVSEEMMEEME-------TARDLMP-----
>Armadillidium_vulgare_endogenous_virus_parvovirus_KM034115
---------------------------------------------------------------------------------------MAWFIIIADKRCHKINTLVVYGNTNTGKSLVIRTLLGYVKLMFDNLVKNFALLENVINELKLFFEGAKFEANIKNYPSKTLGRIPTFVTTNHDGNALKTRIKE-
>Armadillidium_vulgare_endogenous_virus_parvovirus_KM034113
--------------------------------------------------------------------------------------------------------MVVYGKTNTGKSLVIRTLLGYVKLMFDNLVKNYALLENIINELKLFLEGAKFEANIKNNPSKTLGRIPTFVTTNHDGNALKTR----
>Chinchilla_lanigera_GBDF01269873
PSQYYEVIVLIPSDFEEQLPDIPRDFVKWVDSVVWQLPQGSDVDLNLIEQKPLTVAHSVVQVLLRSWRRIFGSK----------PEYFVQFEKGW--QHFHLRALILTGGM---RPLVVG------RCLIRRMICQVVFFDNIPDWMAVTRTGHGAHKEKDFISAYLLPKTQ-------PELQW-------
>Euprymna_scolopes_GBHH01003248
-------------------------------------------------------------------------------------------------------MSVVLGPSD--------------NFTFGSCVKSLIFTEQNVEEAKCILEGTDTYVNVKHQSERILKRTPCLSTSND------------
>Aedes_albopictus_densovirus_3_AY310877
QQNLTDIILDKIKDKKITTAQQWENIIEPEFKIQLMKEFGLNVDSYVQRIVRIERTRIQQLIKSKTLTEIMVEILNEYMFKENNIHFLAWNEIIKTKRYKKINGMVLEGITNAGKSLILDNLLEEIGFHLDQLPAGSVLFEVNVGTWKLLLEGKTIKTDVKNKDKEPIERTPTWITT-AETSQILQRIKL-
>Aedes_albopictus_densovirus_2_X74945
QQNLTDIILDKIKEKKITTAQQWENIIEPEFKIQLMKEFGLNVDSYVQRIVRIERTRIQQIIKSKTLTEIMVEILNEYMFKENNIHFLAWNEIIKPKRYKKINGMVLEGITNAGKSLILDNLLEEIGFHLDQLPAGSVLFEVNVGTWKLLLEGKTIKTDVKNKDKEPIERTPTWITT-AETSQILQRIKL-
>Haemagogus_equinus_densovirus_AY605055
QQNLTDIILDKIKDKKITTAQQWENIIEPEFKIQLMKEFGLNVDSYVQRIVRIERTRIQQLIKSKTLTEIMVEILNEYMFKENNIHFLAWNEIIKTKRYKKINGMVLEGITNAGKSLILDNLLEEIGFHLDQLPAGSVLFEVNVGTWKLLLEGKTIKTDVKNKDKEPIERTPTWITT-AETSQILQRIKL-
>Aedes_albopictus_densovirus_1_AY095351
QQNLTDIILEKIKEKKITTAQQWENQIEPEFKIQLMKEFGLNVDSYVQRIVRIERTRIQQVIKAKTLTEIMLEILNEYLFKENNIHFLAWNEIIKTKRYKKINGMVLEGITNAGKSLILDNLLEEIGFHLDQVPAGSILFEVNVGTWKLLLEGKTIKTDVKNKDKEPIERTPTWITT-AETSQILQRIKL-
>Mosquito_densovirus_BR/07_GU452720
QQNLTDIILEKIKEKKITTAQQWENQIEPEFKIQLMKEFGLNVDSYVTRIVRIERTRIQQLIKAKTLTEIMLEILNEYLFKENNIHFLAWNEIIKTKRYKKINGMVLEGITNAGKSLILDNLLEEIGFHLDQVPAGSILFEVNVGTWKLLLEGKTIKTDVKNKDKEPIERTPTWITT-AETSQILQRIKL-
>Aedes_aegypti_densovirus_1_M37899
QQNLTDIILEKIKEKKITTAQQWENQIEPEFKIQLMKEFGLNVDSYVTRIVRIERTRIQQLIKAKTLTEIMLEILNEYLFKENNIHFLAWNEIIKTKRYKKINGMVLEGITNAGKSLILDNLLEEIGFHLDQVPAGSILFEVNVGTWKLLLEGKTIKTDVKNKDKEPIERTPTWITT-AETSQILQRIKL-
>Aedes_aegypti_densovirus_2_FJ360744
QQNLTDIILEKIKEKKITTAQQWENQIEPEFKIQLMKEFGLNVDSYVTRIVRIERTRIQQLIKAKTLTEIMLETLNEYLFKENNIHFLAWNEIIKTKRYKKINGMVLEGITNAGKSLILDNLLEEIGFHLDQVPAGSILFEVNVGTWKLLLEGKTIKTDVKNKDKEPIERTPTWITT-AETSQILQRIKL-
>Culex_pipiens_pallens_densovirus_EF579771
QQNLTDIILEKIKEKKITTAQQWENQIEPEFKIQLMKEFGLNVDSYVTRIVRIERTRIQQLIKAKTLTEIMLEILNEYLFKENNIHFLAWNEIIKTKRYKKINGMVLEGITNAGKSLILDNLLEEIGFHLDQVPAGSILFEVNVGTWKLLLEGKTIKTDVKNKDKEPIERTPTWITT-AETSQILQRIKL-
>Anopheles_gambiae__densovirus_EU233812
QQNLTEIILEKIKEKKITTAQQWENQIEPEFKIQLIKEFGLNVDSYVTRIVRIERTRIQQLIKAKTLTEIMLETLNEYLFKENNIHFLAWNEIIKTKRYKKINGMVLEGITNAGKSLILDNLLEEIGFHLDQVPAGSILFEVNVGTWKLLLEGKTIKTDVKNKDKEPIERTPTWITT-AETSQILQRIKL-
>Rhodnius_prolixus_T1I3J3
RKHISNPFKQHKQPQNKRRNNSIIEYIPTKLIYYMGQ-IGLQADSFIKRLLQNKQTLQKQLLKQTHYYDLLPETTEEKLFISNDNEFFAKFILIQSRQLNKINTFIIKGPTNTGKSLLINALLTLIGFHLDQLPAGYVLFETNIGTWKLLMEGSELTTDIKNQSKEIIERLPFL-----------------
>Extatosoma_tiaratum_GAWG01006018
----------------------------------------------------------------------------------------------------------MF------KEPLLD---------------------TNVGTWKLPLEGATITTDIKHSDKEMIHRLPIFVTTNCNLWNWCE-----
>Extatosoma_tiaratum_GAWG01117743
-----------------------------------------------------------------------------------------MFLRLLT------------DPLQ--PTLISR---ERDGFHYDQLPATCVLFENNVGTWKLLLEGAIITTDIKHSDKEKIPRIPVYITTNQEKEPIKQRIFI-
>Extatosoma_tiaratum_GAWG01005303
------------------------------------------ADKIIPQDVQWF----------------------HDFFRENDVDFYVKFIQIQSRMLKKINGFVLEGPTNTGKSMPARLLTEPLGFHLDQLPATAVLFE-------------------------LIDNTN-------------------
>Extatosoma_tiaratum_GAWG01139811
--------------------------------------CGTSMTSYIKILIKMYNTEKIRHIRQLHYYDIIQQHWNNTFFEKNDVNFYADFIRIQSCMLKKINGFVIEGPTNAGKSMLTRILT-------------------------------------------------TLLTRERDNTGFHF-----
>Medauroidea_extradentata_GAWD01093153
------------------------------------------QEENIHIPSLEWLDHFFHSNSIDI------------------PHFYADFIRIQS-MMKKINGFVIQGPTNAGKSMLTRLLTQDMGFHFDQLPSTCALYENNIGTWKLLLEGTPITTDIKHMDKEIIERIPFYLTTNAEASEIPPRTYIY
>Aretaon_asperrimus_GAWC01115021
-------------------------------------------------------------------------------------------------MVTRLLTEHLL------PALLTR------GFHFDQLPACCALYENNVGTWKLLLEGTLVTTDMKH-----------------------------
>Aretaon_asperrimus_GAWC01079978
RQNIVTTLDNTIREYKCRTYENFISTVPTDMQKTLLQLCGTSMTSYIKLLLKIYNTELMAKIKCSHYYDIIVEHYDETFFKLNKAIFYSQFIKIHSKMLKMIDAFVVEGPTNTGKSMVTRLLTALLGFHFDQLPACCAFYENNVGTWKLLLEGTLVTTDMKHTDKELIPRLPFYITTNNEHCEIPPRIFKF
>Latrodectus_hesperus_GBCS01020635
---------------------------------------------------------------------------------------MISTNGSLQ-ETPKKNTFVIQGPSNTGKTSFIRQLFAEISFLFQNCPADILVWEADADKCKLVFEGVQTQVNVKRCEPKTLFSTPVLITTNKDKDALMNRCYT-
>Crepidula_fornicata_x
--MVQGTSTIGKSYFYCTPFGSIPGVGHWCKEGAFGFMDCVDKLMIIADEFSIVPEMVEVSKSILRGNEVKVSVKNQQMVKKT-PMIACTNNSIVK-GLGATDCIAVR--SR--VHLLQLDLEGTKAWLARHANPKWLVWL--HDNIYMQQEGYNADFEMEY-ANEEFVSIP-------------------
>Echinococcus_granulosus_W6UKP4
--------------------------------------FNLRAVPTAGRRAGNPDLRTNQRRAHARTTETHASHPPEDMFLANEVDFAITLRIIMNCEDEKINTLVLYGPTNTGKSLICKTFLGSVAFAYENLLRKVALMEANQQDLKQILGGETFKGDMQTTQQA---PQPVQSTAQH------------
>Echinococcus_granulosus_CDS25081
LTGFTRTYYTFILRHYTRTPNAISEVLYTEHDDHMHVIYQSSTSNSPRKMERIIEECGVPPQAVPDIKMTKQLVRNIRYMRGRGDHYDHFLRVATSLEWPDCSVIPSE-----GRRILKS---AKEKFLAEEIMRKVRSMNTYQETFRLMADYQSYNMIVRK----ALETVRMMNVAHQRATDYAD-----
>Echinococcus_granulosus_EUB54832
--------------------------------------AQEKLLDLLKEELDNVRHGSPSHLCAYPINHSGP----EDMFSANEVDFAITLRIIMNCEDEKINTLVLYGPTNTGKSLICKTFLGCVAFAYENLLREVALMEANQQDLKQILGGEPFEVSVKYQNPDLLERLPVIVTTNEDAAAIEGRCKI-
>Echinococcus_multilocularis_U6HHY9
-------------------MNDMNKKFTYQETFRLMADYEQSYNMIVRKALETVRTMNVAHQRATDYMDLLKEELDEDMFSANEVDFAITLRIIMNCEDEKINTLVLYGPTNTGKSLICRSFLGSVAFAYENLLRKVALMEANQQDLKQILGGEPFEVHIKYQNPDLLERLPVIVTTNEDAAAIEGRCKI-
>Echinococcus_multilocularis_CDS36816
-------------------MNDMNKKFTYQETFRLMADYEQSYNMIVRKALETVRTMNVAHQRATDYMDLLKEELDEDMFSANEVDFAITLRIIMNCEDEKINTLVLYGPTNTGKSLICRSFLGSVAFAYENLLRKVALMEANQQDLKQILGGEPFEVHIKYQNPDLLERLPVIVTTNEDAAAIEGRCKI-
>Echinococcus_multilocularis_U6HDP5
KRQKFLDLAEEIIRRKVRSMNDMNKKFTYQETFRLMADYGQSYNMIVRKALETVRMMNVAHQRATDYMDLLKEELDEDMFSANEVDFAITLRIIMNCEDEKINTLVLYGPTNTGKSLICRSFLGSVAFAYENLLRKVALMEANQQDLKQILGGEPFEVHIKYQNPDLLERLPVIVTTNEDAAAIEGRCKI-
>Echinococcus_multilocularis_U6HC26
-------------------MNDMNKKFTYQETFRLMADYGQSYNMIVRKALETVRMMNVAHQRATDYMDLLKEELDEDMFSANEVDFAITLRIIMNCEDEKINTLVLYGPTNTGKSLICR-LTGSVAFAYENLLRKVALMEANQQDLKQILGGEPFEVHIKYQNPDLLERLPVIVTTNEDAAAIEGRCKI-
>Echinococcus_multilocularis_A0A077RCT5
-------------------MNDMNKKFTYQETFRLMADYGQSYNMIVRKALETVRMMNVAHQRATDYMDLLKEELDEDMFSANEVDFAITLRIIMNCEDEKINTLVLYGPTNTGKSLICR-LTGSVAFAYENLLRKVALMEANQQDLKQILGGEPFEVHIKYQNPDLLERLPVIVTTNEDAAAIEGRCKI-
>Echinococcus_multilocularis_U6HLE6
KRQKYIDLAEEIMRRKVRSMNDMNKKFTYQETFRLMADYEQSYNMIVRKALETVRTMNVAHQRATDYMDLLKEELDEDMFSANEVDFAITLRIIMNCEDEKINTLVLYGPTNTGKSLICRLTTGSVAFAYENLLRKVALMEANQQDLKQILGGEPFEVHIKYQNPDLLERLPVIVTTN-DAAAIEGRCKI-
>Echinococcus_multilocularis_A0A068XWU9
KRQKYIDLAEEIMRRKVRSMNDMNKKFTYQETFRLMADYEQSYNMIVRKALETVRTMNVAHQRATDYMDLLKEELDEDMFSANEVDFAITLRIIMNCEDEKINTLVLYGPTNTGKSLICRLTTGSVAFAYENLLRKVALMEANQQDLKQILGGEPFEVHIKYQNPDLLERLPVIVTTN-DAAAIEGRCKI-
>Echinococcus_multilocularis_CDS36723
KRQKYIDLAEEIMRRKVRSMNDMNKKFTYQETFRLMADYEQSYNMIVRKALETVRTMNVAHQRATDYMDLLKEELDEDMFSANEVDFAITLRIIMNCEDEKINTLVLYGPTNTGKSLICRLTTGSVAFAYENLLRKVALMEANQQDLKQILGGEPFEVHIKYQNPDLLERLPVIVTTN-DAAAIEGRCKI-
>Echinococcus_multilocularis_CDI70496
KRQKYIDLAEEIMRRKVRSMNDMNKKFTYQETFRLMADYEQSYNMIVRKALETVRTMNVAHQRATDYMDLLKEELDEDMFSANEVDFAITLRIIMNCEDEKINTLVLYGPTNTGKSLICRTFLGSVAFA------------ANQQDLKQILGGEPFEVSVKYQNPDLLERLPVIVTTNEDAAAIEGRCKI-
>Echinococcus_multilocularis_CDS35594
KRQKYIDLAEEIMRRKVRSMNDMNKKFTYQETFRLMADYEQSYNMIVRKALETVRTMNVAHQRATDYMDLLKEELDEDMFSANEVDFAITLRIIMNCEDEKINTLVLYGPTNTGKSLICRTFLGSVAFA------------ANQQDLKQILGGEPFEVSVKYQNPDLLERLPVIVTTNEDAAAIEGRCKI-
>Echinococcus_multilocularis_U6HA22
KRQKYIDLAEEIMRRKVRSMNDMNKKFTYQETFRLMADYEQSYNMIVRKALETVRTMNVAHQRATDYMDLLKEELDEDMFSANEVDFAITLRIIMNCEDEKINTLVLYGPTNTGKSLICR-LTGSVAFAYENLLRKVALMEANQQDLKQILGGEPFEVSVKYQNPDLLERLPVIVTTNEDAAAIEGRCKI-
>Echinococcus_multilocularis_A0A087VWP3
KRQKYIDLAEEIMRRKVRSMNDMNKKFTYQETFRLMADYEQSYNMIVRKALETVRTMNVAHQRATDYMDLLKEELDEDMFSANEVDFAITLRIIMNCEDEKINTLVLYGPTNTGKSLICR-LTGSVAFAYENLLRKVALMEANQQDLKQILGGEPFEVSVKYQNPDLLERLPVIVTTNEDAAAIEGRCKI-
>Echinococcus_multilocularis_U6H9L2
KRQKYIDLAEEVMRRKVRSMNDMNKKFTYQETVRLMADYGQSYNMIVRKALETVRMMNVAHQRGTDYADLLKEELDEDMFSANAVDFAITLRIIMNCEDEKINTLVLYGPTNTGKSLICKLMT---SFL-----------------------G-------------SVMRRP--------------------
>Echinococcus_multilocularis_U6HF69
KRQKYIDLAEEVMRRKVRSMNDMNKKFTYQETFRLMADYGQSYNMIVRKALETVRLMNVAHQRGTDYADLLKEELDEDRFSANEVDFAITLRIIMNCEDEKINALVLYGPTSTGKSLICKTFLGSVAFAHENLLRKVALMEANQQDLKQILGGEPFEVHIKYQNPDLLERLPVIVTTNEDAAATEGRCKI-
>Echinococcus_multilocularis_CDI70553
KRQKYIDLAEEVMRRKVRSMNDMNKKFTYQETVRLMADYGQSYNMIVRKALETVRMMNVAHQRATDYADLLKEELDEDMFSANAVDFAITLRIIMNCEDEKINTLVLYGPTNTGKSLICKLMTGSVAFAYENLLGKVALMEANQQDLKQILGGEPFEVPVKYQNPDLLERLPVVVTTNEDAAATEGRCKI-
>Echinococcus_multilocularis_CDS35651
KRQKYIDLAEEVMRRKVRSMNDMNKKFTYQETVRLMADYGQSYNMIVRKALETVRMMNVAHQRATDYADLLKEELDEDMFSANAVDFAITLRIIMNCEDEKINTLVLYGPTNTGKSLICKLMTGSVAFAYENLLGKVALMEANQQDLKQILGGEPFEVPVKYQNPDLLERLPVVVTTNEDAAATEGRCKI-
>Echinococcus_multilocularis_U6HA02
---------------------------------------------------------------------------------MNC-------------EDEKINTLVLYGPTNTGKSLICKLMTGSVAFAYENLLGKVALMEANQQDLKQILGGEPFEVPVKYQNPDLLERLPVVVTTNEDAAATEGRCKI-
>Echinococcus_multilocularis_U6HNK1
VKRQKYIDLAEEIMRKVRSMNDMNKKFTYQETFRLMVDCGQYYHMVVRKALETERKMNVAHRRATDFMDLLKEELEEDMFSANAVDFAITLRIIMNCEDEKINTLVLYGPTNTGKSLICKLMTGSVAFAYEDLLRKVALMEANQQDLKQILGGEPFEVHIKYQNPDLLERLPVVVTTNEDAAATEGRCKS-
>Echinococcus_multilocularis_CDS36692
VKRQKYIDLAEEIMRKVRSMNDMNKKFTYQETFRLMVDCGQYYHMVVRKALETERKMNVAHRRATDFMDLLKEELEEDMFSANAVDFAITLRIIMNCEDEKINTLVLYGPTNTGKSLICKLMTGSVAFAYEDLLRKVALMEANQQDLKQILGGEPFEVHIKYQNPDLLERLPVVVTTNEDAAATEGRCKS-
```

>Echinococcus_multilocularis_U6H9C9
---MNC-------------EDEKINTLVLYGPTNTGKSLICKLMTGSVAFAYENLLRKVALMEANQQDLKQILGGEPFEVHIKYQNPDLLERLPVVVTTNEDAAATEGRCKI-
>Echinococcus_multilocularis_U6HFK7
---MEANQQDLKQILGGEPFEVHTKYQNPDLLERLPVVVTTNEDAAATEGRCKI-
>Echinococcus_multilocularis_CDI70562
--M-----------------TSFLEH---GSVAFAYENLLRKVALMEANQQDLKQILGGEPFEVHTKYQNPDLLERLPVVVTTNEDAAATEGRCKI-
>Echinococcus_multilocularis_U6HBG0
KRQKYIDLAEEVMRRKVRSMNDMNKKFTYQETVRLMADYGQSYNMIVRKALETVRMMNVAHQRATDYADLLKEELDEDMFSANAVDFAITLRIIMNCEDEKINTLVLYGPTNTGKSLICKLMTGSVAFAYENLLRKVALMEANQQDLKQILGGEPFEVHIKYQNPDLLERLPVVVTTNEDAAATEGRCKI-
>Echinococcus_multilocularis_U6H9K2
KRQKYIDLAEEVMRRKVRSMNDMNKKFTYQETVRLMADYGQSYNMIVRKALETVRMMNVAHQRATDYADLLKEELDEDMFSANAVDFAITLRIIMNCEDEKINTLVLYGPTNTGKSLICKLMTGSVAFAYENLLRKVALMEANQQDLKQILGGEPFEVHIKYQNPDLLERLPVVVTTNEDAAATEGRCKI-
>Echinococcus_multilocularis_U6HGG3
KRQKYIDLAEEVMRRKVRSMNDMNKKFTYQETVRLMADYGQSYNMIVRKALETVRMMNVAHQRATDYADLLKEELDEDMFSANAVDFAITLRIIMNCEDEKINTLVLYGPTNTGKSLICKLMTGSVAFAYENLLRKVALMEANQQDLKQILGGEPFEVHIKYQNPDLLERLPVVVTTNEDAAATEGRCKI-
>Echinococcus_multilocularis_U6FUW9
KRQKYIDLAEEVMRRKVRSMNDMNKKFTYQETVRLMADYGQSYNMIVRKALETVRMMNVAHQRATDYADLLKEELDEDMFSANAVDFAITLRIIMNCEDEKINTLVLYGPTNTGKSLICKLMTGSVAFAYENLLRKVALMEANQQDLKQILGGEPFEVHTKYQNPDLLERLPVVVTTNEDAAATEGRCKI-
>Echinococcus_multilocularis_CDS35660
KRQKYIDLAEEVMRRKVRSMNDMNKKFTYQETVRLMADYGQSYNMIVRKALETVRMMNVAHQRATDYADLLKEELDEDMFSANAVDFAITLRIIMNCEDEKINTLVLYGPTNTGKSLICKLMTGSVAFAYENLLRKVALMEANQQDLKQILGGEPFEVHTKYQNPDLLERLPVVVTTNEDAAATEGRCKI-
>Echinococcus_multilocularis_U6HA06
-------------------MNDMNKKFTYQETVRLMADYGQSYNMIVRKALETVRMMNVAHQRATDYADLLKEELDEDMFSANAVDFAITLRIIMNCEDEKINTLVLYGPTNTGKSLICKLMTGSVAFAYENLLRKVALMEANQQDLKQILGGEPFEVHTKYQNPDLLERLPVVVTTNEDAAATEGRCKI-
>Echinococcus_multilocularis_CDI70556
--MIVRKALETVRMMNVAHQRATDYADLLKEELDEDMFSANAVDFAITLRIIMNCEDEKINTLVLYGPTNTGKSLICKLMTGSVAFAYENLLRKVALMEANQQDLKQILGGEPFEVHTKYQNPDLLERLPVVVTTNEDAAATEGRCKI-
>Echinococcus_multilocularis_CDS36689
--MIVRKALETVRMMNVAHQRATDYADLLKEELDEDMFSANAVDFAITLRIIMNCEDEKINTLVLYGPTNTGKSLICKLMTGSVAFAYENLLRKVALMEANQQDLKQILGGEPFEVHTKYQNPDLLERLPVVVTTNEDAAATEGRCKI-
>Echinococcus_multilocularis_U6HD97
--MFSANAVDFAITLRIIMNCEDEKINTLVLYGPTNTGKSLICKLMTGSVAFAYENLLRKVALMEANQQDLKQILGGEPFEVHIKYQNPDLLERLPVVVTTNEDAAATEGRCKI-
>Echinococcus_multilocularis_U6H9K6
--MFSANAVDFAITLRIIMNCEDEKINTLVLYGPTNTGKSLICKLMTGSVAFAYENLLRKVALMEANQQDLKQILGGEPFEVHIKYQNPDLLERLPVVVTTNEDAAATEGRCKI-
>Echinococcus_multilocularis_U6HBN8
--MIVRKALETVRMMNVAHQRATDYADLLKEELDEDMFSANAVDFAITLRIIMNCEDEKINTLVLYGPTNTGKSLICKLMTGSVAFAYENLLRKVALMEANQQDLKQILGGEPFEVHIKYQNPDLLERLPVVVTTNEDAAATEGRCKI-
>Echinococcus_multilocularis_U6H9Z7
--MFSANAVDFAITLRIIMNCEDEKINTLVLYGPTNTGKSLICKLMTGSVAFAYENLLRKVALMEANQQDLKQILGGEPFEVHIKYQNPDLLERLPVVVTTNEDAAATEGRCKS-
>Echinococcus_multilocularis_CDI70288
KRQKYIDLAEEVMRRKVRSMNDMNKKFTYQETVRLMADYGQSYNMIVRKALETVRMMNVAHQRATDYADLLKEELDEDMFSANAVDFAITLRIIMNCEDEKINTLVLYGPTNTGKSLICKLMTGSVAFAYENLLRKVALMEANQQDLKQILGGEPFEVHIKYQNPDLLERLPVVVTTNEDAAATEGRCKS-
>Echinococcus_multilocularis_CDS35386
KRQKYIDLAEEVMRRKVRSMNDMNKKFTYQETVRLMADYGQSYNMIVRKALETVRMMNVAHQRATDYADLLKEELDEDMFSANAVDFAITLRIIMNCEDEKINTLVLYGPTNTGKSLICKLMTGSVAFAYENLLRKVALMEANQQDLKQILGGEPFEVHIKYQNPDLLERLPVVVTTNEDAAATEGRCKS-
>Echinococcus_multilocularis_CDI70555
KRQKYIDLAEEVMRRKVRSMNDMNKKFTYQETVRLMADYGQSYNMIVRKALETVRMMNVAHQRATDYADLLKEELDEDMFSANAVDFAITLRIIMNCEDEKINTLVLYGPTNTGKSLICKLMTGSVAFAYENLLRKVALMEANQQDLKQILGGEPFE----------------------------------
>Echinococcus_multilocularis_CDS35653
KRQKYIDLAEEVMRRKVRSMNDMNKKFTYQETVRLMADYGQSYNMIVRKALETVRMMNVAHQRATDYADLLKEELDEDMFSANAVDFAITLRIIMNCEDEKINTLVLYGPTNTGKSLICKLMTGSVAFAYENLLRKVALMEANQQDLKQILGGEPFE----------------------------------
>Echinococcus_multilocularis_U6H948
---DFAITLRIIMNCEDEKINTLVLYGPTNTGKSLICKLMTGSVAFAYENLLRKVALMEANQQDLKQILGGEPFEVHIKYQNPDLLERLPVVVTTNEDAAATEGRCKI-
>Hydatigera_taeniaeformis_LL723621
--M---------------------RTVAVLEPNVNDMKQLFGGEPFEINVKYKPMQYLNRLPVIVTTNEDCAALESRYCQF
>Hydatigera_taeniaeformis_LL725419
--MLCREEIMALNGKPRHDCLGRPLNKEGMDWI-NNLFHSNGHAFCRKMECIMDKKDDKVNTLVLYGPTNTGKSLLCKLMTGTIAFAFENLLRSVGILEANMNDMKQLLGGEEFEVAVKY-----------------KPMQFLK-----
>Hymenolepis_diminuta_LM390786
RIEKTYDLARTLLDRRITHPAQLHEKFSLEEMAQLAVDFGNSYKETVSMIIANLRQSRLKEEKEKPYCELLNRELCDNLFDANGSEFINKLDRVMNRNDSKINSIVLYGPTNTGKSLLCKIMTGTINFAYENLLRSVAILEANVNDMKQLLGGESFEVAVKYKPMQFLHRIPVIITTNEDAAALESRYFQ-
>Hymenolepis_microstoma_CDS32658
RIEKTYDLAKTLLERRITHPSQLNEKFSLDEMAQLMLDFGNSYKETVSMVLANLRQSRLKEERTKSYCELLNRELLECLFKSNGLEFVSRLERVMNKVDNKVNCIVLYGPTNTGKSLLCKIMTGTINFAFENLLRSVAILEANANDMKQLLGGEAFEVSVKYKPMQYLQRLPVIVTTNEDAAALESRYYQ-
>Hymenolepis_microstoma_CDJ13943
RIEKTYDLAKTLLERRITHPSQLNEKFSLDEMAQLMLDFGNSYKETVSMVLANLRQSRLKEERTKSYCELLNRELLECLFKSNGLEFVSRLERVMNKVDNKVNCIVLYGPTNTGKSLLCKIMTGTINFAFENLLRSVAILEANANDMKQLLGGEAFEVSVKYKPMQYLQRLPVIVTTNEDAAALESRYYQ-
>Hymenolepis_diminuta_LM389152
RIDKTYDLARTLLDRRITHPSQLNEHFSLEEMTQLMVDFGNSYKETVSMVLANLRQARLKEERELPYKRLLERELLNQIFHANGFEFCRQVERVLNRQDNKVNTIVLYGPTNTGKSLLCKIMTGTIAFAFENLLRSVAILEGNANDMKQLLGGESFEVAVKYKPMQYLLRLPVIITTN-------------
>Hymenolepis_diminuta_LM388960
---MALAGKSRHDCSGFPFTAEVRSWL-DCLFQSNGLEFCNQLECVMNKRDNKVNTIVLYGPTNIGKSLVCKIATGTIAFAFENLLRSVAILEGNANEMKQLLGGESFEVSVKYKPMQYLPRLPVIITTNEDAAALESRYHQ-
>Hymenolepis_microstoma_CDJ13946
--MQYLQRLPVIITNEYLGCRLPDVDAAAG--KSLLCKVMTGTINFAFENLLRSVAILESNANDMKQLLGGESFEVAVKYKPMQFLPRLPVIITTNEDAAALES-----
>Hymenolepis_microstoma_CDS32661
--MQYLQRLPVIITNEYLGCRLPDVDAAAG--KSLLCKVMTGTINFAFENLLRSVAILESNANDMKQLLGGESFEVAVKYKPMQFLPRLPVIITTNEDAAALES-----
>Hymenolepis_diminuta_LM389639
---------------------------------MSHCYFSDDKRLLEREYLMALSGKPRHDCSREPFIPRIEKWL-ETLFRSNGIEFCKMVEKVLDKKDNKVNTIVLYGPTNTGKSLLCKLMTGTIVFAFENLLRTVAILEANANDMKQLLGGEAFEVAVMYKPMQYLNRLPVVITTNEDAAALESRYYQ-
>Hymenolepis_diminuta_LM390089
RIDKTYDLARMLLDRGVTHAAQLNEKFSLEEMTQLMLDFGNSYKETVSMVLGNLRHARLNEERSMSYKRLLDREYLDTLFRSNGVEFCKTVEKVLDKKDNKVNTIVLYGPTNTGKSLLCKLMTGTIVFAFENLLRTVAILEANANDMKQLLGGEAFEVAVKYKPMQYLNRLPVVITTNEDAAALESRYYQ-
>Dicrocoelium_dendriticum_LK428994
RTKRTNLIDERIEVHRCTHLKELKRCLTRKQRLTIYNEFGKQWEESAIMCIEAFNEELFERQTHVSFEEYMLENNHDNLLAANKDKFCKAIHEIMNKKIDRLNCLCIEGPTTTGKSLVLKMICGTVQFFLQNLVKSIALMEATVNDFKELLGGSPFDIHVKHSPDVTLNRLPVLISTNHDAAAIYERCHT-
>Dicrocoelium_dendriticum_LK459200
----------------------MKRCLTRKQRLSIYNEFGMQWEESAKMCIEAFNEELFERQTNISFEEYMQENNHDNLLAANKDKFCKAIHEVMNKKIDRLNTLCIEGPTTTGKSLVLKMICGTVQFFLQNLVKSVALMEATVNDFKELLGGSPFDIHVKHSPDVTLSRLPVLISTNHDAAAIY------
>Schistosoma_mattheei_LM181082
---QKFGINTLCIEGPTTTGKTLILKLITGTVKFFLQNLNKSVALMEVNVNDFKQLLGGSSLDIHIKHQSDVTLQRIPVLISTNSDKKAIYKRCITY
>Schistosoma_margrebowiei_LL888002
RVQCGEHLMDLVTLYNVTHLQQLKMKLSAKDRLNLYYEYGKQWEETAQLCIDVYNETNIKQQMCVPWVTWVSTNHHETFFAANEKQFLNDIQTIIDKTKLRVNTFCLEGPTTTGKTLILKLIVGTVQFFLQNLNKSVALMEITVNDFKELLGGSPFDIHITHQPDVTLPRIPVLISTNSDKDAIYKRCITY
>Taenia_multiceps_JR929085
---NVAMGMRTIPWKLGASGSIRCYCITTF------------LLLYTKVVDVVDEKMDKVNMLCFYRTTNAGKSLLAGLITAAIAFQFDNLLKTGALME--
>Schistosoma_mansoni_XP_002571349
RKQRTDLILDMVRLNDTRQMDAFENSLSTQQRLDLYNDFGNQWQEVAKLCIKIYNEELVRAQIDTPYEQYEGSHDEDMLMEKNDKVFCASVSTIMNKKVKRVNTLCLEGPTTTGKSLLLKLICGTVQFFLQNLLKTVALMEITVQDFKLLLGGEPLDIHVKHQDDCRLPRIPVLISTNHDKDALYNRTHY-
>Opisthorchis_viverrini_XP_009173661
RKQRTDLILDMVRLNDTRQMDAFENSLSTQQRLDLYNDFGNQWQEVAKLCIKIYNEELVRAQIDTPYEQYEGSHDEDMLMEKNDKVFCASVSTIMNKKVKRVNTLCLEGPTTTGKSLLLKLICGTVQFFLQNLLKTVALMEITVQDFKLLLGGEPLDIHVKHQDDCRLPRIPVLISTNHDKDALYNRTHY-
>Schistosoma_mansoni_CCD58408
RKQRTDLILDMVRLNDTRQMDAFENSLSTQQRLDLYNDFGNQWQEVAKLCIKIYNEELVRAQIDTPYEQYEGSHDEDMLMEKNDKVFCASVSTIMNKKVKRVNTLCLEGPTTTGKSLLLKLICGTVQFFLQNLLKLLPSWK--
>Opisthorchis_viverrini_XP_009170655
RKRRRELMKGLIEAYDARSYNEFYMAMSCDDRDDIYDEYGPQWKETAEHSINNYIAGMLSKQLAMRFEEILMTNYHDQLMSANKQQFLTDLTLVMNKVCTRKNASVIEGPTTTGKTLFVKLIAGTVTKPATTTATTTTVAPQRLDTQEDESYGPNLAVHAQSLFGIHYYKTDVTVTRKQGGDNFRDNLVAV
>Amblyomma_americanum_GAGD01007016
--MLLGGDSLSVNKKFEDYVEIYRTPIIMLTNNRDPVWTDRCLFY
>Opisthorchis_felineus_GBJA01006694
RRLVDDVLMNLVEANDARSFNEFYMSLTFEQTNDIYAEYGPQWKHTAKHCIANYTKKILKHQQTSRFEDIIRTNNHDLLFHANKRDFLVNLTQIMNKDIDGRNAFVIEGPTTTGKTLLVKLIAGTVQCFLMKLLKALALVELTVNHFKALLGGNAFDIRVKHQKDERLTRLPVLITTNTDAKAIKERCFI-
>Clonorchis_sinensis_GAA57952
RKRRLDLLRNLVETYDARSFNELNLALTYDERDDIYGEYGSQWKETAEHCIQNYTMRILVEQQTNRFEDHIRTNSHDLLLHVNRQNFLTDLTRVMNKQIDRKNAFVLEGPTTTGENIIRKTHCGQLHIW-----DRTEIWR-------------------------VLPNEP------------TE-----
>Clonorchis_sinensis_GAA32750
RKRRLDLLRNLVETYDARSFNELNLALTYDERDDIYGEYGPQWKETAEHCISNYTVKILIEQQTSRFEDHIRTNSHDLLLHVNRQDFLCNLTKIMNKQIDRKNAFVIEGPTTTGKTLFVKLIAGTVQFFLMNLLKALALMELTVNDFKELLGGNAFDIHVKHQKDERLTRLPVLITTNNSRKFYLDQLSHA
>Clonorchis_sinensis_GAA40146
RKRRLDLLRNLVETYDARSFNELNLALTYDERDDIYGEYGPQWKETAEHCIQNYTMKILLEQQTSRFEDHIRTNSHDLLLHVNRQEFLTNITKIMNREIDRKNAFVIEGPTTTGKTLFVKLIAGTVQFFLMNLLKALALMELTVNDFKELLGGNAFDIHVKHQKDERLTRFPVLITTNNDDKAIKERCFYY
>Clonorchis_sinensis_GAA56553
RKRRLDLLRNLVETYDARSFNELNLALTYDERDDIYGEYGPQWKETAEHCIQNYTMRILVEQQTSRFEDHIRTNSHDLLLFVNRQQFLADLTRVMNKEVDRKNAFVLEGPTTTGKTLFVKLIAGTVQFFLMNLLKALALMELTVNDFK---
>Clonorchis_sinensis_GAA57641
RKRRLDLLRSLVETYDARSFNELNLALTYDERDDIYGEYGPQWKETAEHCIQNYTMRILVEQQTSRFEDHIRTNSHDLLLFVNRQQFLTDLTRVMNKQIDRKNAFVLEGPTTTGKTLFVKLIAGTVQFFLMNLLKALALMELTVNDFKELLGGNAFDIHVKHQKDERLTRLPVLITTNNDGKAIKERCFY-
>Clonorchis_sinensis_GAA57638
RKRRLDLLRNLVETYDARSFNELNLALTYDERDDIYGEYGPQWKETAEHCIQNYTMRILVEQQTSRFEDHIRTNSHDRLLFVNRQEFLADLTRVMNKQVDRKNAFVLGGPTTTGKTLFVKLIAGTVQFFLMNLLKALALMELTVNDFKELLGGNAFDIHVKHQKDERLTRLPVLITTNNDGKAIKERCFY-
>Clonorchis_sinensis_GAA57639
RKRRLDLLRNLVDTYDARSFNELNLALTYDERDDIYGEYGPQWKETAEHCIQNYTMRILVEQQTSRFEDHIRTNSHDRLLFVNRQQLLADLTRVMNKQVDRKNAFVLEGPTTTGKTLFVKLIAGTVQFFLMNLLKALALMELTVNDFKELLGGNAFDIHVKHQKDERLTRLPVLITTNNDGKAIRERCFYY
>Clonorchis_sinensis_GAA57954
RKRRLDLLRNLVETYDARSFNELNLALTYDERDDIYGEYGPQWKETAEHCIQNYTMRILVEQQTSRFEDHIRTNSHDRLLFVNRQKFLADLTRVMNKQVDRKNAFVLEGPTTTGKTLFVKLIAGTVQFFLMNLLKALALMELTVNDLKELLGGNAFDIHVKHQKDERLTRLPVRITTNNDGKVIKERCFY-
>Clonorchis_sinensis_GAA57640
RKRRLDLLRNLVETYDARSFNELNLALTYDERDDIYGEYGPQWKETAEHCIQNYTMRILVEQQTSRFEDHIRTNSHDRLLFVNRQKFLCDLTRVMNKQVDRKNAFVLEGPTTTGKTLFVKLIAGTVQFFLMNLLKALALMELTVNDFKELLGGNAFDIHVKHQKDERLTRLPVLITTNN------------
>Opisthorchis_felineus_GBJA01005901
--MIRPAKRIMIEAYDARSYNELYMSLTYDETDDIYAEYGPQWKETAEHCIANYIKKILISQQTSRFEDVLRSNNHDLLFYVNKQEFLIDITRIMNKEVDRTNAFVIEGPTTTGKTLFVKLIAGTVQFFLMNLLKALALMELTVNYFKELLGGNPFDIHVKHQKDERLNRLPVLITTNNDGKAIKERCFT-
>Opisthorchis_felineus_GBJA01002124
RKRRLDLMRELVTAYDARSYNELYMKLSVEHTDDIYAEYGPTWKETADHAIANYCKKIIIDQQTMTFDEILNSNHHDQLMAVNNKEMLHSLTLVMNKTMKRKNAFVIEVPTTTGKTLFVKLIAGTVPFFLMNLLKTLALMELTVNDFKELLGGNPFDIHVKHQKDERLDRLPVLITTNNDAKAILERCFY-
>Opisthorchis_felineus_GBJA01002120
RKRRLDLMRELVTAYDARSYNELYMKLSVEHTDDIYAEYGPTWKETADHAIANYCKKIIIDQQTMTFDEILRSNHHDQLMLVNNKDMLHALTLVMNKTMKRKNAFVIEGPTTTGKTLFVKLIAGTVQFYLMNLLKTLALMELTVNDFKELLGGNPFDIHVKHQKDERLERLPVLITTNNDAKAILERCFY-
>Opisthorchis_felineus_GBJA01002117
-KRRLDLMRELVTAYDARSYNELYMKLSVEHTDDIYAEYGPTWKETADHAIANYCKKIIIEQQTMTFDEILRSNHHDQLMLVNNKEMLHSLTLVMNKTMKRKNAFVIEGPTTTGKTLFVKLIAGTVQFFLMNLLNKTLGFS--
>Opisthorchis_viverrini_XP_009177542
GKRRRELMKGLIEAYDARSYNEFYMAMSCEDRDDIYDEYGPQWKETAEHSINNYIAGMLAKQLAMRFEEVLMGNFHDQLMWVNRKQFLIDLTQIMNKAYKRKNAFVIEGPTTTGKTLFVKLIAGTVQFFLMNLLKSLGLMELTVNDFKELLGGNPFDIHVKHQKDERLQRLPVLITTNNDGKAILERCFY-
>Opisthorchis_felineus_GBJA01002122
---MSRFPDSISLCMDRSDNHNRECQHPKNTIDGEQDLLFQVNKQTFLCNITRIMNKEIDRKNAFVIEGPTTTGKTLFVKLIAGTVQFFLMNLLKALALMELTVNDFKELLGGNPFDIHVKHQKDERLNRLPVLITTNNDGKAIKERCFA-
>Opisthorchis_viverrini_XP_009176824
RKRRRELMKGLIEAYDARSYNEFYMAMSCDDRDDIYDEYGPQWKETAEHSINNYIAGMLSKQLAMRFEEILMTNYHDQLMAANKQQFLTDLTLVMNKVCTRKNAFVIEGPTTTGKTLFVKLIAGTVQFFLMNLLKALALMELTVNDFKELLGGNPFDIHVKHQKDERLQRLPVLITTNNDGKAILERCFY-
>Opisthorchis_viverrini_XP_009170634
-------MKGLIEAYDARSYNEFYMAMSCDDRDDIYDEYGPQWKETAEHSINNYIAGMLSKQLAMRFEEILMTNYHDQLMSANKQQFLTDLTLVMNKVCTRKNAFVIEGPTTTGKTLFVKLIAGTVQFFLMNLLKALALMELTVNDFKELLGGNPFDIHVKHQKDERLQRLPVLITTNNDGKAILERCFY-
>Opisthorchis_felineus_GBJA01010281
---MTFAEILNSNHHSRTCQHPGNTIDGDQLMQVNNKDILHSLTLIMNKSLKRKNAFVIEGPTTTGKTLFVKLIAGTVQFFLMNLLKTLALMELSVNDFKELLGGNPFDIHVKHQKDELHYGTH-------RRSFWCW-----
>Opisthorchis_viverrini_XP_009178033
RKRRLDLMRELVRTYDARSFNELYKRLSVQDTDDIYAEYGPTWKETAEHSISNYCKEIILEQETMTFEQILTSNHHDELIRVNNRELLVSLTAIMNKLCTRKNAFVIEGPTTTGKTLFVKLIA-----------------------------G-----TVQRSGDH-------------------------
>Opisthorchis_felineus_GBJA01008850
RKRRLDLMRELVAAYDARSYNELYMRLSVEHTDDIYAEYGPTWKETADHAITNYCKKIIIEQQTMTFEEILRSNHHDRLMLVNNRELLTSLTLVMNKSMNRKNAFVIEGPTTTGKTLFVKLVAGTVQFFLMNLLKTLALMELTVNDFKELLGGNPFDIHVKHQKDERLERLPVLITTNNDAKAILERCFY-
>Opisthorchis_viverrini_XP_009177530
INKDLTIAGLLSTWDRYVLDPEFREQLSQVLRGIPWDPNDIKYQAHLMWTHATRDVPPYTGNMIDVLQTRRKNYPKDTQEPKTVAKHAQSLFGIHYKTRKQHLTLAQYKPADTGWSINMNFRDGSIPYAAKDSLDKDVFVNKTSDEGLLGLADKTFHQTVYYQMNCLKQAVPAFDNARGERKPVKTMNVKV
>Opisthorchis_viverrini_XP_009177798
---MNK-------------LCTRKNAFVIEGPTTTGKTLFVKLVAGTVQFFLMNLLKTLALMELTVNDFKELLGGNAFDIHVKHQKDERLDRLPVLITTNNDGKAILERCFYF
>Opisthorchis_viverrini_XP_009177702

RKRRLDLMRELVRTYDARSFNELYKRLSVQDTDDIYAEYGPTWKETAEHSISNYCKEIILEQETMTFEQILNSNHHDQLIQVNNRELLVCLTSVMNKLCTRKNAFVIEGPTTTGKTLFVKLVAGTVQFFLMNLLKTLALMELTVNDFKELLGGNPFDIHVKHQKDERLERLPVLITTNN-----------
>Opisthorchis_viverrini_XP_009177942
-------MRELVRSYDARSFNELYKRLSVQDTDDIYAEYGPTWKETAEHSIANYCKEIILEQETMTFEQILRSNHHDELIRVNNRDLLVALTAVMNKLCTRKNAFVIEGPTTTGKTLFVKLVAGTVQFFLMNLLKTLALMELTVNDFKELLGGNPFDIHVKHQKDE------------------------
>Bithynia_siamensis_GAQQ01009056
---MCTRKNAFVIEGPTTTGKTLFVKLVAGTVQFFLMNLLKTLALMELTVNDFKELLG---------------------------------------
>Opisthorchis_viverrini_XP_009177808
RKRRLDLMRELVRSYDARSFNELYKRLSVQDTDDIYAEYGPTWKETAEHSIANYCKEIILEQETMTFEQILNSNHHDQLIRVNNRELLACLTSVMNKLCTRKNAFVIEGPTTTGKTLFVKLVA---NYE------------LTVNDFKELLGGNPFDIHVKHQKDERLDRLPVLITTNNDGKAILER----
>Opisthorchis_viverrini_XP_009177971
---MKLLNKTLALME------------------EPRITQ---------------------LTVNDFKELLGGNPFDIHVKHQKDERLERLPVLITTNN-----------
>Moniezia_expansa_JL291017_1
--VLQGPSNTGKSLLAKLIVATVNFIFQNLLKTAALMEATVNDFKQLLGGERMEIGIKHRDREWLERVPIICTTNDDCQAIQNRCVY-
>Moniezia_expansa_JL291017_2
--VLQGPSNTGKSLLAKLIVATVNFIFQNLLKTAALMEATVNDFKQLLGGERMEIGIKHRDREWLERVPIICTTNDDCQAIQNRCVY-
>Echinostoma_caproni_LL285499
RKARVDLIRELVETYDARTLAELKDALSYDDRLNLYAEHGPSWKETAELACEAYVEKLRKDQETTSLHEYIARNNHDRLLAVNNNKFLTQLTDIMNKKENRVNAFVIQGPTTTGKFQYSSSHRGELNSPWRPLPQGSCAYGINGQRLQRTPGRKTIRYETPTRCPAQTPTRPHFYQYRHGREGHQGAMFL-
>Echinostoma_caproni_LL274983
PDKLWTFIYRGPSPNFTIHTRINVLWADHGDHYHFVFKHPNNKRRTITRIIQAGNLDTNQSMSIFSTCQPVINWEKAYLVRHGRQHLATDARSTTR-AEQDCATLLRNDRAQ-GQKFTNLARVDLIALSYDDRLHGPSWKEAYVERLRKDQETTSLHEYIARNNHNRTCQKPTSTTAGC-----------
>Echinostoma_caproni_LL284665
PDKLWTFIYRGRAPTFNSHTRISLLWADHEDHYHFVFKHPNNKKRTIQRIIQAGNLDTAQSVSIFSTCQPVINWDKAYLVRTNRQSLANDARSTTR-AEQDCATLLRNDRAQ-GQKFTNLARVDLIALSYDDRLHGPSWKEAYVERLRKEQETTSLHEYISRNNHSRTCQKP---RSTEEGCRWLDRLLAV
>Echinostoma_caproni_LL286487
RKARVDLIKELVETYDARTLAELKDALSYDDRLNLYAEHGPSWKETAELACEAYVERLRKEQETTTLHEYISRNNHDRLLAVNNNRFLTQLTNIMNKKEQRINAFVIQGPTTT-GKFLCS---SHLSSHILDAKGELHLWNFAILSHEFAEQG---------CRPH--GRAS-------NHPAHGE-----
>Scylla_olivacea_GDRN01037122
---MQKINTLVIKGPTGTGKTLTLATLLGTIQFHLQNLLRNFALFEATVDEYKLLLEGFQFEINVKNSDMEQLHRIPIFISTNRDGQALQSRCKTF
>Penaeus_monodon_AAM94165
NSAEYDYLRHLVKSKAARTVQELVNKLDDEEYKQLWTRTRGQYKDKLRGILTYYNNKKKSSQSQLSLITIYKNISKKYMFANNDPEILAWIIIVADKKLDKINTLVLQGPTGTGKSLTIGALLGLVTFHLQNLIKSYALFEITVDDFKLLFEGSDLEVNIKHQESEIMGRIPIFISTNKDGKALQTRTKT-
>Penaeus_monodon_endogenous_virus_DQ228358_
NSAEYDYLRHLVKSKAARTVQELVNKLDDEEYKQLWTRTRGQYKDKLRGILTYYNNKKKSSQSQLSLITIYKNISKKYMFANNDPEILAWIIIVADKKLDKINTLVLQGPTGTGKSLTIGALLGLVTFHLQNLIKSYALFEITVDDFKLLFEGSDLEVNIKHQESEIMGRIPIFISTNKDGKALQTRTKT-
>Penaeus_monodon_penstyldensovirus_1_GQ411199
NSAEYDYLQHLVKTKSARTVQELVNKLDDEEYKQLWTRTRGQYKDKLRGILTYYNNKKKSNQSQLSLITNLQNISKKYMLANNDPEILAWIIIVADKKLDKINTLVLQGPTGTGKSLTIGALLGLVTFHLQNLIKSYALFEITVDDFKLLFEGSDLEVNIKHQESEIMGRIPIFISTNKDGKALQTRTKTF
>Penaeus_monodon_penstyldensovirus_2_AY124937
NSAEYDYLRHLVKTKSARTVQELVNKLDDEEYKQLWTRTRGQYKDKLRGILTYYNNKKKSNQSQLSLITNLQNISKKYMLANNDPEILAWILIVADKKLDKINTLVLQGPTGTGKSLTIGALLGLVTFHLQNLIKSYALFEITVDDFKLLFEGSDLEVNIKHQESEIMGRIPIFISTNKDGKALQTRTKT-
>Penaeus_stylirostris_penstyldensovirus_2_GQ475529
NSAEYDYLQHLVKTKSARTVQELVNKLDDEEYKQLWTRTRGQYKDKLRGILTYYNNKKKSNQSQLSLITNLQNISKKYMLANNDPEILAWIIIVADKKLDKINTLVLQGPTGTGKSLTIGALLGLVTFHLQNLIKSYALFEITVDDFKLLFEGSDLEVNIKHQESEIMGRIPIFISTNKDGKALQTRTKT-
